# Palladium-catalyzed intermolecular transthioetherification of aryl halides with thioethers and thioesters†

**DOI:** 10.1039/c9sc05532k

**Published:** 2020-01-22

**Authors:** Yahui Li, Gao Bao, Xiao-Feng Wu

**Affiliations:** Key Laboratory of Agri-Food Safety of Anhui Province, School of Resources and Environment, Anhui Agricultural University Hefei 230036 China Yahui.Li@ahau.edu.cn; Leibniz-Institut für Katalyse e.V. an der Universität Rostock Albert-Einstein-Straße 29a 18059 Rostock Germany Xiao-Feng.Wu@catalysis.de

## Abstract

Functional group transfer reactions are an important synthetic tool in modern organic synthesis. Herein, we developed a new palladium-catalyzed intermolecular transthioetherification reaction of aryl halides with thioethers and thioesters. The synthetic utility and practicality of this catalytic protocol are demonstrated in a wide range of successful transformations (>70 examples). This catalytic protocol is applicable in carbonylative coupling processes as well, and the first example of carbonylative methylthioesterification of aryl halides has been achieved. Notably, this work also provides an approach to using natural products, such as methionine and selenomethionine, as the functional group sources.

## Abstract 2

Functional group transfer reactions are an important synthetic tool in modern organic synthesis.

## Graphical abstract

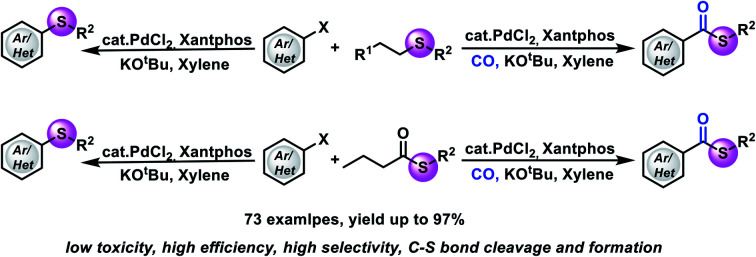

Functional group transfer reactions have become an indispensable tool in organic synthesis.^1^ Representative examples such as metathesis^2^ and transfer hydrogenation^3–6^ have had enormous applications in polymer materials and pharmaceuticals. Recently, the limit of transfer hydrogenation has been extended successfully. Besides hydrogen, other small molecules (H_2_/CO and RCOH) can also be used in these processes.^7–12^ In 2016, Morandi and co-workers reported an elegant Ni-catalyzed reversible transfer hydrocyanation reaction between alkyl nitriles and alkenes under mild conditions.^13*a*^ Later on, they reported their new achievement of palladium-catalyzed C–S bond metathesis (Fig. 1B).^13*b*^ In 2018, the research groups of Arndtsen and Morandi reported their achievement of palladium-catalyzed metathesis between aroyl chlorides and aryl iodides, respectively.^14^ By using Pd_2_dba_3_/Xantphos as the catalyst system, new acid chlorides can be produced effectively. Among all the carbon-heteroatom bond transfer reactions, the formation of carbon–sulfur and carbon–selenium is an attractive target because a broad range of pharmaceuticals, biological molecules, and agrochemicals contain these bonds (Fig. 1A).^15,16^ However, several challenges need to be overcome in order to realize this hypothesis, because compared with C–C bond metathesis, the displacement of dative ligands by thiols and thiolates usually deactivates late transition metals due to the formation of ionic thiolate complexes or bridging thiolate complexes that undergo slow reductive elimination.^17^

**Fig. 1:**
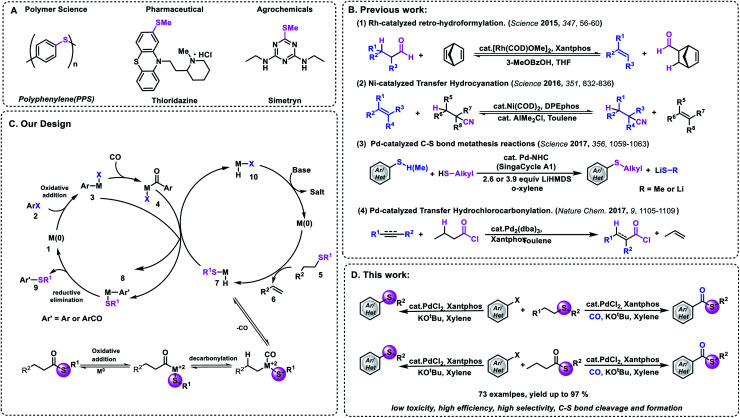
Development of catalysts and design of thiomethylation and carbonylative thiomethylation of aryl halides. (A). Selected examples of compounds containing a C–S bond. (B) Development of transfer catalysis. (C) Our transfer strategy. (D) This work.

On the other hand, existing examples of transfer reactions usually occur between two components. Therefore the development of multicomponent transfer reactions remains an interesting area for exploration. Transition metal-catalyzed carbonylation reactions are one of the most potent tools for the synthesis of carbonyl-containing chemicals.^18–21^ In these transformations, carbon monoxide (CO) can be used as one of the cheapest and abundant C_1_ building blocks; meanwhile the carbon chains of the parent molecules can also be increased. However, most of the known procedures need ready-made chemicals as the nucleophile. More specifically, amines and alcohols are the more frequently explored nucleophiles. Thio-related reagents are much less explored, due to their odor and/or gas properties. Consequently, the development of new processes to transfer more challenging nucleophiles remains an important goal. For example, methyl thioesters are important intermediates and building blocks in nature and in organic synthesis.^22,23^ However, to the best of our knowledge, there is no example that uses the carbonylation reaction in the synthesis of aryl methylthioesters. This may be due to the fact that methyl mercaptan is a flammable and highly toxic gas and can explode after mixing with air.

With this background, we wish to report here the first example of palladium-catalyzed thiomethylation and carbonylative thiomethylation of an aryl halide by using an alkyl sulfide or methyl thioester as a convenient methylthiolating reagent (Fig. 1D). Inspired by previous work,^13^ we hypothesized that a simple alkyl sulfide bearing β-H hydrogens could possibly be employed as a methylthiolating reagent (Fig. 1C). We envisaged that a challenging sequence of C–SMe bond oxidative addition followed by β-H elimination could be mediated by a metal catalyst and give the important intermediate H-M-SMe species **7**. Subsequently, the organometallic nucleophile (H-M-SMe) undergoes transmetallation with ArCO-M-X **4** or Ar-M-X **3** to form the desired thioester or aryl methyl sulfide after reductive elimination. And the active M^0^ species **10** could be regenerated after the reaction between H-M-I and a base.

To prove that the hypothesis is valuable, control experiments have been performed. A palladium catalyst was chosen as the metal catalyst because Pd(0) complexes are very active species in the formation and cleavage of C–S bonds. As shown in Fig. 2A, with 5 mol% PdCl_2_ and Xantphos, 8% yield of 1-dodecene could be obtained. The result shows that the catalytic cycle can be achieved by using a palladium salt as the catalyst.

**Fig. 2:**
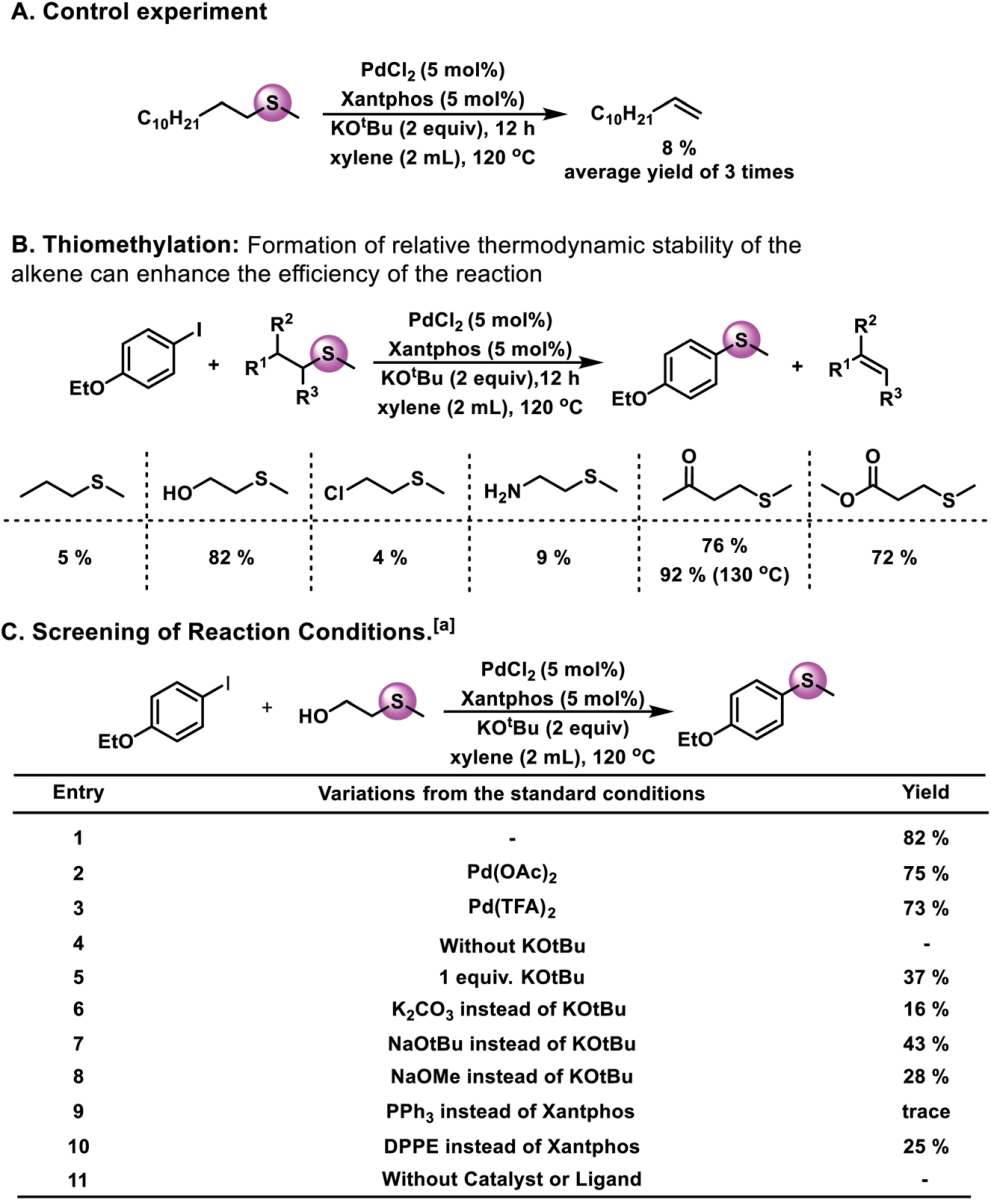
Control experiment and condition selection. (A) Control experiment. (B) Reagent optimization for thiomethylation. (C) Condition selection: ^a^reaction conditions: **1a** (0.2 mmol), **2** (0.6 mmol), [Pd] (5 mol%), ligand (5 mol%), base (0.4 mmol), xylene (2 mL), 120 °C, and 12 h. ^b^Yield was determined by GC using *n*-dodecane as the internal standard.

Subsequently, a range of alkyl sulfides were tested as potential methylthiolating reagents (Fig. 2B). And the experimental results showed that 5%, 82%, 9%, 76%, and 72% yields of the desired product were obtained when methyl(propyl)-sulfide, 2-(methylmercapto)ethanol, 2-(methylthio)ethylamine, 4-(methylthio)butan-2-one and methyl 3-(methylthio)-propanoate were used in the reaction independently. Here, the existence of a suitable coordinating atom in the reagent, such as oxygen, might be favorable for the target reaction. Additionally, the possibility of further transforming the eliminated alkene can promote the desired reaction. For example, when 2-(methylmercapto)ethanol was used as the methylthiolating reagent, the eliminated product, ethanol, could be transformed into acetaldehyde, which could drive the reaction towards the right.

After this, we selected 1-ethoxy-4-iodobenzene and 2-(methylmercapto)ethanol as the model substrates to establish this thiomethylation procedure. Upon variation of the reaction conditions, different yields could be obtained (Fig. 2C). Among the different metal catalyst precursors, PdCl_2_ showed the best results (82% GC yield; 80% isolated yield). Notably, no significant decrease of the yield was obtained when we used Pd(OAc)_2_ and Pd(TFA)_2_ instead of PdCl_2_. Moreover, without a catalyst or ligand, no desired product could be detected. The amount of KO^*t*^Bu plays an important role in this reaction. Only 37% yield of the desired product was obtained if we decreased the loading of KO^*t*^Bu from 2 equivalents to 1 equivalent. The properties of the base seem to have a strong influence on this transformation; when NaO^*t*^Bu, NaOMe, or K_2_CO_3_ were used instead of KO^*t*^Bu, the yield of the target product decreased significantly. Next, the effects of different ligands were studied. The reaction with PPh_3_ or DPPE resulted in decreased yields. Extensive evaluation revealed that a reaction in the presence of 5 mol% PdCl_2_ and Xantphos together with 2 equiv. of KO^*t*^Bu at 120 °C can give the desired product in 82% yield.

In order to prove the synthetic potential of this methodology, testing of different aryl iodides was also conducted under our standard conditions. Good yields of the desired aryl methyl sulfides can be produced by reacting 1-iodo-3,5-dimethylbenzene and 1-(*tert*-butyl)-4-iodobenzene with 2-(methylmercapto)ethanol (72% and 76% yield, respectively). Substrates bearing electron-donating or electron-withdrawing groups reacted well and gave the desired product in good to excellent yields. Also, good to excellent yields of the desired aryl methyl sulfides can be obtained from the corresponding *ortho*-, *meta*-, and *para*-substituted aryl iodides. Substrates bearing different functional groups with oxygen, nitrogen and sulfur atoms such as 4-iodo-*N*,*N*-dimethylaniline, (4-iodophenyl)(methyl)sulfide, and 1-iodo-4-phenoxy-benzene were well tolerated and the corresponding products were obtained in 71%, 88%, and 82% yields, respectively. Additionally, a heteroaromatic ring can also proceed in this reaction and yield 6-(methylthio)quinoline in 72% yield. Furthermore, in order to further demonstrate the synthetic utility of this strategy, we also performed an experiment that uses 2-(phenylthio)ethanol to replace 2-(methylmercapto)ethanol under our standard conditions. The arylthio group can also be transferred to aryl iodide, and it gave the corresponding thioether in 62% yield. To clearly illustrate the synthetic power of the reaction, a gram-scale experiment has also been performed and 70% of the desired product was obtained. Methionine is an essential amino acid in humans and plays a critical role in metabolism and health.^24,25^ Additionally, it is the main source of active methyl, sulfur and methylthio in the body. Herein, we implement a synthetic version that features the transfer of a methylthio group from methionine to aryl iodides. As shown in Fig. 3, good yield of the desired aryl methyl sulfide can be produced by reacting 1-iodonaphthalene with methionine. Another important element, selenium, can also participate in this reaction; the methyl selenization of 1-ethoxy-4-iodobenzene gave the corresponding product in good yield. We next aimed to evaluate the universality of our process of carbonylative thiomethylation of aryl halides. We found that the tested aryl halides can be effectively transformed in general. Good to excellent yields of the desired aryl methylthioesters can be obtained. The reaction also showed good functional group tolerances.

**Fig. 3:**
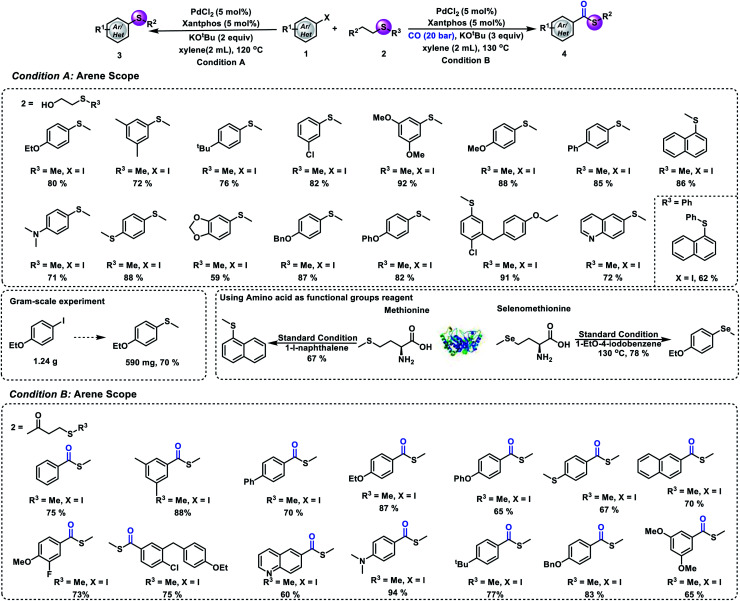
Substrate scope. (A) Thiomethylation of an aryl halide and 2-(methylmercapto)ethanol. (B) Carbonylative thiomethylation of an aryl halide and 4-(methylthio)butan-2-one. Condition A: **1** (0.2 mmol), **2** (0.6 mmol), PdCl_2_ (5 mol%), Xantphos (5 mol%), KO^*t*^Bu (0.4 mmol), xylene (2 mL), 120 °C, 12 h, and isolated yield. Condition B: **1** (0.2 mmol), **2** (0.6 mmol), PdCl_2_ (5 mol%), Xantphos (5 mol%), KO^*t*^Bu (0.6 mmol), xylene (2 mL), 20 bar CO, 130 °C, and 16 h.

Having successfully developed a method for transferring a methylthio group from a methyl sulfide to aryl halides, we next aimed to extend the scope of the methylthio source. Despite the fact that methylthioesters provide meaningful chemoselectivity in the synthesis of biomolecules and Fukuyama coupling, they have rarely been used as a methylthio source. Hence, we turned our attention to methylthioesters. We envisaged a mechanism that involves oxidative addition, decarbonylation and β-hydride elimination (Fig. 1C).

After a small modification of the reaction conditions, several aryl halides and phenyl triflate were selectively thiomethylated and gave the corresponding products in good to excellent yields. As shown in Fig. 4, good to excellent yields of the desired aryl methyl sulfides can be obtained from the corresponding substrates bearing either electron-deficient or electron-donating groups. *ortho*-Substituted aryl halides reacted well under our reaction conditions. Heterocycles such as unprotected indole, benzothiazole and quinoline were well tolerated under our conditions. Notably, 4-bromophenylboronic acid, pinacol ester also reacted well in our reaction, which provides useful possibilities for further synthetic transformations. For the scope of thioesters, as shown in Fig. 4, cyclohexanethio-, 1-decanethio- and arylthio- can also be tolerated in our process and gave the desired products in good to excellent yields. After proving the compatibility of aryl halides and thioesters for this methodology, we became interested in carbonylative thiomethylation. Different aryl halides were tested under our standard conditions successively. The corresponding carbonylation products were produced in good to excellent yields with excellent selectivity.

**Fig. 4:**
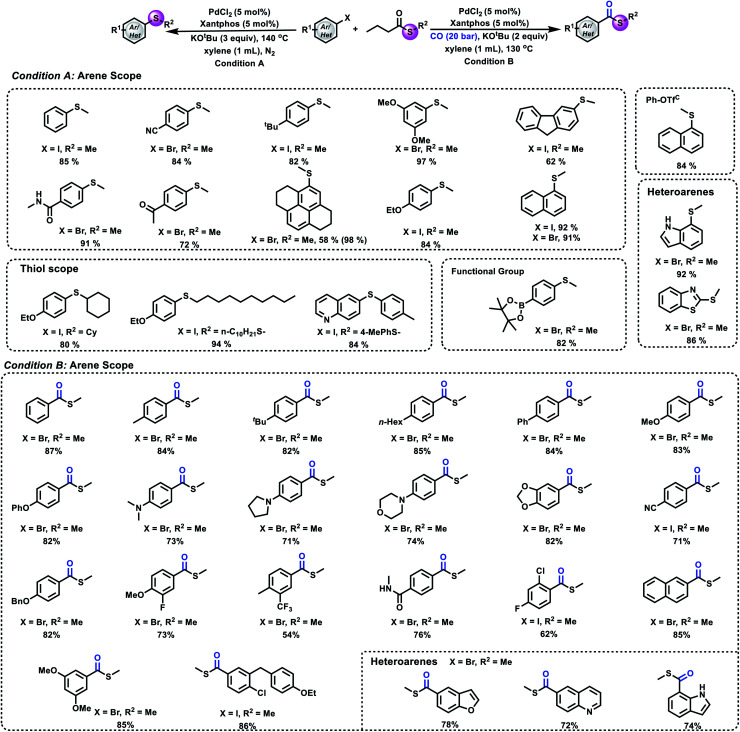
Substrate scope. (A) Thiomethylation of an aryl halide and *S*-methyl butanethioate. (B) Carbonylative thiomethylation of an aryl halide and *S*-methyl butanethioate. Condition A: **1** (0.2 mmol), **2** (0.6 mmol), PdCl_2_ (5 mol%), Xantphos (5 mol%), KO^*t*^Bu (0.6 mmol), xylene (1 mL), 140 °C, N_2_, 12 h, and isolated yield. Condition B: **1** (0.2 mmol), **2** (0.6 mmol), PdCl_2_ (5 mol%), Xantphos (5 mol%), KO^*t*^Bu (0.6 mmol), xylene (1 mL), 20 bar CO, 130 °C, and 16 h. C: using NaOMe as the base.

In conclusion, a novel palladium-catalyzed intermolecular transthioetherification reaction of aryl halides has been developed. With alkyl sulfides, thioesters and even natural products as convenient functional reagents, different aryl sulfides and thioesters were obtained. Overall, the broad scope, excellent functional group compatibility, and high efficiency allow for efficient and safe synthesis of related chemicals in the pharmaceutical industry and in laboratories.

## Conflicts of interest

The authors declare no competing financial interests.

## Supplementary Material

SC-011-C9SC05532K-s001
