# Supplementary material for: Palladium-catalyzed intermolecular transthioetherification of aryl halides with thioethers and thioesters
Source: Chem Sci. 2020 Jan 22;11(8):2187–92. doi: 10.1039/c9sc05532k (PMC8150098; doi:10.1039/c9sc05532k)
Supplement: SC-011-C9SC05532K-s001 [file SC-011-C9SC05532K-s001.pdf]

Electronic Supplementary Material (ESI) for Chemical Science.
This journal is © The Royal Society of Chemistry 2020

Supporting Information

General Remarks

Most of chemicals were purchased from Sigma-Aldrich, Strem, Acros, TCI or Alfa Aesar and used as such unless stated otherwise. Solvents (Anhydrous and under inert atmosphere) were collected from The Solventpurification system by M BRAUN and used under standard schlenk technique. NMR spectra were recorded on Bruker Avance 600 and Bruker ARX 400 spectrometers. Chemical shifts (ppm) are given relative to solvent: references for $CDCl_3$ were 7.26 ppm (^{1}H NMR) and 77.00 ppm (^{13}C NMR). Multiplets were assigned as s (singlet), d (doublet), t (triplet), q (quartet), p (pentet) dd (doublet of doublet), m (multiplet) and br. s (broad singlet). GC-yields were calculated using isooctane as internal standard. All measurements were carried out at room temperature unless otherwise stated. Electron impact (EI) mass spectrawere recorded on AMD 402 mass spectrometer (70 eV). High resolution mass spectra (HRMS) were recorded on Agilent 6210. The data are given as mass units per charge (m/z). Gas chromatography analysis was performed on an Agilent HP-7890A instrument with a FID detector and HP-5 capillary column (polydimethylsiloxane with 5% phenyl groups, 30 m, 0.32 mm i.d., 0.25 μm film thickness) using argon as carrier gas. The products were isolated from the reaction mixture by column chromatography on silica gel 60, 0.063-0.2 mm, 70-230 mesh (Merck).

A. General Procedure of Thiomethylation and Carbonylative Thiomethylation

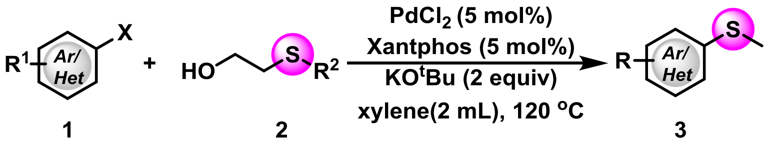

Under an N_2 atmosphere, a 25 mL pressure tube was charged with ArX (0.2 mmol), $PdCl_2$ (1.8 mg, 5 mol%), Xantphos (5.8 mg, 5 mol%), KO^{t}Bu (44.8mg, 2 equiv) and an oven-dried stirring bar. Then **2** (0.6 mmol) and xylene (2 mL) were injected by syringe. The pressure tube was sealed and the reaction was allowed to be heated under 120 °C for 12 hours. Afterwards, the reaction was cooled to room temperature. After removal of solvent under reduced pressure, pure product was obtained by column chromatography on silica gel (eluent: pentane/ethyl acetate = 100:1).

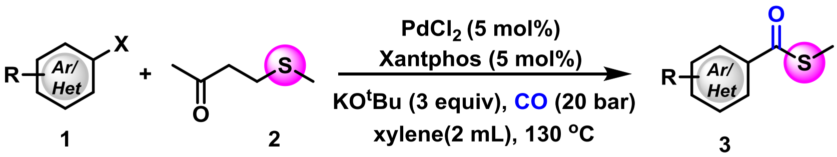

Under an N_2 atmosphere, a 4 mL screwcap vial was charged with ArX (0.2 mmol), $PdCl_2$ (1.8 mg, 5 mol%), Xantphos (5.8 mg, 5 mol%), KO^{t}Bu (67.2 mg, 3 equiv) and an oven-dried stirring bar. Then 4-(methylthio)butan-2-one (70.8 mg, 0.6 mmol) and xylene (2 mL) were injected by syringe. The vial was closed by a Teflon septum and a phenolic cap and connected to the atmosphere through a needle. Then the vial was fixed in an alloy plate and put into Parr 4560 series autoclave (300 mL). At room temperature, the autoclave was flushed with carbon monoxide for three times and 20 bar of carbon monoxide was charged. The autoclave was placed on a heating plate equipped with magnetic stirring and an aluminum block. The reaction was heated at 130 °C for 16 hours. Afterwards, the autoclave was cooled to room temperature and the pressure was carefully released. After removal of solvent under reduced pressure, pure product was obtained by column chromatography on silica gel (eluent: eluent: pentane/ethyl acetate = 100:1).

General Procedure of Thiomethylation and Carbonylative Thiomethylation

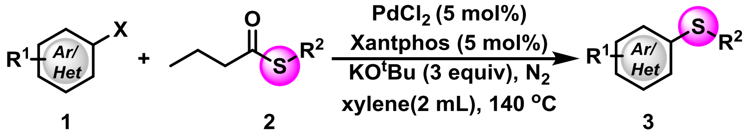

A 25 mL pressure tube was charged with ArX (0.2 mmol), $PdCl_2$ (1.8 mg, 5 mol%), Xantphos (5.8 mg, 5 mol%), KO^tBu (67.2 mg, 3 equiv) and an oven-dried stirring bar. After exchange N_2, **2** (0.6 mmol) and xylene (2 mL) were injected by syringe. The reaction was allowed to be heated under 140 °C for 12 hours. Afterwards, the reaction was cooled to room temperature. After removal of solvent under reduced pressure, pure product was obtained by column chromatography on silica gel (eluent: pentane/ethyl acetate = 100:1).

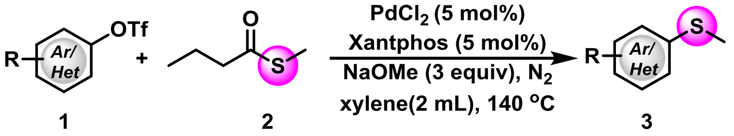

A 25 mL tube was charged with ArX (0.2 mmol), $PdCl_2$ (1.8 mg, 5 mol%), Xantphos (5.8 mg, 5 mol%), NaOMe (32.4mg, 3 equiv) and an oven-dried stirring bar. After exchange $N_{2,}$ *S*-methyl butanethioate (70.8 mg, 0.6 mmol) and xylene (2 mL) were injected by syringe. The reaction was allowed to be heated under 140 °C for 12 hours. Afterwards, the reaction was cooled to room temperature. After removal of solvent under reduced pressure, pure product was obtained by column chromatography on silica gel (eluent: pentane/ethyl acetate = 100:1).

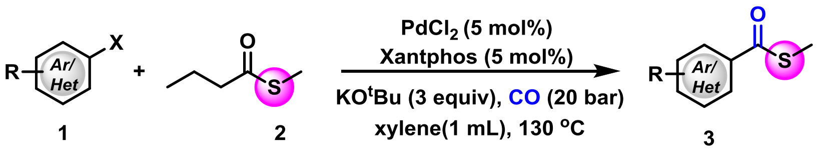

Under an N_2 atmosphere, a 4 mL screwcap vial was charged with ArX (0.2 mmol), $PdCl_2$ (1.8 mg, 5 mol%), Xantphos (5.8 mg, 5 mol%), KO^tBu (67.2 mg, 3 equiv) and an oven-dried stirring bar. Then *S*-methyl butanethioate (70.8 mg, 0.6 mmol) and xylene (1 mL) were injected by syringe. The vial was closed by a Teflon septum and a phenolic cap and connected to the atmosphere through a needle. Then the vial was fixed in an alloy plate and put into Parr 4560 series autoclave (300 mL). At room temperature, the autoclave was flushed with carbon monoxide for three times and 20 bar of carbon monoxide was charged. The autoclave was placed on a heating plate equipped with magnetic stirring and an aluminum block. The reaction was heated at 130 °C for 16 hours. Afterwards, the autoclave was cooled to room temperature and the pressure was carefully released. After removal of solvent under reduced pressure, pure product was obtained by column chromatography on silica gel (eluent: eluent: pentane/ethyl acetate = 100:1).

Pd-Catalyzed Thiomethylation of Aryl Iodide with different Alkyl sulfides

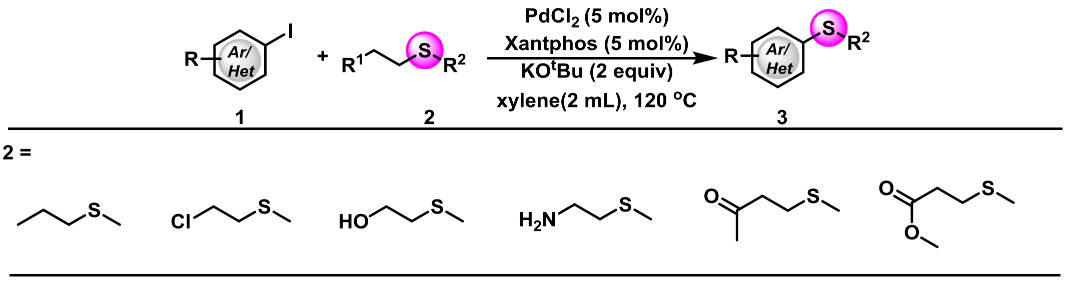

A 25 mL pressure tube was charged with ArI (0.2 mmol), $PdCl_2$ (5 mol%), Xantphos (5 mol%), KO^tBu (2 equiv) and an oven-dried stirring bar. Then, **2** (0.6 mmol) and xylene (2 mL) were injected by syringe. The reaction was

allowed to be heated under 120 °C for 12 hours. Afterwards, the reaction was cooled to room temperature. Yields were determined by GC using n-dodecane as internal standard. Or after removal of solvent under reduced pressure, pure product was obtained by column chromatography on silica gel (eluent: pentane/ethyl acetate = 100:1).

Pd-Catalyzed Thiomethylation of Aryl halide with Methionine and Selenomethionine

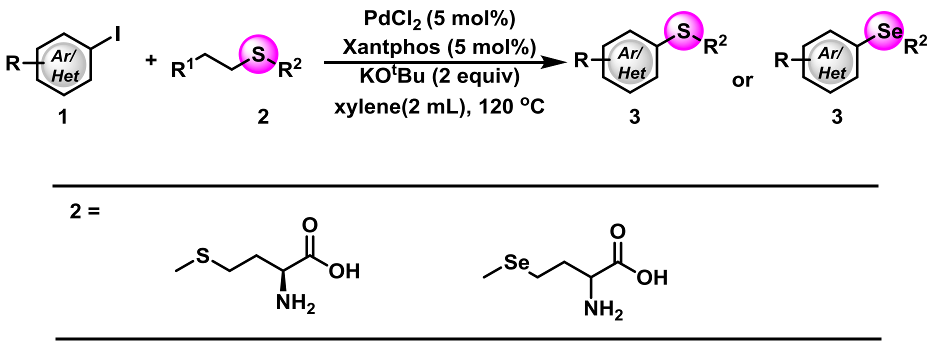

Methionine: A 25 mL pressure tube was charged with $PdCl_2$ (1.8 mg, 5 mol%), Xantphos (5.8mg, 5 mol%), KO^{t}Bu (67 mg, 3 equiv) and an oven-dried stirring bar. After exchange N_2, ArX (0.2 mmol), Methionine (0.6 mmol) and xylene (2 mL) were injected by syringe. The reaction was allowed to be heated under 130 °C for 24 hours. Afterwards, the reaction was cooled to room temperature. After removal of solvent under reduced pressure, pure product was obtained by column chromatography on silica gel (eluent: pentane/ethyl acetate = 100:1).

Selenomethionine: A 25 mL pressure tube was charged with $PdCl_2$ (1.8 mg, 5 mol%), Xantphos (5.8mg, 5 mol%), KO^{t}Bu (67 mg, 3 equiv) and an oven-dried stirring bar. After exchange N_2, ArX (0.2 mmol), Selenomethionine (0.6 mmol) and xylene (2 mL) were injected by syringe. The reaction was allowed to be heated under 140 °C for 24 hours. Afterwards, the reaction was cooled to room temperature. After removal of solvent under reduced pressure, pure product was obtained by column chromatography on silica gel (eluent: pentane/ethyl acetate = 100:1).

Control experiment

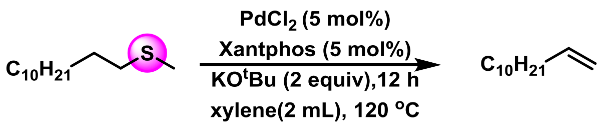

A 25 mL pressure tube was charged with $PdCl_2$ (1.8 mg, 5 mol%), Xantphos (5.8mg, 5 mol%), KO^{t}Bu (45 mg, 2 equiv) and an oven-dried stirring bar. After exchange N_2, dodecyl methyl sulfide (43.2 mg, 0.2 mmol) and xylene (2 mL) were injected by syringe. The reaction was allowed to be heated under 120 °C for 12 hours. Afterwards, the reaction was cooled to room temperature. Yield was determined by GC using n-dodecane as internal standard.

Gram-scale experiment

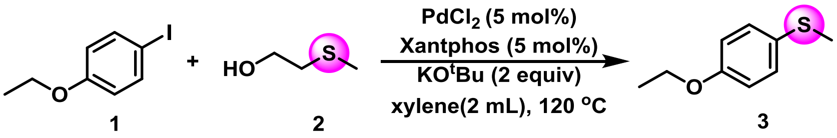

A 25 mL pressure tube was charged with ArI (0.2 mmol), $PdCl_2$ (1.8 mg, 5 mol%), Xantphos (5.8 mg, 5 mol%), KO^tBu (44.8mg, 2 equiv) and an oven-dried stirring bar. After exchange N_2, 2-mercaptoethanol (55.2 mg, 0.6 mmol) and xylene (2 mL) were injected by syringe. The reaction was allowed to be heated under 120 ^oC for 12 hours. Afterwards, the reaction was cooled to room temperature. After removal of solvent under reduced pressure, pure product was obtained by column chromatography on silica gel (eluent: pentane/ethyl acetate = 100:1).

Analytic Data of Products

(3,5-Dimethylphenyl)(methyl)sulfane[1]

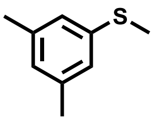

^{1}H NMR (600 MHz, Chloroform-*d*) δ 6.89 (s, 3H), 6.78 (s, 1H), 2.47 (s, 3H), 2.29 (s, 6H).
^{13}C NMR (151 MHz, $CDCl_3$) δ 138.40, 137.90, 126.97, 124.37, 21.23, 21.21, 15.86, 15.84.
GC-MS (EI, 70ev): m/z(%) = 152(M+, 100), 119(60), 105(30), 91(45), 77(20).

Methyl(naphthalen-1-yl)sulfane[1]

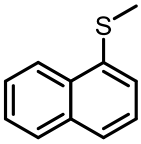

^{1}H NMR (600 MHz, Chloroform-*d*) δ 8.33 – 8.28 (m, 1H), 7.85 (dd, *J* = 8.0, 1.5 Hz, 1H), 7.69 (dt, *J* = 8.0, 1.1 Hz, 1H), 7.54 (dddd, *J* = 20.5, 8.1, 6.8, 1.4 Hz, 2H), 7.47 – 7.38 (m, 2H), 2.59 (s, 3H).
^{13}C NMR (151 MHz, $CDCl_3$) δ 135.81, 133.65, 131.74, 128.50, 126.19, 126.10, 125.88, 125.64, 124.32, 123.86, 16.29.
GC-MS (EI, 70ev): m/z(%) = 174(M+, 100), 159(50), 141(18), 128(10), 115(80), 87(10), 77(7).

(3,5-Dimethoxyphenyl)(methyl)sulfane[2]

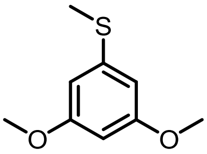

^{1}H NMR (600 MHz, Chloroform-*d*) δ 6.41 (d, *J* = 2.2 Hz, 2H), 6.24 (t, *J* = 2.2 Hz, 2H), 3.78 (s, 6H), 2.47 (s, 3H).
^{13}C NMR (151 MHz, $CDCl_3$) δ 160.95, 140.59, 104.46, 97.42, 55.33, 55.31, 15.60.
GC-MS (EI, 70ev): m/z(%) = 184(M+, 100), 151(78), 141(10), 121(15), 108(20), 91(10).

(4-Ethoxyphenyl)(methyl)sulfane[3]

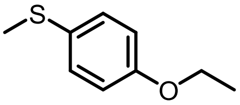

^{1}H NMR (600 MHz, Chloroform-*d*) δ 7.29 – 7.24 (m, 2H), 6.87 – 6.81 (m, 2H), 4.01 (qd, *J* = 6.9, 1.5 Hz, 2H), 2.44 (d, *J* = 1.6 Hz, 3H), 1.40 (td, *J* = 7.0, 1.6 Hz, 3H).
^{13}C NMR (151 MHz, $CDCl_3$) δ 157.56, 130.23, 128.57, 115.20, 115.18, 63.57, 18.09, 14.78.
GC-MS (EI, 70ev): m/z(%) = 168(M+, 100), 140(78), 139(30), 125(95), 111(12), 97(10), 81(8), 77(10), 65(10).

1,4-Bis(methylthio)benzene[1]

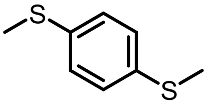

^{1}H NMR (600 MHz, Chloroform-*d*) δ 7.20 (s, 3H), 2.46 (s, 3H).
^{13}C NMR (151 MHz, $CDCl_3$) δ 135.20, 127.68, 127.66, 16.40.
GC-MS (EI, 70ev): m/z(%) = 170(M+, 100), 155(82), 108(10).

5-(Methylthio)benzo[d][1,3]dioxole[4]

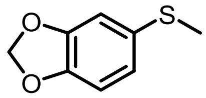

^{1}H NMR (600 MHz, Chloroform-*d*) δ 7.18 (d, *J* = 1.9 Hz, 1H), 7.14 (dd, *J* = 8.1, 1.9 Hz, 1H), 7.08 (d, *J* = 8.1 Hz, 1H), 6.28 (s, 2H), 2.78 (s, 3H).
^{13}C NMR (151 MHz, $CDCl_3$) δ 148.04, 146.22, 130.50, 121.92, 121.89, 109.41, 109.39, 108.70, 101.13, 18.01.
GC-MS (EI, 70ev): m/z(%) = 168(M+, 100), 153(72), 123(18), 95(30).

Methyl(4-phenoxyphenyl)sulfane[5]

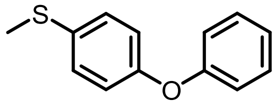

^{1}H NMR (600 MHz, Chloroform-*d*) δ 7.35 – 7.31 (m, 2H), 7.29 – 7.26 (m, 2H), 7.10 (tt, *J* = 7.5, 1.1 Hz, 1H), 7.01 – 6.98 (m, 2H), 6.97 – 6.93 (m, 2H), 2.48 (s, 3H).
^{13}C NMR (151 MHz, $CDCl_3$) δ 157.25, 155.33, 132.27, 129.71, 129.28, 123.22, 119.57, 118.66, 17.19.
GC-MS (EI, 70ev): m/z(%) = 216(M+, 100), 201(42), 129(23), 77(25), 51(15).

(4-(Benzyloxy)phenyl)(methyl)sulfane[4]

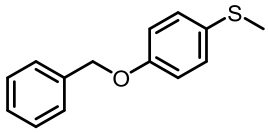

^{1}H NMR (600 MHz, Chloroform-*d*) δ 7.42 (d, *J* = 7.4 Hz, 2H), 7.40 – 7.36 (m, 2H), 7.33 (t, *J* = 7.2 Hz, 1H), 7.27 (d, *J* = 8.5 Hz, 2H), 6.95 – 6.90 (m, 2H), 5.05 (s, 2H), 2.45 (d, *J* = 1.0 Hz, 3H).
^{13}C NMR (151 MHz, $CDCl_3$) δ 157.15, 136.86, 133.15, 130.05, 128.57, 127.97, 127.41, 115.59, 70.16, 17.93.
GC-MS (EI, 70ev): m/z(%) = 230(M+, 35), 139(17), 91(100), 65(12).

(4-Chloro-3-(4-ethoxybenzyl)phenyl)(methyl)sulfane

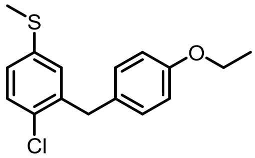

^{1}H NMR (600 MHz, Chloroform-*d*) δ 7.27 (t, *J* = 6.5 Hz, 1H), 7.09 (d, *J* = 8.5 Hz, 2H), 7.06 – 7.01 (m, 2H), 6.83 (d, *J* = 8.6 Hz, 2H), 4.01 (dd, *J* = 12.6, 5.6 Hz, 5H), 2.41 (s, 3H), 1.41 (t, *J* = 7.0 Hz, 3H).
^{13}C NMR (151 MHz, $CDCl_3$) δ 157.49, 139.54, 137.12, 130.97, 129.86, 129.73, 129.01, 125.66, 114.52, 63.40, 38.32, 16.03, 14.86.
GC-MS (EI, 70ev): m/z(%) = 292(M^+, 100), 264(18), 257(20), 229(33), 217(17), 182(28), 165(10), 152(20), 115(12), 107(28), 77(10).
HRMS(EI): calcd. for [$C_{16}H_{17}ClOS$]: 262.0662 found: 262.0658.

(4-(*tert*-Butyl)phenyl)(methyl)sulfane[4]

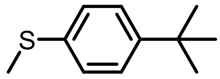

^{1}H NMR (600 MHz, Chloroform-*d*) δ 7.34 – 7.30 (m, 2H), 7.23 (d, *J* = 8.5 Hz, 2H), 2.48 (s, 3H), 1.31 (s, 9H).
^{13}C NMR (151 MHz, $CDCl_3$) δ 148.35, 134.78, 126.88, 125.84, 34.35, 31.29, 16.30, 16.26.
GC-MS (EI, 70ev): m/z(%) = 180(M+, 50), 165(100), 150(10), 137(20), 117(30), 115(13), 91(10), 77(5).

[1,1'-Biphenyl]-4-yl(methyl)sulfane[1]

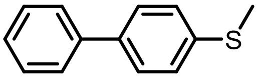

^{1}H NMR (600 MHz, Chloroform-*d*) δ 7.59 – 7.56 (m, 2H), 7.53 (d, *J* = 8.4 Hz, 2H), 7.44 (t, *J* = 7.7 Hz, 2H), 7.37 – 7.31 (m, 3H), 2.53 (s, 3H).
^{13}C NMR (151 MHz, $CDCl_3$) δ 140.53, 138.06, 137.57, 128.78, 127.46, 127.18, 127.00, 126.82, 15.93.
GC-MS (EI, 70ev): m/z(%) = 200(M+, 100), 185(50), 152(25), 115(12), 100(10).

(3-Chlorophenyl)(methyl)sulfane[6]

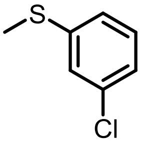

^{1}H NMR (600 MHz, Chloroform-*d*) δ 7.20 (dd, *J* = 13.8, 4.9 Hz, 2H), 7.11 (dd, *J* = 10.5, 8.5 Hz, 2H), 2.48 (s, 3H).
^{13}C NMR (151 MHz, $CDCl_3$) δ 140.68, 134.74, 129.73, 125.92, 125.01, 124.51, 15.58.
GC-MS (EI, 70ev): m/z(%) = 158(M+, 100), 143(18), 125(73), 112(27), 108(42), 75(13).

***N,N*-Dimethyl-4-(methylthio)aniline[4]**

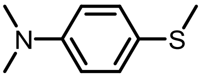

^{1}H NMR (600 MHz, Chloroform-*d*) δ 7.31 – 7.26 (m, 2H), 6.68 (dd, *J* = 9.4, 2.5 Hz, 2H), 2.94 (s, 6H), 2.42 (d, *J* = 4.1 Hz, 3H).
^{13}C NMR (151 MHz, $CDCl_3$) δ 149.47, 131.35, 123.45, 113.18, 40.56, 19.16.
GC-MS (EI, 70ev): m/z(%) = 167(M^+, 75), 152(100), 136(5), 108(12), 83(11), 77(10).

(4-Methoxyphenyl)(methyl)sulfane[1]

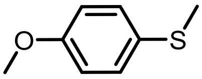

^{1}H NMR (600 MHz, Chloroform-*d*) δ 7.28 (d, *J* = 8.6 Hz, 2H), 6.84 (t, *J* = 9.0 Hz, 2H), 3.81 (d, *J* = 17.6 Hz, 3H), 2.44 (s, 3H).
^{13}C NMR (151 MHz, $CDCl_3$) δ 158.19, 130.20, 128.76, 114.59, 55.37, 55.32, 18.09, 18.07.
GC-MS (EI, 70ev): m/z(%) = 154(M+, 90), 139(100), 124(12), 111(15), 95(10), 77(10).

6-(Methylthio)quinoline[4]

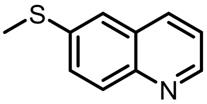

^{1}H NMR (600 MHz, Chloroform-*d*) δ 8.80 (dd, *J* = 4.0, 1.3 Hz, 1H), 8.01 (d, *J* = 8.2 Hz, 1H), 7.97 (d, *J* = 8.9 Hz, 1H), 7.57 (dd, *J* = 8.8, 2.0 Hz, 1H), 7.50 (d, *J* = 1.8 Hz, 1H), 7.35 (dd, *J* = 8.3, 4.2 Hz, 1H), 2.57 (s, 3H).
^{13}C NMR (151 MHz, $CDCl_3$) δ 149.45, 146.48, 137.48, 134.69, 129.58, 128.99, 128.75, 122.51, 121.60, 15.64.
GC-MS (EI, 70ev): m/z(%) = 175(M+ ,100), 165(50), 142(22) ,129(25), 116(30), 102(10), 89(17), 77(11).

Naphthalen-1-yl(phenyl)sulfane[7]

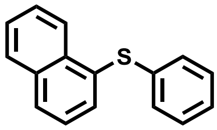

^{1}H NMR (600 MHz, Chloroform-*d*) δ 8.40 (dt, *J* = 6.3, 3.5 Hz, 1H), 7.92 – 7.84 (m, 2H), 7.68 (d, *J* = 7.2 Hz, 1H), 7.53 (dd, *J* = 6.4, 3.3 Hz, 2H), 7.44 (t, *J* = 7.7 Hz, 1H), 7.28 – 7.13 (m, 5H).
^{13}C NMR (151 MHz, $CDCl_3$) δ 136.91, 134.24, 133.60, 132.51, 131.28, 129.16, 129.04, 129.01, 128.53, 126.90, 126.39, 126.12, 125.79, 125.62.
GC-MS (EI, 70ev): m/z(%) = 236(M+ ,100), 221(10), 202(25) ,127(15), 115(30), 77(20), 51(18).

***N*-Methyl-4-(methylthio)benzamide**[8]

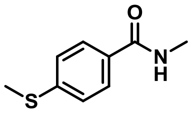

^{1}H NMR (600 MHz, Chloroform-*d*) δ 7.70 – 7.62 (m, 2H), 7.23 (d, *J* = 8.4 Hz, 2H), 6.29 (s, 1H), 2.98 (d, *J* = 4.8 Hz, 3H), 2.49 (s, 3H).
^{13}C NMR (151 MHz, $CDCl_3$) δ 167.65, 143.15, 130.83, 127.21, 125.51, 26.74, 15.08, 15.06.
GC-MS (EI, 70ev): m/z(%) = 181(M+ ,80), 151(100), 123(25) ,108(12), 77(20).

7-(Methylthio)-1*H*-indole

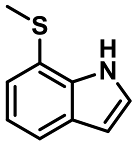

^{1}H NMR (600 MHz, Chloroform-*d*) δ 8.50 (s, 1H), 7.58 (d, *J* = 7.9 Hz, 1H), 7.32 – 7.21 (m, 2H), 7.13 (t, *J* = 7.7 Hz, 1H), 6.61 (s, 1H), 2.52 (s, 3H).
^{13}C NMR (151 MHz, $CDCl_3$) δ 136.16, 127.70, 124.21, 120.47, 119.89, 118.82, 103.37, 103.35, 18.05.
HR-MS (ESI-TOF) calcd. for C_9H_9NS [M+H]+: 164.0456; found: 264.10466.

1-(4-(Methylthio)phenyl)ethan-1-one[9]

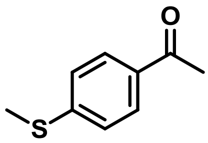

^{1}H NMR (600 MHz, Chloroform-*d*) δ 7.91 – 7.80 (m, 2H), 7.31 – 7.18 (m, 2H), 2.56 (s, 3H), 2.51 (s, 3H).
^{13}C NMR (151 MHz, $CDCl_3$) δ 197.01, 145.82, 133.57, 128.68, 125.02, 26.33, 26.31, 14.78, 14.76.
GC-MS (EI, 70ev): m/z(%) = 166(M+ ,70), 151(100), 123(27) ,108(18), 79(15).

2-(Methylthio)benzo[d]thiazole[10]

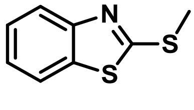

^{1}H NMR (600 MHz, Chloroform-*d*) δ 7.87 (d, *J* = 8.2 Hz, 1H), 7.75 (dd, *J* = 8.1, 1.3 Hz, 1H), 7.41 (ddd, *J* = 8.4, 7.2, 1.3 Hz, 1H), 7.28 (td, *J* = 7.6, 7.2, 1.2 Hz, 1H), 2.79 (s, 4H).

^{13}C NMR (151 MHz, $CDCl_3$) δ 167.97, 153.38, 135.17, 126.01, 124.06, 121.39, 120.91, 15.91.
GC-MS (EI, 70ev): m/z(%) = 181(M+ ,100), 148(75), 122(18) ,108(35), 69(18).

(1,2,3,6,7,8-Hexahydropyren-4-yl)(methyl)sulfane

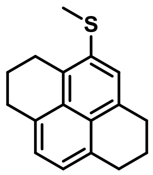

^1H NMR (600 MHz, Chloroform-*d*) δ 7.19 (s, 1H), 7.13 (d, *J* = 7.1 Hz, 1H), 7.09 (d, *J* = 7.1 Hz, 1H), 3.08 (ddt, *J* = 28.2, 15.9, 6.2 Hz, 8H), 2.55 (s, 2H), 2.07 (td, *J* = 6.3, 2.2 Hz, 4H).
^{13}C NMR (151 MHz, $CDCl_3$) δ 134.57, 134.12, 133.50, 131.73, 131.32, 130.32, 128.22, 124.13, 123.00, 122.39, 31.51, 31.31, 31.22, 28.44, 23.23, 22.84, 16.33.
GC-MS (EI, 70ev): m/z(%) = 254(M+ ,100), 239(40), 206(22) ,178(25), 89(15)
HRMS(EI): calcd. for [$C_{17}H_{18}S$]: 254.1123, found: 254.1119.

4-(Methylthio)benzonitrile[11]

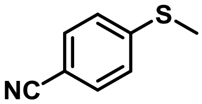

^1H NMR (600 MHz, Chloroform-*d*) δ 7.57 – 7.47 (m, 2H), 7.25 (d, *J* = 8.4 Hz, 2H), 2.50 (s, 3H).
^{13}C NMR (151 MHz, $CDCl_3$) δ 146.09, 132.12, 125.54, 125.52, 118.88, 107.72, 14.69.
GC-MS (EI, 70ev): m/z(%) = 149(M+ ,100), 134(28), 116(53) ,104(22), 90(13) , 75(12).

4,4,5,5-Tetramethyl-2-(4-(methylthio)phenyl)-1,3,2-dioxaborolane[12]

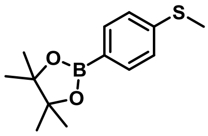

^1H NMR (600 MHz, Chloroform-*d*) δ 7.77 – 7.63 (m, 2H), 7.23 (d, *J* = 8.2 Hz, 2H), 2.48 (s, 3H), 1.34 (s, 12H).
^{13}C NMR (151 MHz, $CDCl_3$) δ 142.52, 135.05, 125.12, 83.69, 24.82, 15.11.
GC-MS (EI, 70ev): m/z(%) = 250(M+ ,100), 235(28), 164(42) ,150(80), 117(18) , 85(12).

Cyclohexyl(4-methoxyphenyl)sulfane[13]

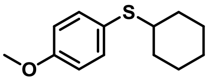

^1H NMR (600 MHz, Chloroform-*d*) δ 7.64 – 7.21 (m, 2H), 6.97 – 6.62 (m, 2H), 3.79 (s, 3H), 2.90 (ddd, *J* = 10.7, 6.9, 3.7 Hz, 1H), 1.92 (dd, *J* = 10.2, 5.2 Hz, 2H), 1.75 (dt, *J* = 12.5, 3.6 Hz, 2H), 1.60 (q, *J* = 7.1, 5.4 Hz, 1H), 1.42 – 1.12 (m, 5H).
^{13}C NMR (151 MHz, $cdcl_3$) δ 159.29, 135.55, 135.50, 125.04, 114.26, 55.30, 55.23, 47.89, 33.37, 26.09, 25.76.
GC-MS (EI, 70ev): m/z(%) = 222(M+ ,38), 140(100), 125(25) ,55(20).

Decyl(4-methoxyphenyl)sulfane

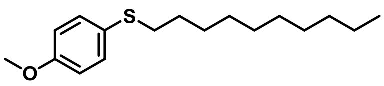

^{1}H NMR (600 MHz, Chloroform-*d*) δ 7.48 – 7.17 (m, 2H), 6.84 (d, *J* = 8.3 Hz, 2H), 3.79 (s, 3H), 2.81 (t, *J* = 7.4 Hz, 2H), 1.62 – 1.53 (m, 2H), 1.38 (t, *J* = 7.5 Hz, 2H), 1.26 (s, 11H), 0.88 (t, *J* = 7.0 Hz, 3H).
^{13}C NMR (151 MHz, $cdcl_3$) δ 158.70, 132.88, 132.85, 132.83, 127.04, 114.47, 55.31, 55.25, 35.82, 31.87, 29.52, 29.49, 29.36, 29.28, 29.16, 28.69, 22.65, 14.07.
GC-MS (EI, 70ev): m/z(%) = 280(M+ ,100), 153(10), 140(78) ,125(15) , 55(10).

S-(*p*-Tolyl) quinoline-6-carbothioate[14]

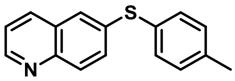

^{1}H NMR (600 MHz, Chloroform-*d*) δ 8.83 (dd, *J* = 4.2, 1.8 Hz, 1H), 8.03 – 7.91 (m, 2H), 7.59 (d, *J* = 2.1 Hz, 1H), 7.53 (dd, *J* = 8.9, 2.1 Hz, 1H), 7.38 (d, *J* = 7.8 Hz, 2H), 7.33 (dd, *J* = 8.3, 4.2 Hz, 1H), 7.18 (d, *J* = 7.8 Hz, 2H), 2.36 (s, 3H).
^{13}C NMR (151 MHz, $cdcl_3$) δ 150.00, 146.96, 138.36, 136.45, 135.10, 133.10, 130.61, 130.33, 130.05, 128.63, 126.51, 121.54, 21.20.

S-Methyl benzothioate[15]

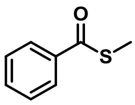

^{1}H NMR (600 MHz, Chloroform-*d*) δ 8.00 – 7.92 (m, 2H), 7.60 – 7.53 (m, 1H), 7.47 – 7.40 (m, 2H), 2.48 (s, 3H).
^{13}C NMR (151 MHz, $cdcl_3$) δ 192.42, 137.06, 133.22, 128.57, 127.10, 11.68.

S-Methyl naphthalene-2-carbothioate

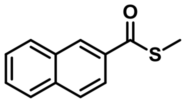

^{1}H NMR (600 MHz, Chloroform-*d*) δ 8.53 (d, *J* = 1.7 Hz, 1H), 8.00 (dd, *J* = 8.6, 1.8 Hz, 1H), 7.97 (d, *J* = 8.1 Hz, 1H), 7.88 (t, *J* = 8.8 Hz, 2H), 7.59 (ddd, *J* = 8.2, 6.8, 1.4 Hz, 1H), 7.55 (ddd, *J* = 8.1, 6.8, 1.3 Hz, 1H), 2.53 (s, 3H).
^{13}C NMR (151 MHz, $cdcl_3$) δ 192.29, 135.71, 134.38, 132.46, 129.52, 128.43, 128.41, 128.36, 127.77, 126.86, 123.11, 11.81.
GC-MS (EI, 70ev): m/z(%) = 202(M+ ,18), 155(100), 127(90) ,101(10) , 77(12).

S-Methyl 3,5-dimethoxybenzothioate

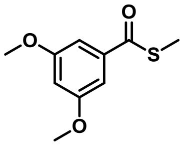

^{1}H NMR (600 MHz, Chloroform-*d*) δ 7.10 (d, *J* = 2.4 Hz, 2H), 6.65 (t, *J* = 2.3 Hz, 1H), 3.83 (s, 6H), 2.46 (s, 3H).
^{13}C NMR (151 MHz, $cdcl_3$) δ 192.26, 160.81, 139.02, 105.65, 104.88, 55.57, 55.56, 11.81.
GC-MS (EI, 70ev): m/z(%) = 212(M+ ,17), 137(28), 122(27) ,107(18), 77(15).

S-Methyl 4-methyl-3-(trifluoromethyl)benzothioate

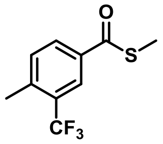

^{1}H NMR (600 MHz, Chloroform-*d*) δ 8.20 (d, *J* = 1.9 Hz, 1H), 8.01 (dd, *J* = 8.0, 1.8 Hz, 1H), 7.38 (d, *J* = 7.9 Hz, 1H), 7.26 (s, 1H), 2.54 (d, *J* = 1.9 Hz, 3H), 2.49 (s, 3H).
^{13}C NMR (151 MHz, Chloroform-*d*) δ 191.01, 142.37, 134.93, 132.28, 130.04, 129.41 (d, *J* = 30.3 Hz), 124.82, 124.64 (q, *J* = 5.6 Hz), 123.00, 19.54, 11.72.

S-Methyl 4-chloro-3-(4-ethoxybenzyl)benzothioate

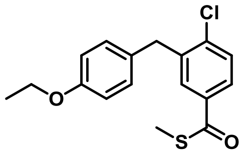

^{1}H NMR (600 MHz, Chloroform-*d*) δ 7.84 – 7.67 (m, 2H), 7.44 (d, *J* = 8.2 Hz, 1H), 7.10 (d, *J* = 8.4 Hz, 2H), 6.92 – 6.74 (m, 2H), 4.08 (s, 2H), 4.01 (qd, *J* = 6.9, 0.9 Hz, 2H), 2.45 (d, *J* = 1.1 Hz, 3H), 1.40 (td, *J* = 6.9, 0.9 Hz, 3H).
^{13}C NMR (151 MHz, $cdcl_3$) δ 191.32, 157.65, 139.83, 139.47, 135.72, 130.45, 129.82, 129.80, 129.38, 126.14, 114.68, 63.41, 38.35, 38.33, 38.31, 14.82, 11.69, 11.67.
GC-MS (EI, 70ev): m/z(%) = 320(M+ ,32), 273(100), 107(18), 135(14), 123(12), 152(10), 181(10).

S-Methyl 4-cyanobenzothioate

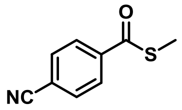

^{1}H NMR (600 MHz, Chloroform-*d*) δ 8.16 – 7.94 (m, 2H), 7.83 – 7.68 (m, 2H), 2.52 (d, *J* = 1.3 Hz, 3H).
^{13}C NMR (151 MHz, $cdcl_3$) δ 191.04, 140.09, 132.47, 127.55, 117.78, 116.56, 11.94.
GC-MS (EI, 70ev): m/z(%) =177(M+ ,10), 130(100), 102(40), 75(10).

S-Methyl 2-chloro-4-fluorobenzothioate

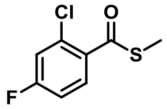

^{1}H NMR (600 MHz, Chloroform-*d*) δ 7.70 (dd, *J* = 8.7, 5.9 Hz, 1H), 7.19 (dd, *J* = 8.4, 2.5 Hz, 1H), 7.03 (ddd, *J* = 8.7, 7.7, 2.5 Hz, 1H), 2.49 (s, 3H).
^{13}C NMR (151 MHz, $cdcl_3$) δ 191.16, 164.62, 162.92, 133.78, 133.76, 132.68, 132.61, 131.08, 131.02, 118.51, 118.35, 114.10, 113.96, 12.64.
GC-MS (EI, 70ev): m/z(%) = 204(M+ ,10), 157(100), 129(34), 159(32).

S-Methyl 4-methylbenzothioate[16]

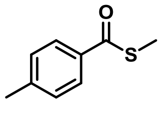

^{1}H NMR (600 MHz, Chloroform-*d*) δ 7.91 – 7.82 (m, 2H), 7.24 (d, *J* = 8.0 Hz, 2H), 2.46 (s, 3H), 2.40 (s, 3H).
^{13}C NMR (151 MHz, cdcl$_3$) δ 192.02, 144.05, 134.55, 129.22, 127.16, 21.62, 11.60, 11.58.
GC-MS (EI, 70ev): m/z(%) = 166(M+ ,10), 119(100), 91(50), 65(16).

S-Methyl 4-(dimethylamino)benzothioate[17]

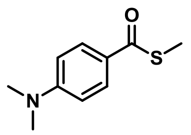

^{1}H NMR (600 MHz, Chloroform-*d*) δ 8.04 – 7.71 (m, 2H), 6.78 – 6.49 (m, 2H), 3.04 (s, 6H), 2.43 (s, 3H).
^{13}C NMR (151 MHz, cdcl$_3$) δ 190.17, 153.63, 129.15, 124.80, 110.60, 40.01, 39.99, 11.33.
GC-MS (EI, 70ev): m/z(%) = 195(M+ ,22), 148(100), 120(12) ,105(10), 77(12), 91(10).

S-Methyl 4-(methylcarbamoyl)benzothioate

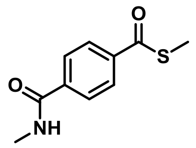

^{1}H NMR (600 MHz, Chloroform-*d*) δ 8.02 – 7.97 (m, 2H), 7.82 (d, *J* = 8.5 Hz, 2H), 3.02 (d, *J* = 4.7 Hz, 3H), 2.49 (s, 3H).
^{13}C NMR (151 MHz, cdcl$_3$) δ 191.76, 167.11, 139.11, 138.69, 127.32, 127.15, 26.93, 11.82.
GC-MS (EI, 70ev): m/z(%) = 209(M+ ,10), 162(100), 134(18), 103(25), 76(20).

S-Methyl 3-fluoro-4-methoxybenzothioate

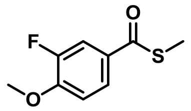

^{1}H NMR (600 MHz, Chloroform-*d*) δ 7.77 (d, *J* = 8.5 Hz, 1H), 7.69 (d, *J* = 11.7 Hz, 1H), 7.02 – 6.94 (m, 1H), 3.94 (s, 3H), 2.45 (s, 3H).
^{13}C NMR (151 MHz, cdcl$_3$) δ 190.16, 152.68, 152.00, 151.92, 151.04, 124.23, 124.20, 114.95, 114.82, 112.42, 56.29, 11.67.
GC-MS (EI, 70ev): m/z(%) = 200(M+ ,18),153(100), 125(22), 110(10), 95(18), 82(12).

S-Methyl 4-(benzyloxy)benzothioate

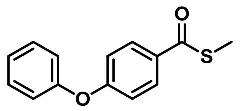

^{1}H NMR (600 MHz, Chloroform-*d*) δ 8.04 – 7.81 (m, 2H), 7.49 – 7.34 (m, 2H), 7.23 – 7.13 (m, 1H), 7.12 – 7.02 (m, 2H), 6.99 (d, *J* = 8.9 Hz, 2H), 2.46 (s, 3H).
^{13}C NMR (151 MHz, cdcl$_3$) δ 190.97, 162.13, 155.47, 131.63, 130.02, 129.26, 124.59, 120.12, 117.33, 11.64.
GC-MS (EI, 70ev): m/z(%) = 244(M+ ,10), 197(100), 141(18), 115(18), 77(10).

S-Methyl [1,1'-biphenyl]-4-carbothioate

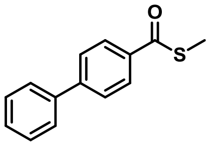

^{1}H NMR (600 MHz, Chloroform-*d*) δ 8.05 (d, *J* = 8.5 Hz, 2H), 7.70 – 7.65 (m, 2H), 7.64 – 7.60 (m, 2H), 7.50 – 7.44 (m, 2H), 7.43 – 7.38 (m, 1H), 2.50 (s, 3H).
^{13}C NMR (151 MHz, $cdcl_3$) δ 191.91, 146.01, 139.80, 135.78, 128.94, 128.22, 127.66, 127.23, 127.22, 11.71.
GC-MS (EI, 70ev): m/z(%) = 228(M+ ,15), 182(12), 181(100), 153(25), 152(46), 76(10).

S-Methyl 4-(pyrrolidin-1-yl)benzothioate

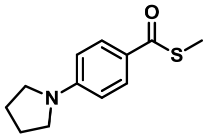

^{1}H NMR (600 MHz, Chloroform-*d*) δ 7.86 (d, *J* = 8.9 Hz, 2H), 6.48 (d, *J* = 8.9 Hz, 2H), 3.50 – 3.18 (m, 4H), 2.42 (s, 4H), 2.12 – 1.87 (m, 3H).
^{13}C NMR (151 MHz, $cdcl_3$) δ 190.03, 151.22, 129.31, 124.26, 110.63, 47.54, 25.41, 11.32.
GC-MS (EI, 70ev): m/z(%) = 221(M+ ,23), 174(100), 146(18), 117(10), 104(12), 77(10).

S-Methyl 4-hexylbenzothioate

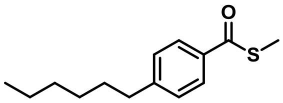

^{1}H NMR (600 MHz, Chloroform-*d*) δ 7.98 – 7.75 (m, 2H), 7.33 – 7.11 (m, 2H), 2.64 (t, *J* = 7.8 Hz, 2H), 2.46 (s, 3H), 1.70 – 1.49 (m, 2H), 1.43 – 1.15 (m, 6H), 1.02 – 0.79 (m, 3H).
^{13}C NMR (151 MHz, $cdcl_3$) δ 192.04, 149.02, 134.72, 128.58, 127.18, 35.99, 31.63, 31.04, 31.02, 28.88, 22.54, 14.04, 14.03, 11.59.

S-Methyl 4-methoxybenzothioate[18]

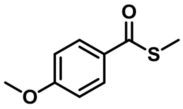

^{1}H NMR (600 MHz, Chloroform-*d*) δ 8.00 – 7.87 (m, 2H), 6.92 (d, *J* = 8.9 Hz, 2H), 3.86 (s, 3H), 2.45 (s, 3H).
^{13}C NMR (151 MHz, $cdcl_3$) δ 190.93, 163.65, 129.98, 129.24, 113.72, 55.47, 55.45, 11.56.
GC-MS (EI, 70ev): m/z(%) = 182(M+ ,10), 135(100), 107(18), 92(17), 77(30).

S-Methyl 4-morpholinobenzothioate

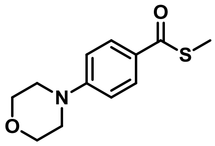

^{1}H NMR (600 MHz, Chloroform-*d*) δ 7.89 (d, *J* = 9.0 Hz, 2H), 6.84 (d, *J* = 9.1 Hz, 2H), 3.84 (dd, *J* = 5.8, 4.1 Hz, 4H), 3.29 (dd, *J* = 5.8, 4.0 Hz, 4H), 2.44 (s, 3H).

^{13}C NMR (151 MHz, $cdcl_3$) δ 190.48, 154.45, 129.00, 127.70, 113.31, 66.53, 47.54, 11.44.
GC-MS (EI, 70ev): m/z(%) = 237(M+ ,12), 190(100), 132(10), 91(12), 77(11).

S-Methyl 4-(*tert*-butyl)benzothioate

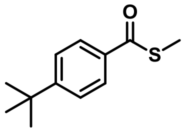

^{1}H NMR (600 MHz, Chloroform-*d*) δ 8.04 – 7.76 (m, 2H), 7.55 – 7.33 (m, 2H), 2.46 (s, 3H), 1.34 (s, 9H).
^{13}C NMR (151 MHz, $cdcl_3$) δ 192.01, 157.01, 134.47, 127.00, 125.50, 35.11, 31.06, 11.58.
GC-MS (EI, 70ev): m/z(%) =208(M+ ,10), 161(100), 146(18), 118(17), 91(12).

S-Methyl benzofuran-5-carbothioate

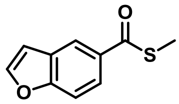

^{1}H NMR (600 MHz, Chloroform-*d*) δ 8.28 (t, *J* = 2.0 Hz, 1H), 7.96 (dt, *J* = 8.7, 2.0 Hz, 1H), 7.69 (s, 1H), 7.59 – 7.50 (m, 1H), 6.86 (td, *J* = 2.5, 1.1 Hz, 1H), 2.67 – 2.41 (m, 3H).
^{13}C NMR (151 MHz, $cdcl_3$) δ 191.95, 157.60, 146.43, 132.57, 127.52, 123.79, 121.19, 111.46, 107.23, 11.83.
GC-MS (EI, 70ev): m/z(%) =192(M+ ,14), 145(100),117(48), 89(25), 63(14).

S-Methyl quinoline-6-carbothioate

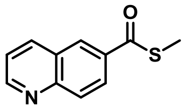

^{1}H NMR (600 MHz, Chloroform-*d*) δ 9.00 (dd, *J* = 4.2, 1.7 Hz, 1H), 8.49 (d, *J* = 2.0 Hz, 1H), 8.32 – 8.26 (m, 1H), 8.23 (dd, *J* = 8.8, 2.0 Hz, 1H), 8.15 (d, *J* = 8.8 Hz, 1H), 7.48 (dd, *J* = 8.3, 4.2 Hz, 1H), 2.54 (s, 3H).
^{13}C NMR (151 MHz, $cdcl_3$) δ 191.74, 152.54, 150.20, 137.46, 134.83, 130.13, 128.14, 127.44, 126.83, 122.01, 11.93.
GC-MS (EI, 70ev): m/z(%) = 203(M+ ,15), 156(100), 128(70), 101(25), 75(18).

S-Methyl 1*H*-indole-7-carbothioate

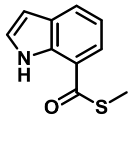

^{1}H NMR (600 MHz, Chloroform-*d*) δ 10.08 (s, 0H), 8.01 – 7.92 (m, 1H), 7.90 – 7.86 (m, 1H), 7.36 – 7.28 (m, 1H), 7.16 (t, *J* = 7.7 Hz, 1H), 6.59 (dd, *J* = 3.2, 2.2 Hz,1H), 2.52 (s, 3H).
^{13}C NMR (151 MHz, $cdcl_3$) δ 193.07, 133.46, 129.40, 126.88, 125.70, 122.91, 119.85, 119.04, 102.39, 11.28.
GC-MS (EI, 70ev): m/z(%) = 191(M+ ,44), 144(100), 116(76), 89(28).

S-Methyl 4-(benzyloxy)benzothioate

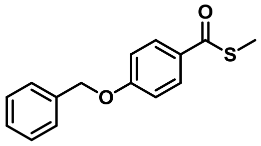

^{1}H NMR (600 MHz, Chloroform-*d*) δ 7.99 – 7.89 (m, 2H), 7.46 – 7.37 (m, 4H), 7.37 – 7.32 (m, 1H), 7.03 – 6.94 (m, 2H), 5.12 (s, 2H), 2.45 (s, 3H).
^{13}C NMR (151 MHz, $cdcl_3$) δ 190.88, 162.81, 136.14, 130.21, 129.26, 128.67, 128.22, 127.43, 114.61, 70.18, 11.57.
GC-MS (EI, 70ev): m/z(%) = 158(M+ ,10), 211(52), 91(100), 65(15).

S-Methyl benzo[d][1,3]dioxole-5-carbothioate[19]

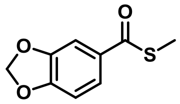

^{1}H NMR (600 MHz, Chloroform-*d*) δ 7.60 (dt, *J* = 8.2, 1.4 Hz, 1H), 7.48 – 7.30 (m, 1H), 6.93 – 6.78 (m, 1H), 6.04 (d, *J* = 1.1 Hz, 2H), 2.44 (d, *J* = 1.2 Hz, 3H).
^{13}C NMR (151 MHz, $cdcl_3$) δ 190.65, 151.88, 148.00, 131.65, 123.12, 107.98, 107.14, 101.88, 11.75.
GC-MS (EI, 70ev): m/z(%) = 196(M+ ,18), 149(100), 121(28), 91(10), 65(22).

(4-Methoxyphenyl)(methyl)selane[20]

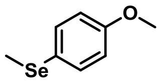

^{1}H NMR (600 MHz, Chloroform-*d*) δ 7.49 – 7.36 (m, 2H), 6.91 – 6.72 (m, 2H), 3.79 (s, 3H), 2.30 (s, 3H).
^{13}C NMR (151 MHz, $cdcl_3$) δ 158.83, 133.45, 121.53, 114.80, 55.30, 55.25, 8.67, 8.65.
GC-MS (EI, 70ev): m/z(%) =202(M+ ,100), 200(50), 189(22), 187(96), 185(46), 183(22), 172(18), 144(18), 121(15), 78(28), 69(19).

S-Methyl 4-(methylthio)benzothioate

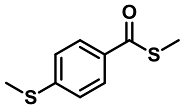

^{1}H NMR (600 MHz, Chloroform-*d*) δ 7.94 – 7.81 (m, 2H), 7.29 – 7.12 (m, 2H), 2.51 (s, 3H), 2.46 (s, 3H).
^{13}C NMR (151 MHz, $cdcl_3$) δ 191.33, 146.05, 133.34, 127.46, 127.44, 125.05, 125.00, 14.84, 14.79, 11.61, 11.56.
GC-MS (EI, 70ev): m/z(%) = 198(M+ ,18), 151(100), 123(20), 108(16), 79(10).

Reference

1. Luo, F.; Pan, C.; Li, L.; Chen, F.; Cheng, J. *Chem. Comm.* **2011,** *47*, 5304-5306.
2. Ghosh, K.; Ranjit, S.; Mal, D. *Tetrahedron Lett.* **2015,** *56*, 5199-5202.
3. Chianelli, D.; Testaferri, L.; Tiecco, M.; Tingoli, M. *Synthesis* **1982**, 475-478.
4. Joseph, P. J. A.; Priyadarshini, S.; Kantam, M. L.; Sreedhar, B. *Tetrahedron* **2013,** *69*, 8276-8283.
5. Zhai, Y.; Chen, X.; Zhou, W.; Fan, M.; Lai, Y.; Ma, D. *J. Org. Chem.* **2017,** *82*, 4964-4969.
6. Wu, X.; Wang, Y. *Tetrahedron Lett.* **2018,** *59*, 1240-1243.
7. Fernández-Salas, J. A.; Pulis, A. P.; Procter, D. J. *Chem. Comm.* **2016,** *52*, 12364-12367.
8. Paul, B.; Panja, D.; Kundu, S. *Org. Lett.* **2019,** *21*, 5843-5847.
9. Hooper, J. F.; Chaplin, A. B.; González-Rodríguez, C.; Thompson, A. L.; Weller, A. S.; Willis, M. C. *J. Am. Chem. Soc.* **2012,** *134*, 2906-2909
10. Dai, C.; Xu, Z.; Huang, F.; Yu, Z.; Gao, Y.-F. J. *Chem. Comm.* **2012,** *77*, 4414-4419.
11. Wang, M.; Qiao, Z.; Zhao, J.; Jiang, X. *Org. Lett.* **2018,** *20*, 6193-6197.
12. Chandrashekar, H. B.; Maji, A.; Halder, G.; Banerjee, S.; Bhattacharyya, S.; Maiti, D. *Chem. Commun.,* **2019***,55,* 6201-6204.
13. Xu, X.-B.; Liu, J.; Zhang, J.-J.; Wang, Y.-W.; Peng, Y. *Org. Lett.* **2013,** *15*, 550-553.
14. Lin, Y.-M.; Lu, G.-P.; Wang, G.-X.; Yi, W.-B. *Adv. Synth. Catal.***2016,** *358*, 4100-4105.
15. Arisawa, M.; Kuwajima, M.; Toriyama, F.; Li, G.; Yamaguchi, M. *Org. Lett.* **2012,** *14*, 3804-3807.
16. Kanda, T.; Ibi, M.; Mochizuki, K.-i.; Kato, S. *Chem. Lett.* **1998,** *27*, 957-958.
17. Boeini, H. Z.; Kashan, M. E. *Green Chem.* **2009,** *11*, 1987-1991.
18. Abbasi, M.; Khalifeh, R. *Beilstein J. Org. Chem.* **2015**, *11*, 1265-1273
19. Harrowven, D. C.; Lucas, M. C.; Howes, P. D. *Tetrahedron* **1999,** *55*, 1187-1196.
20. Taniguchi, N. *Tetrahedron* **2012,** *68*, 10510-10515.

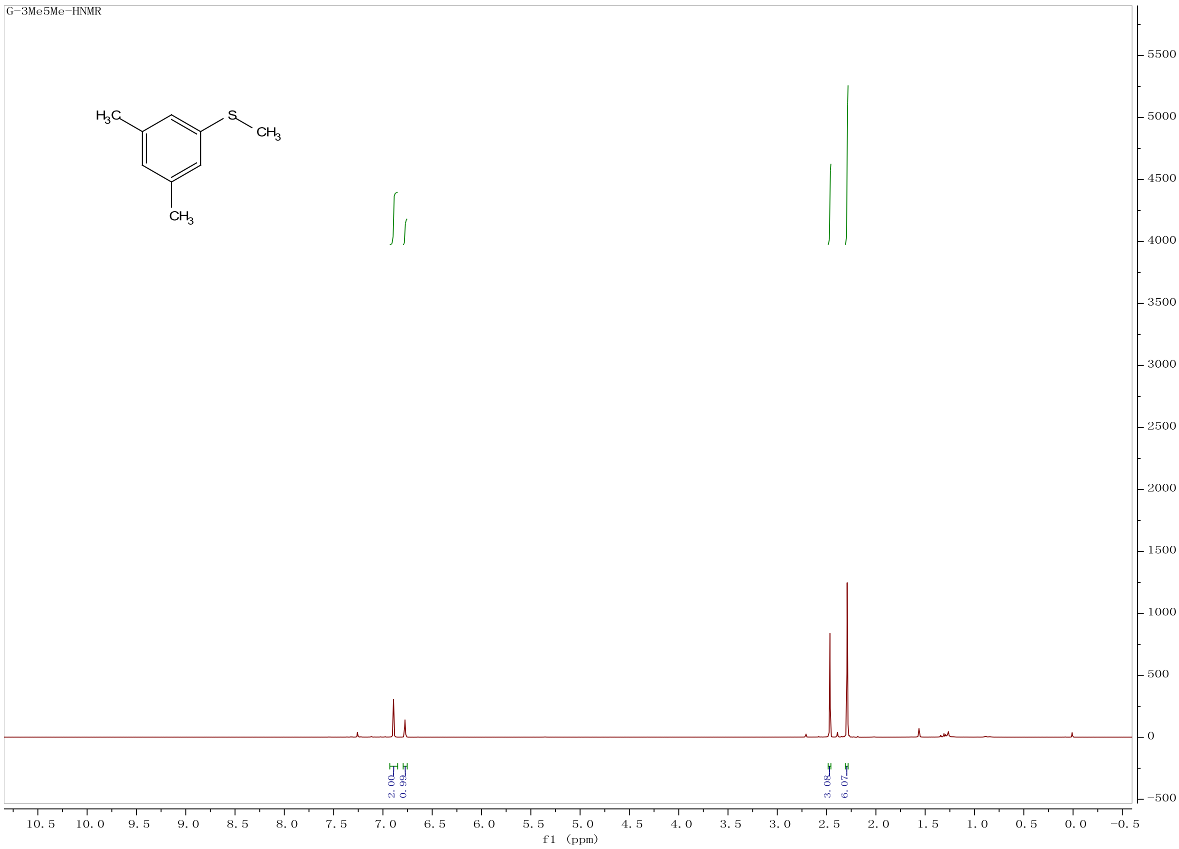
G-3Me5Me-HNMR
H3C
S
CH3
CH3
2.00
0.99
3.08
6.07
f1 (ppm)

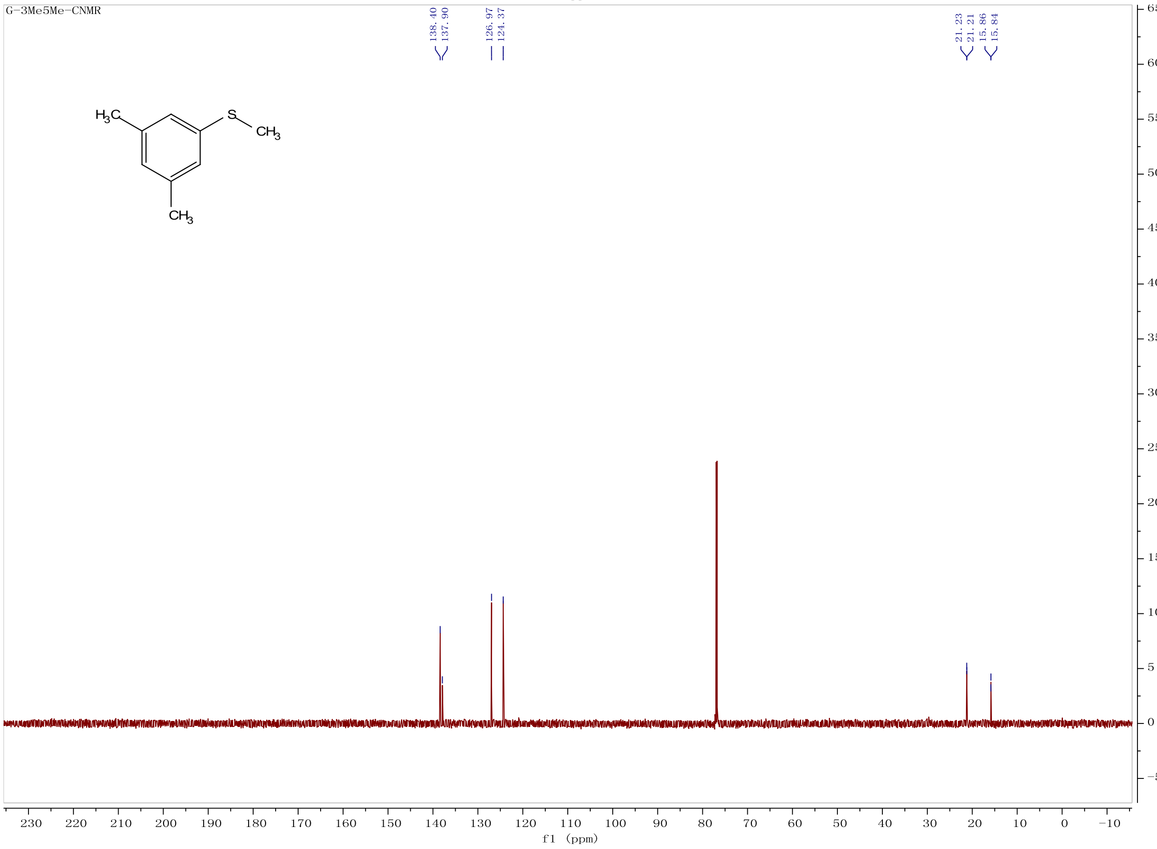
G-3Me5Me-CNMR
138.40
137.90
126.97
124.37
21.23
21.21
15.86
15.84
H3C
S
CH3
CH3
f1 (ppm)

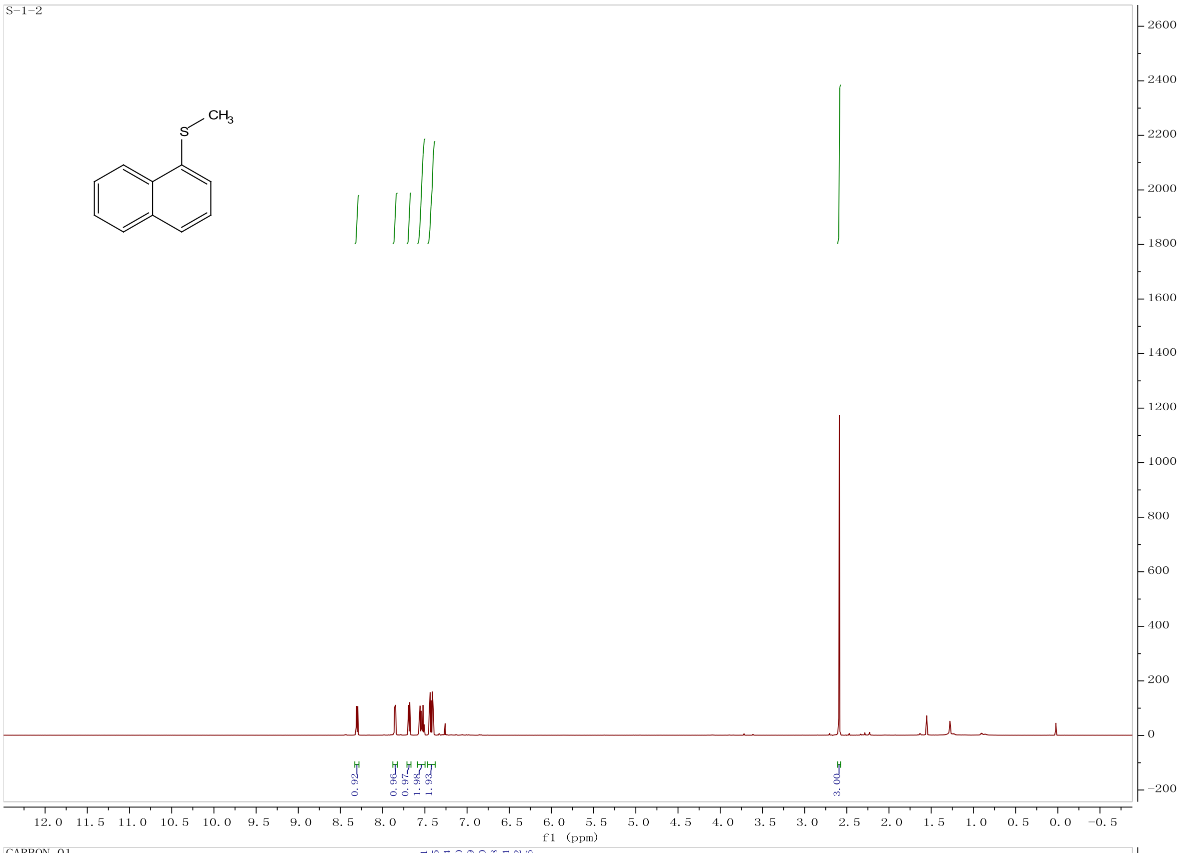
S-1-2
S
CH3
0.92
0.96
0.97
1.98
1.93
3.00
f1 (ppm)

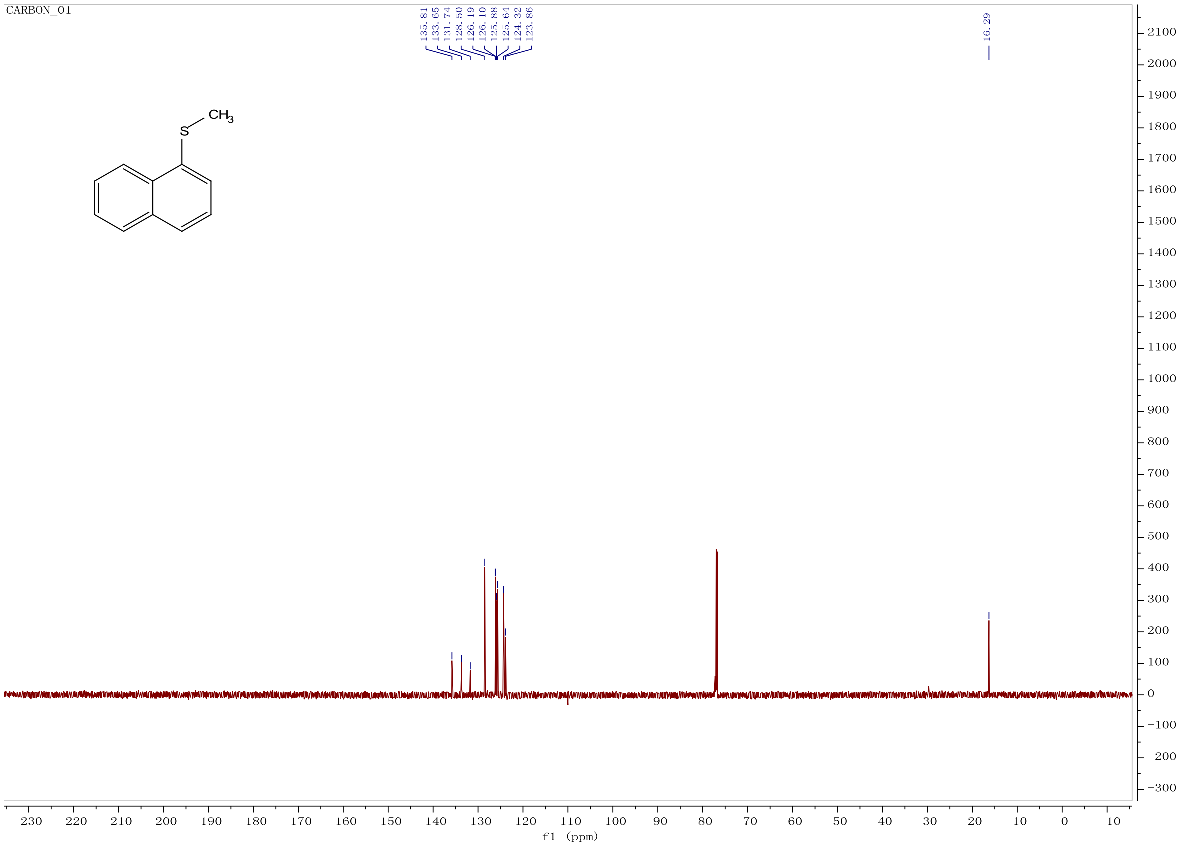
CARBON_01
135.81
133.65
131.74
128.50
126.19
126.10
125.88
125.64
124.32
123.86
16.29
S
CH3
f1 (ppm)

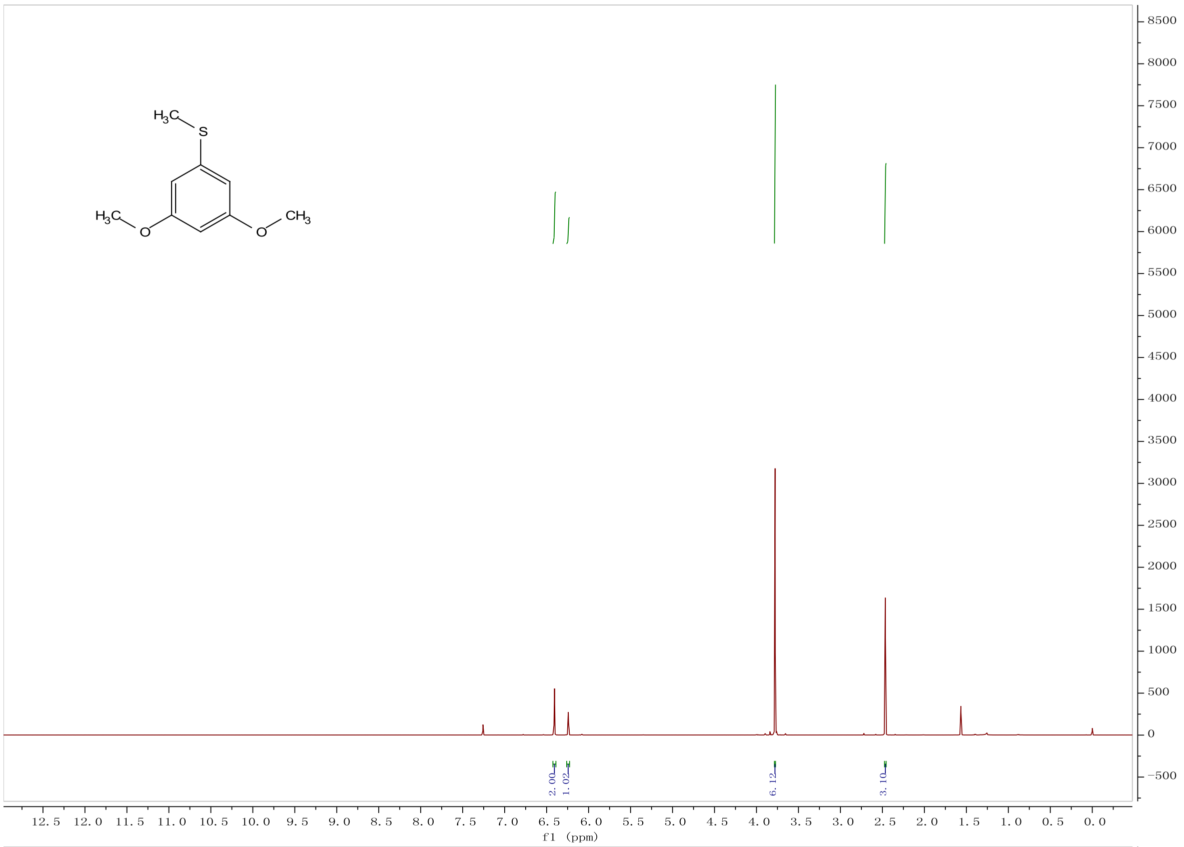

H_3C S
H_3C O
O CH_3
2.00
1.02
6.12
3.10
f1 (ppm)

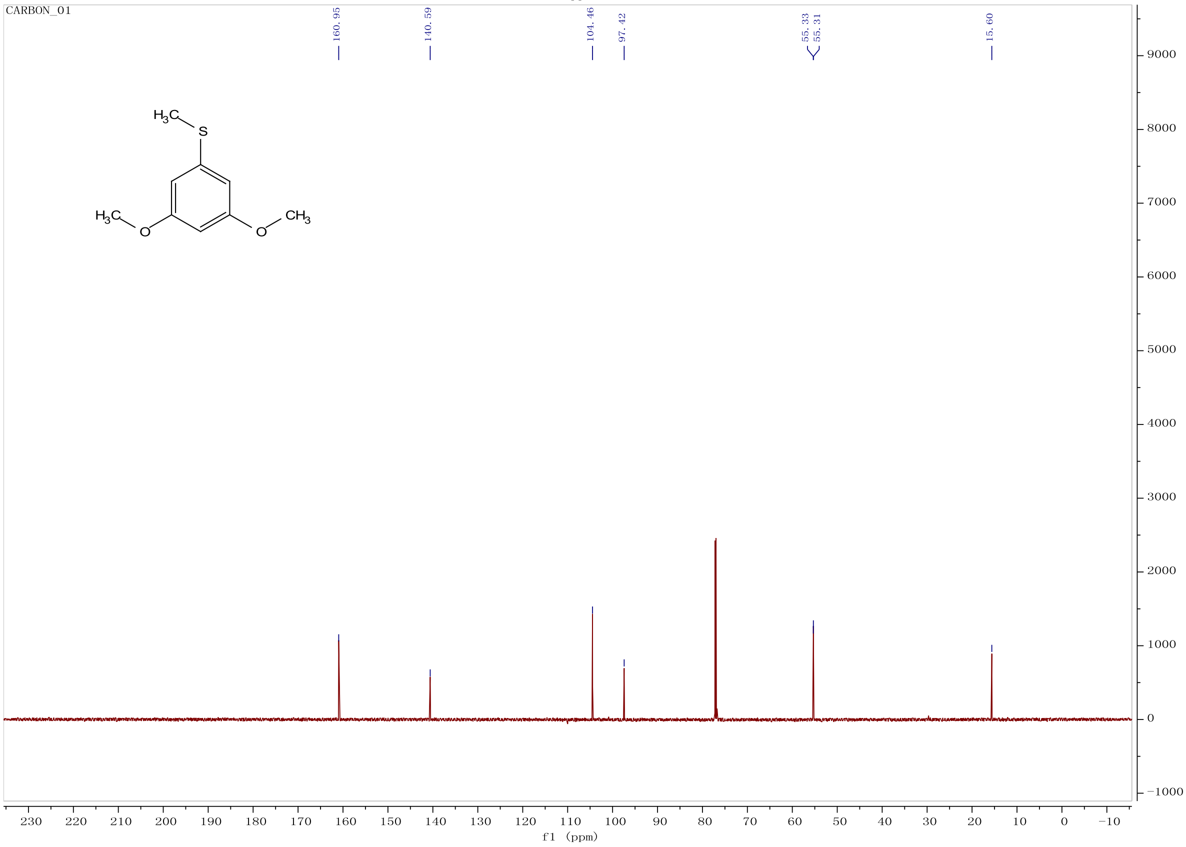

CARBON_01
160.95
140.59
104.46
97.42
55.33
55.31
15.60
H_3C S
H_3C O
O CH_3
f1 (ppm)

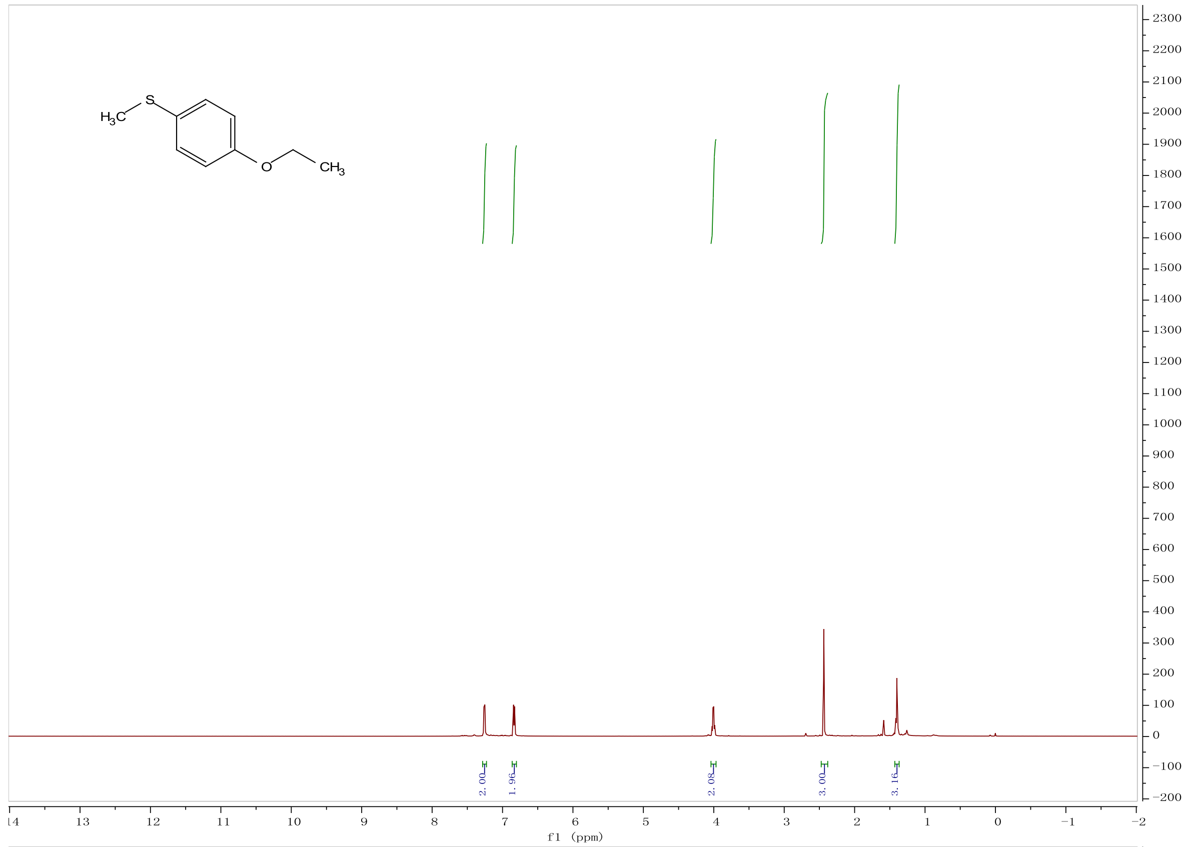
H3C S O CH3
2.00
1.96
2.08
3.00
3.16
f1 (ppm)

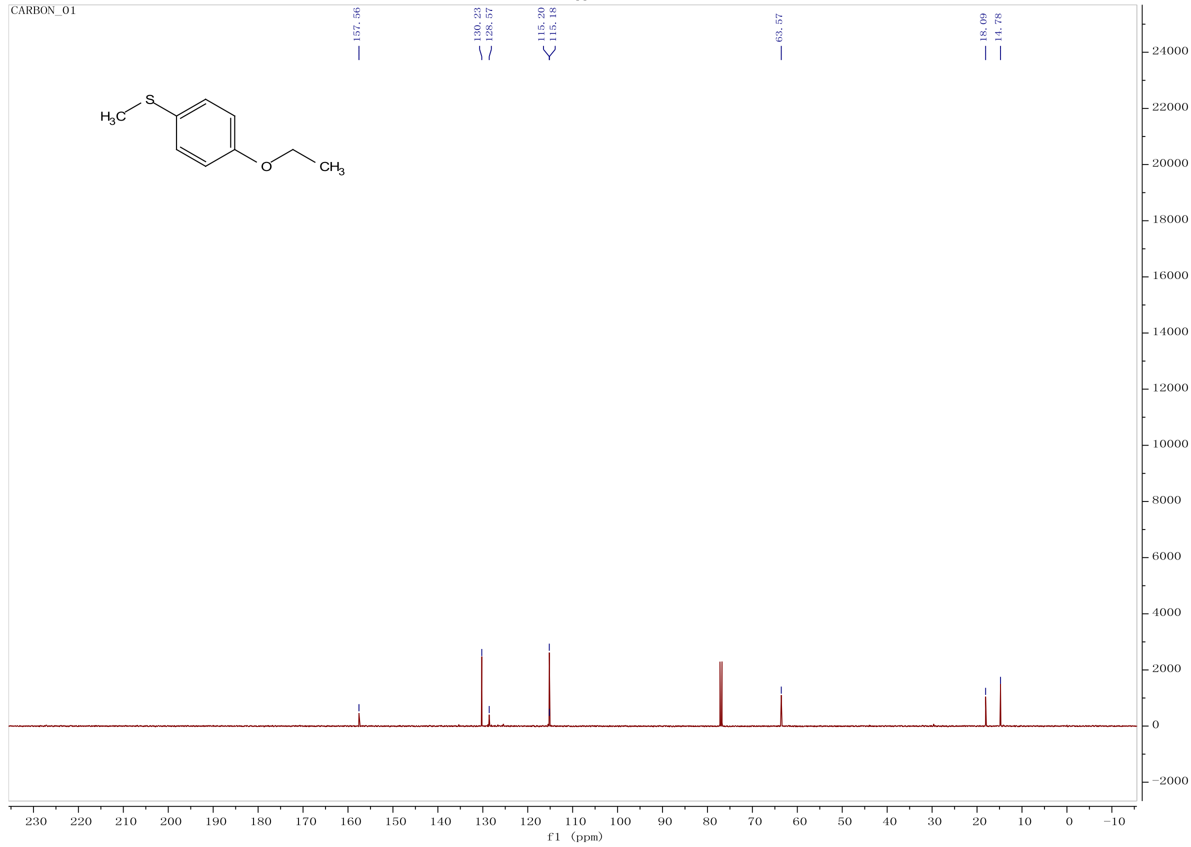
CARBON_01
157.56
130.23
128.57
115.20
115.18
63.57
18.09
14.78
H3C S O CH3
f1 (ppm)

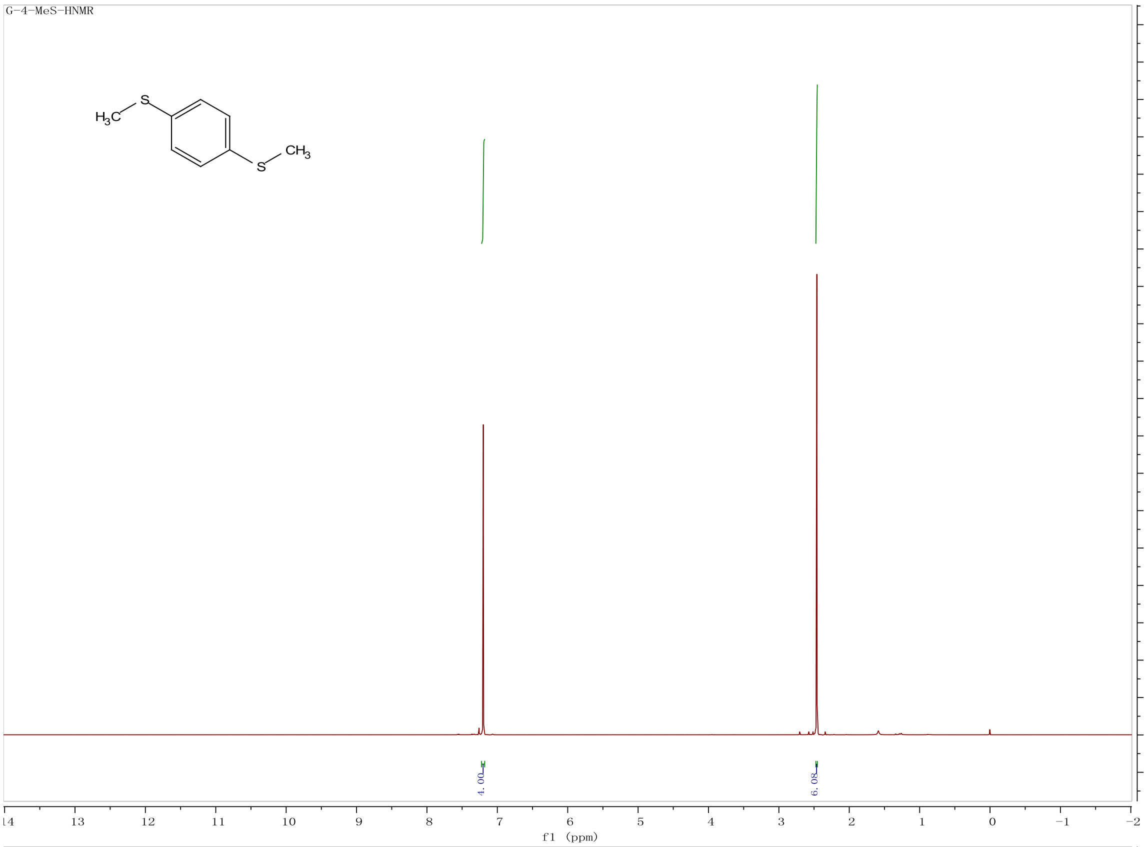
G-4-MeS-HNMR
H3C
S
S
CH3
4.00
6.08
f1 (ppm)

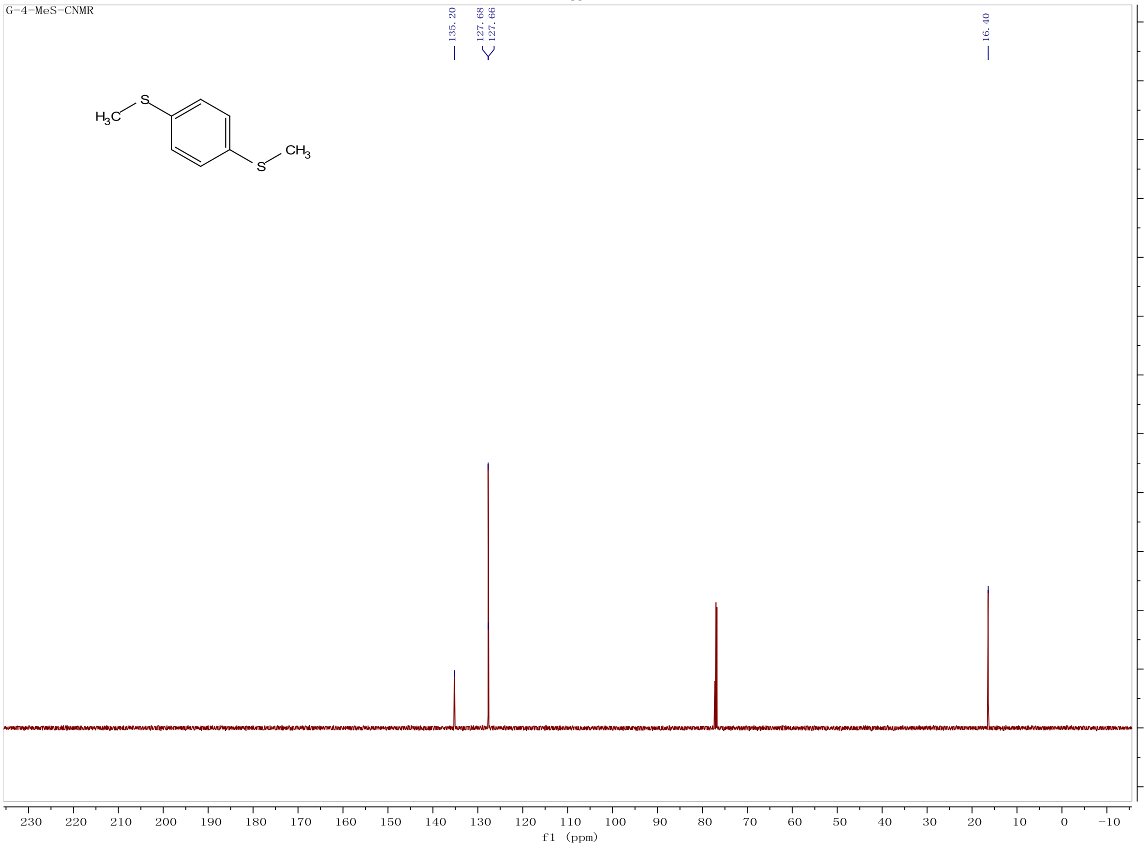
G-4-MeS-CNMR
135.20
127.68
127.66
16.40
H3C
S
S
CH3
f1 (ppm)

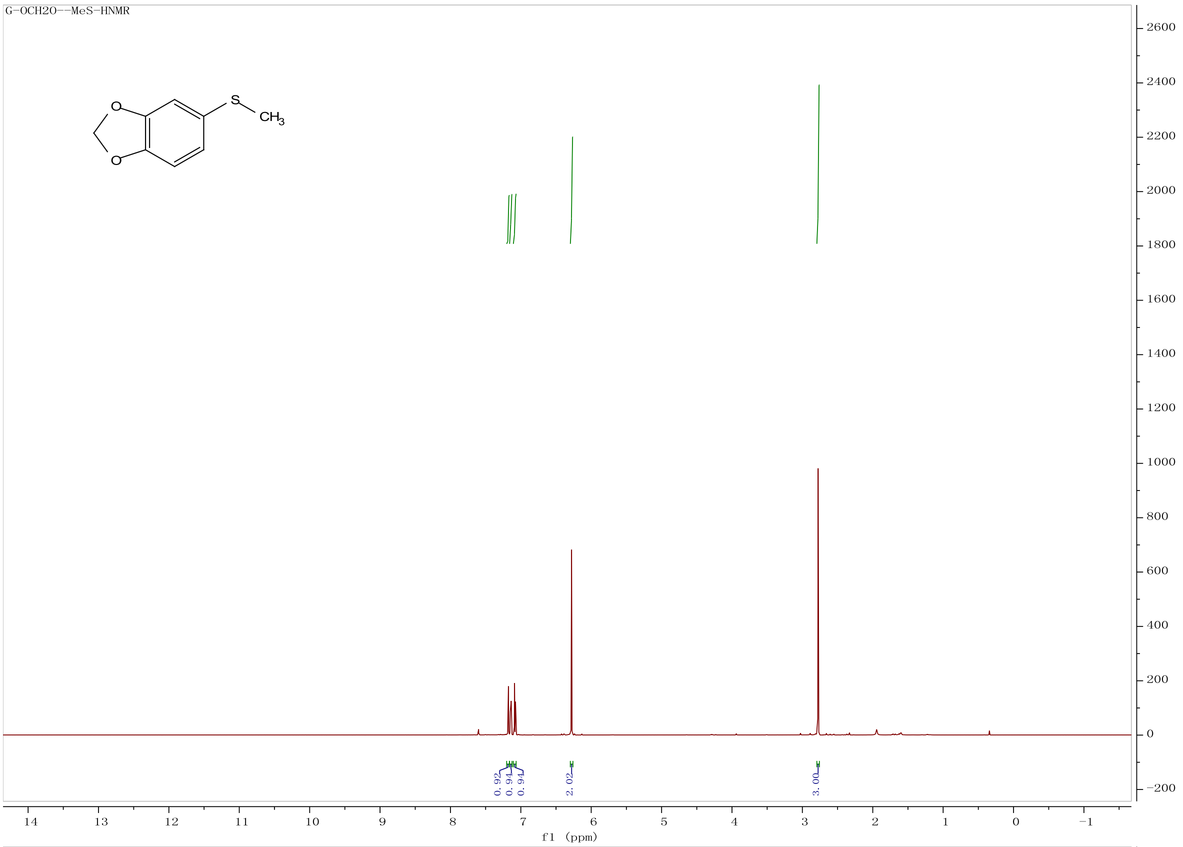
G-OCH2O--MeS-HNMR
S
CH3
O
O
0.92
0.94
0.94
2.02
3.00
f1 (ppm)

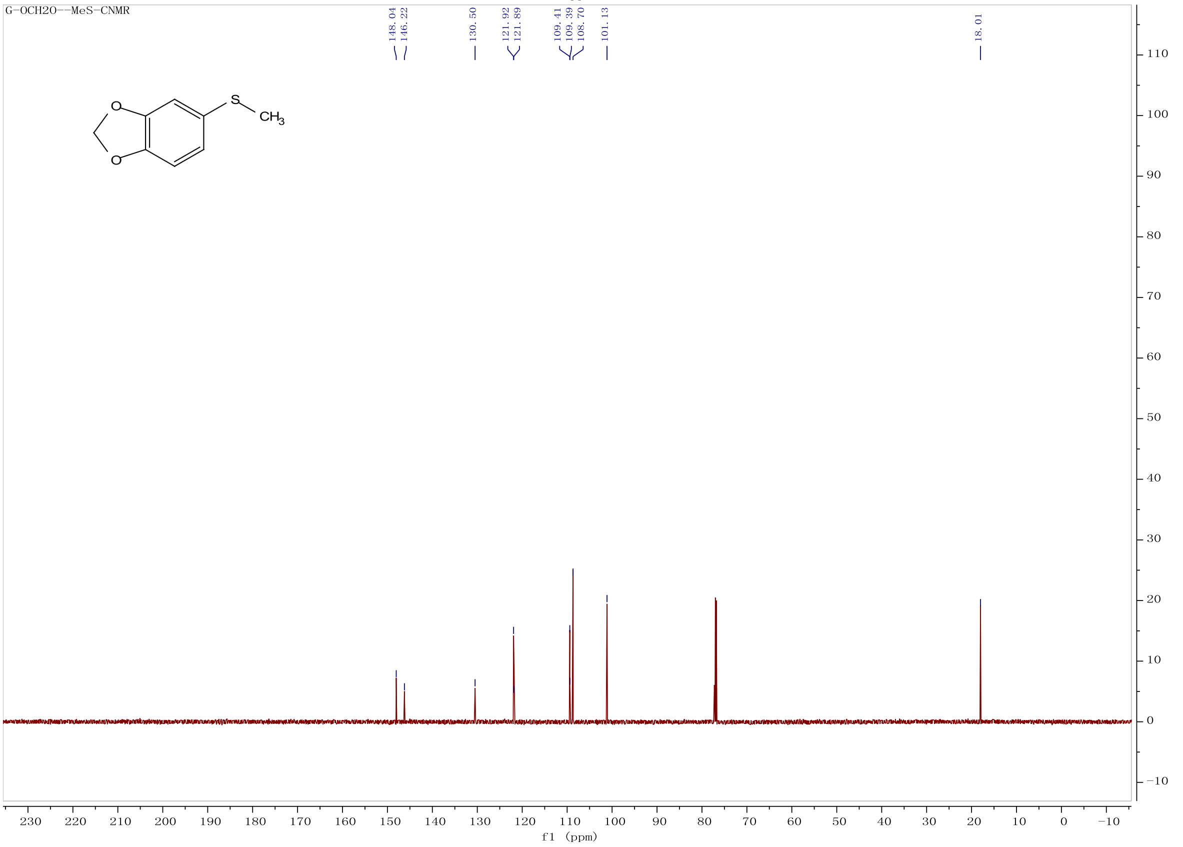
G-OCH2O--MeS-CNMR
148.04
146.22
130.50
121.92
121.89
109.41
109.39
108.70
101.13
18.01
S
CH3
O
O
f1 (ppm)

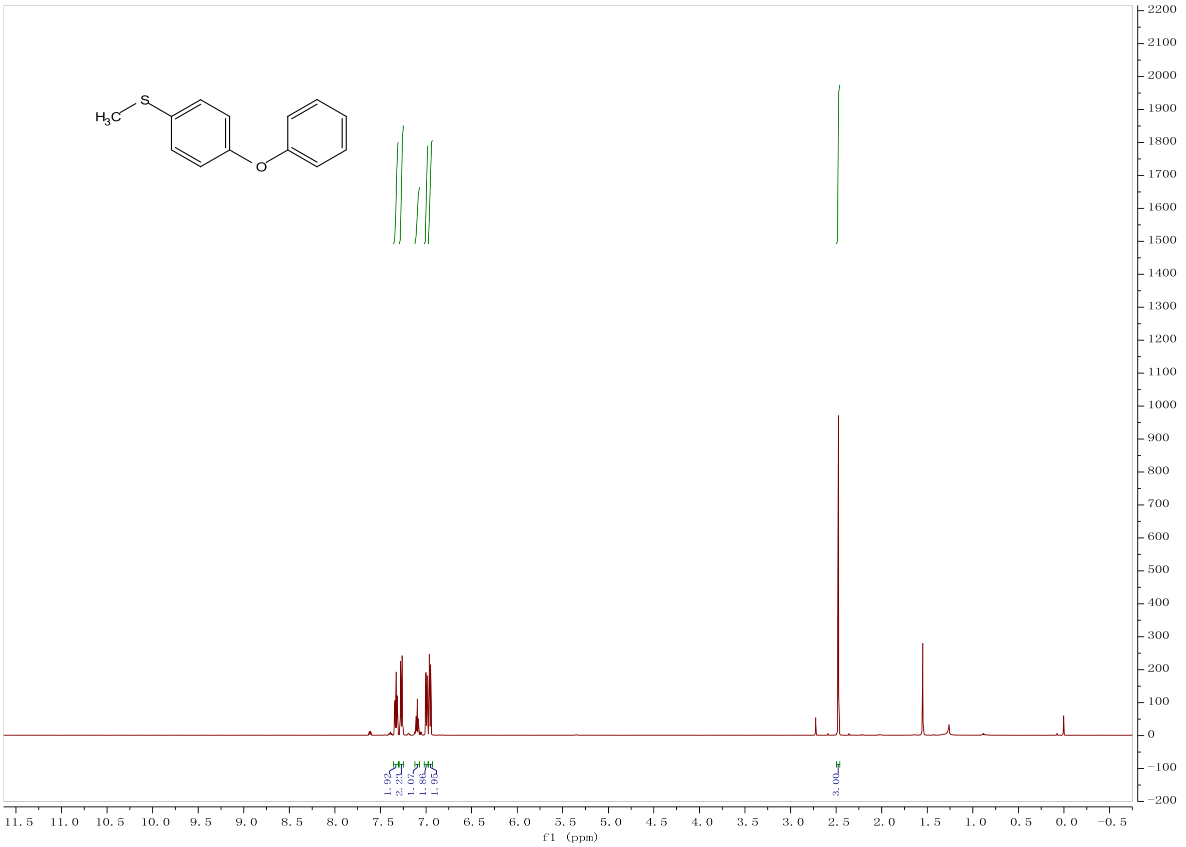
H3C
S
O
1.92
2.23
1.07
1.86
1.95
3.00
f1 (ppm)

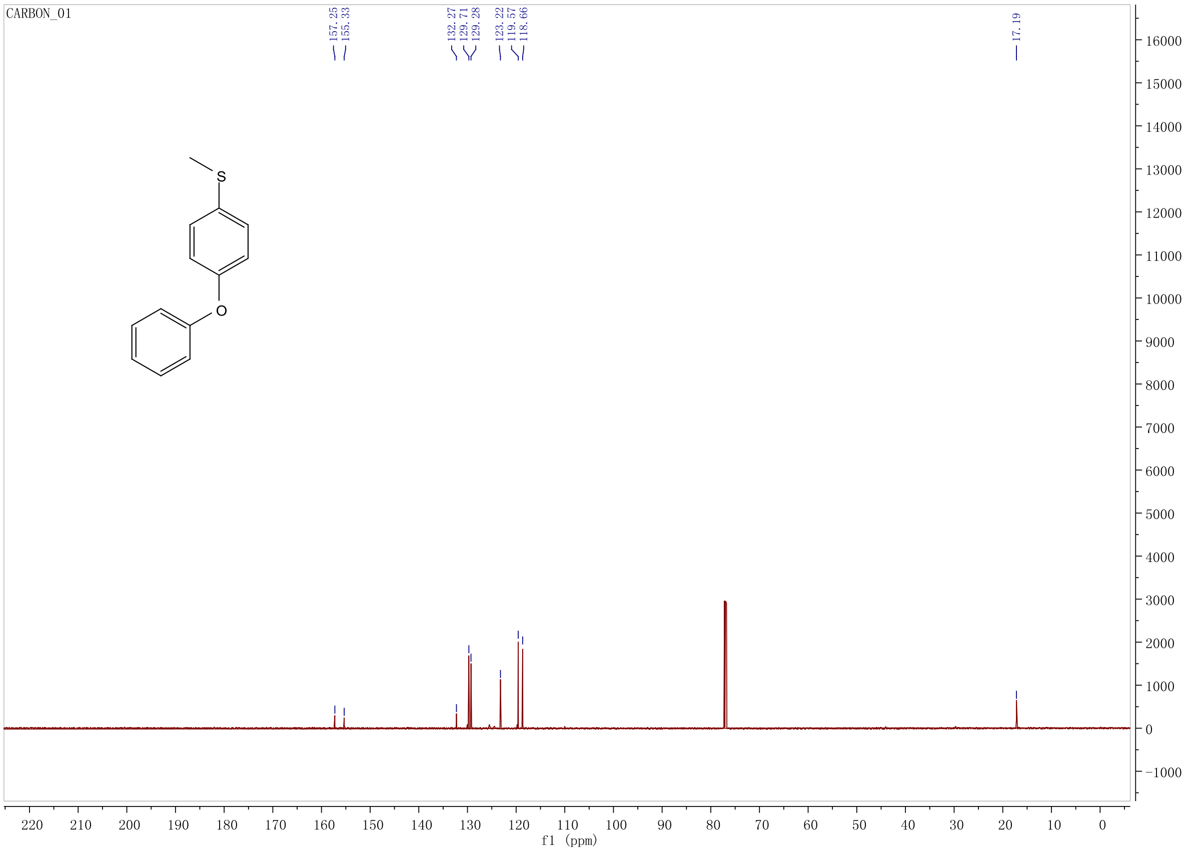
CARBON_01
157.25
155.33
132.27
129.71
129.28
123.22
119.57
118.66
17.19
S
O
f1 (ppm)

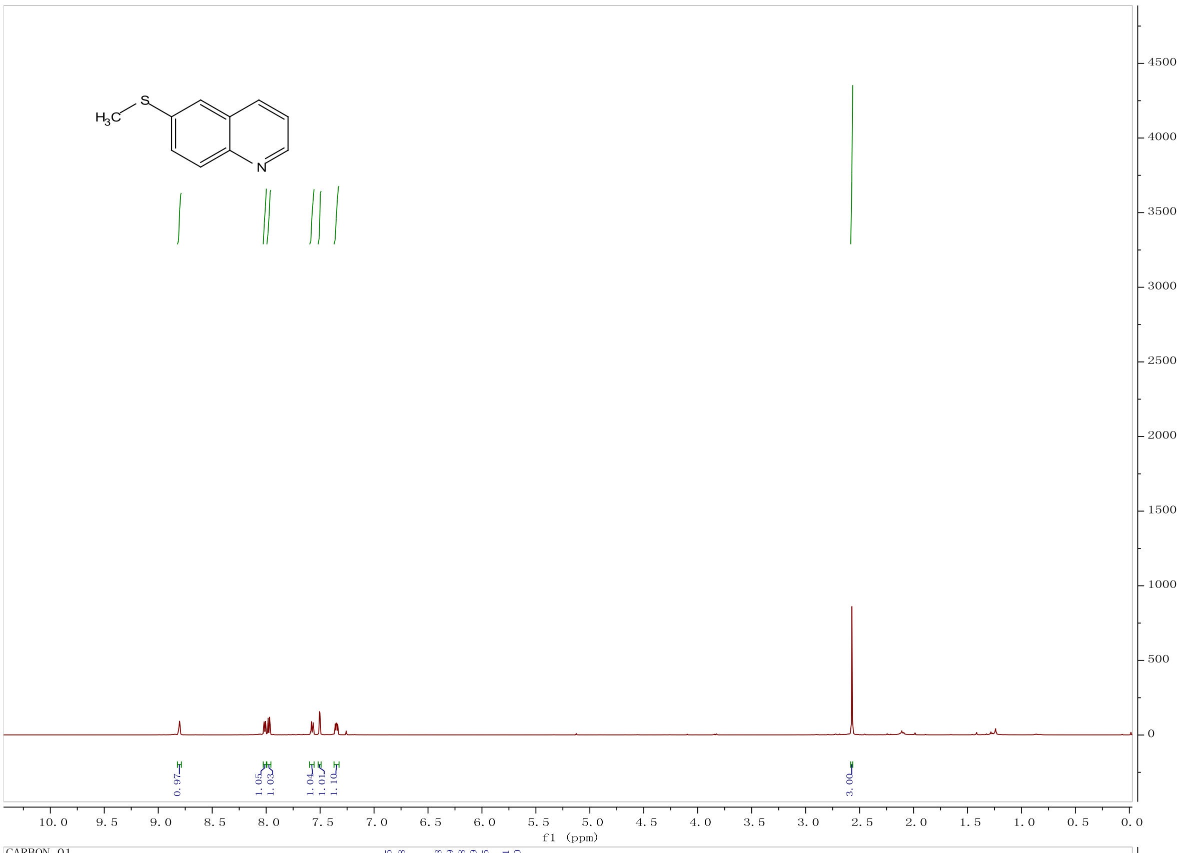
H3C S N
0.97
1.05
1.03
1.04
1.01
1.10
3.00
f1 (ppm)

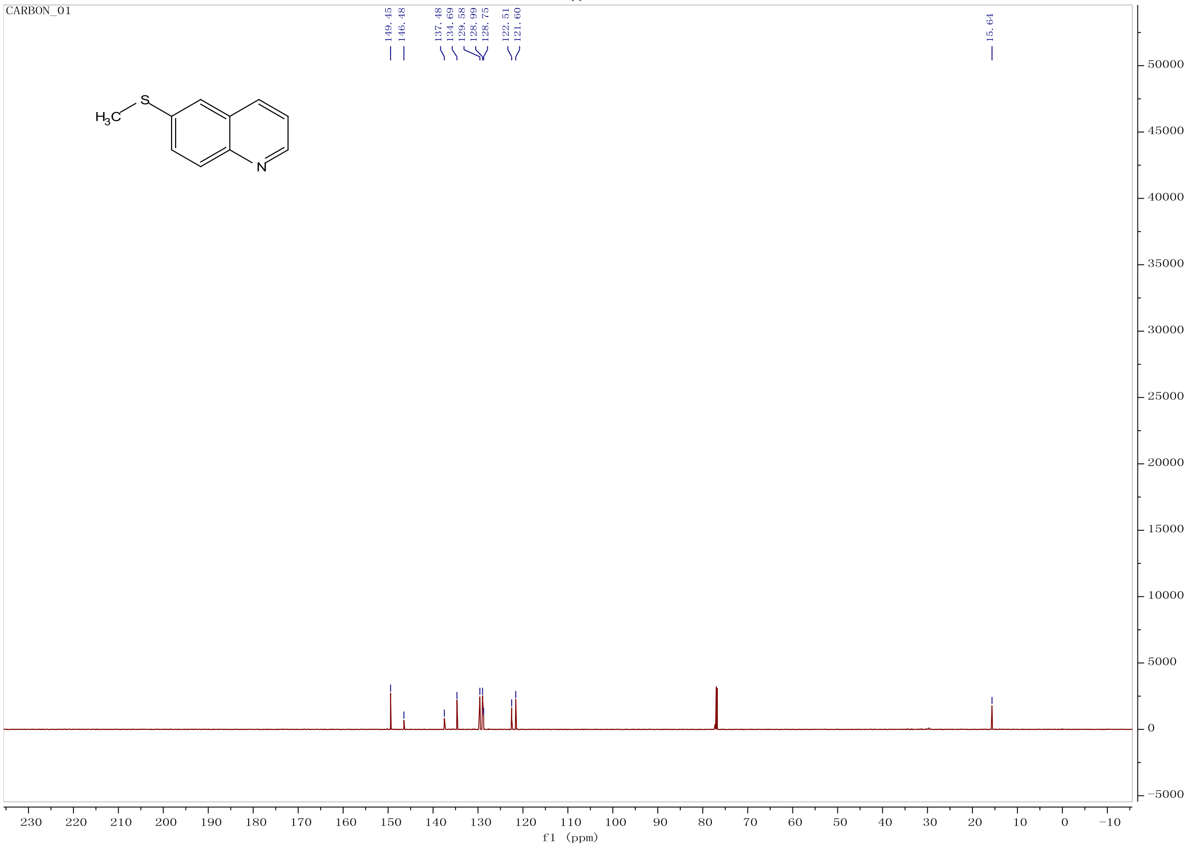
CARBON_01
149.45
146.48
137.48
134.69
129.58
128.99
128.75
122.51
121.60
15.64
H3C S N
f1 (ppm)

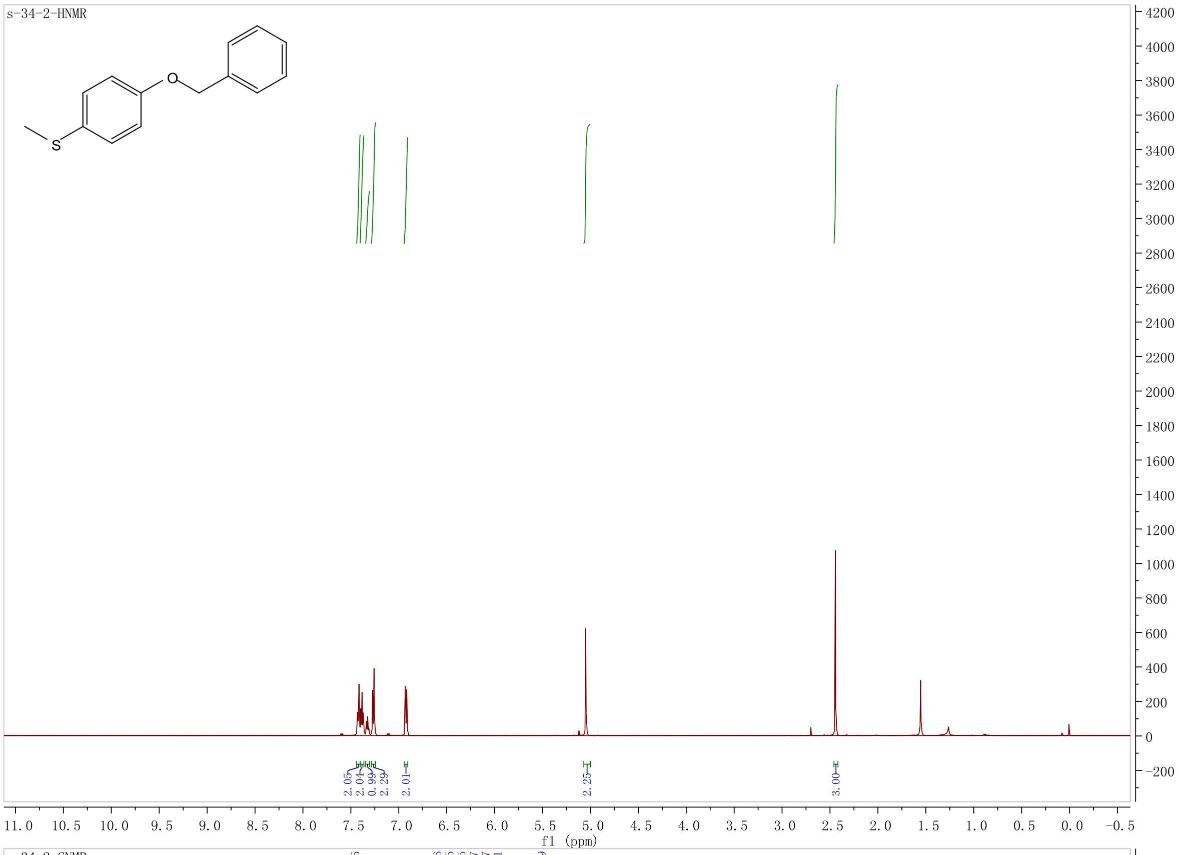
s-34-2-HNMR
S
O
2.05
2.04
0.99
2.29
2.01
2.25
3.00
f1 (ppm)

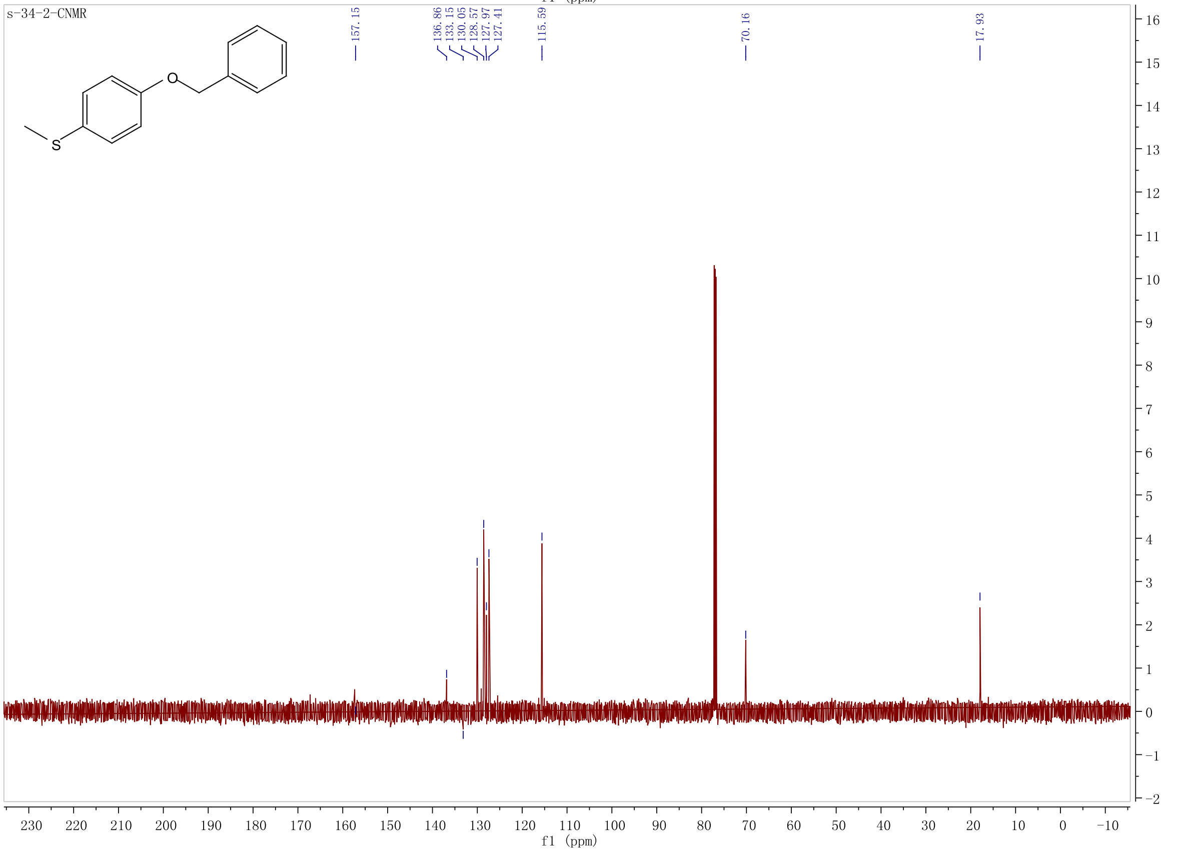
s-34-2-CNMR
S
O
157.15
136.86
133.15
130.05
128.57
127.97
127.41
115.59
70.16
17.93
f1 (ppm)

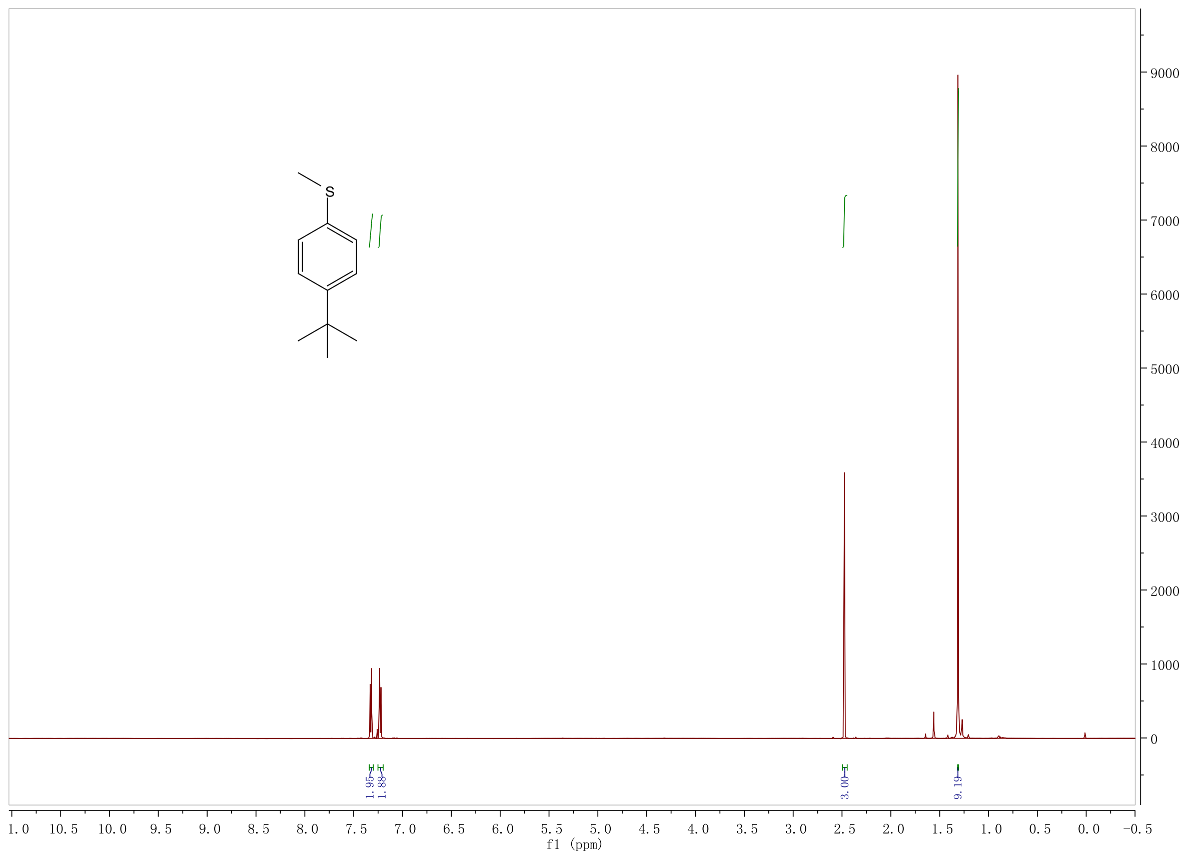

S
1.95
1.88
3.00
9.19
f1 (ppm)

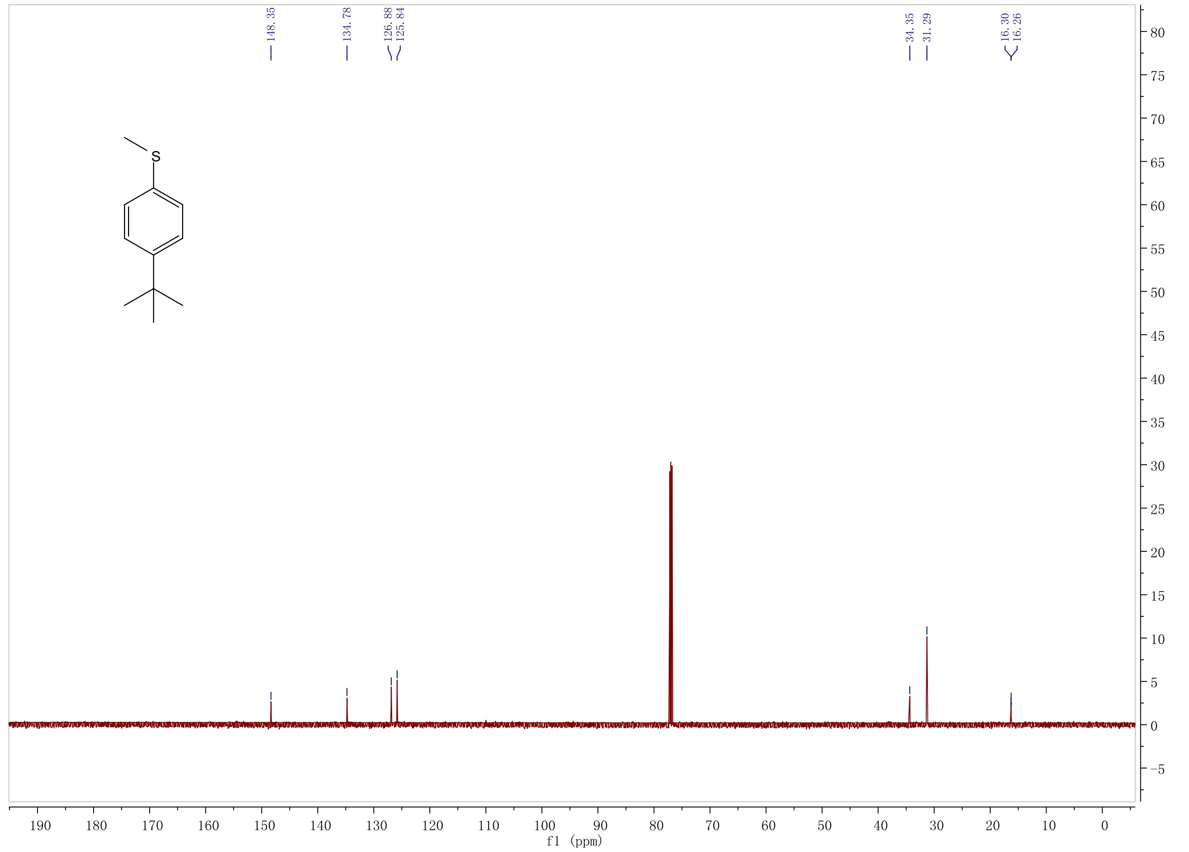

148.35
134.78
126.88
125.84
34.35
31.29
16.30
16.26
S
f1 (ppm)

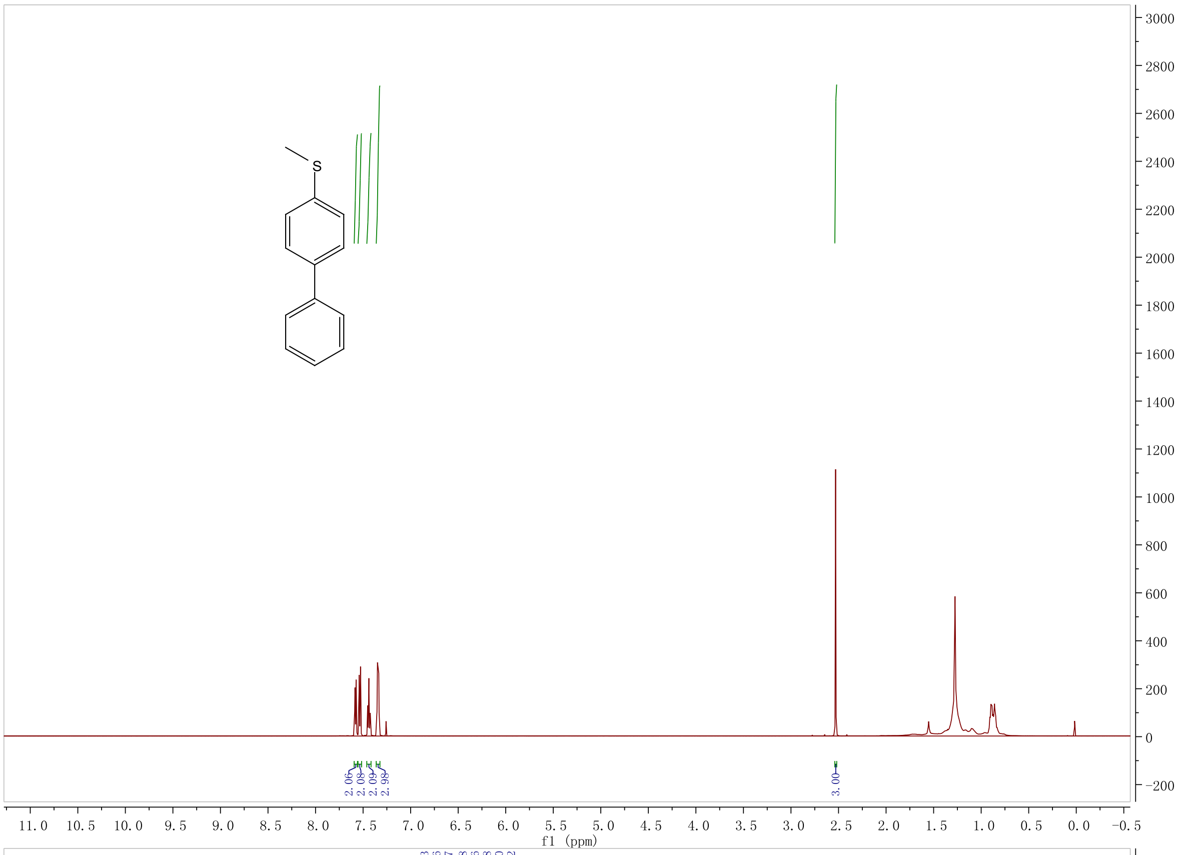
S
2.06
2.08
2.09
2.98
3.00
f1 (ppm)

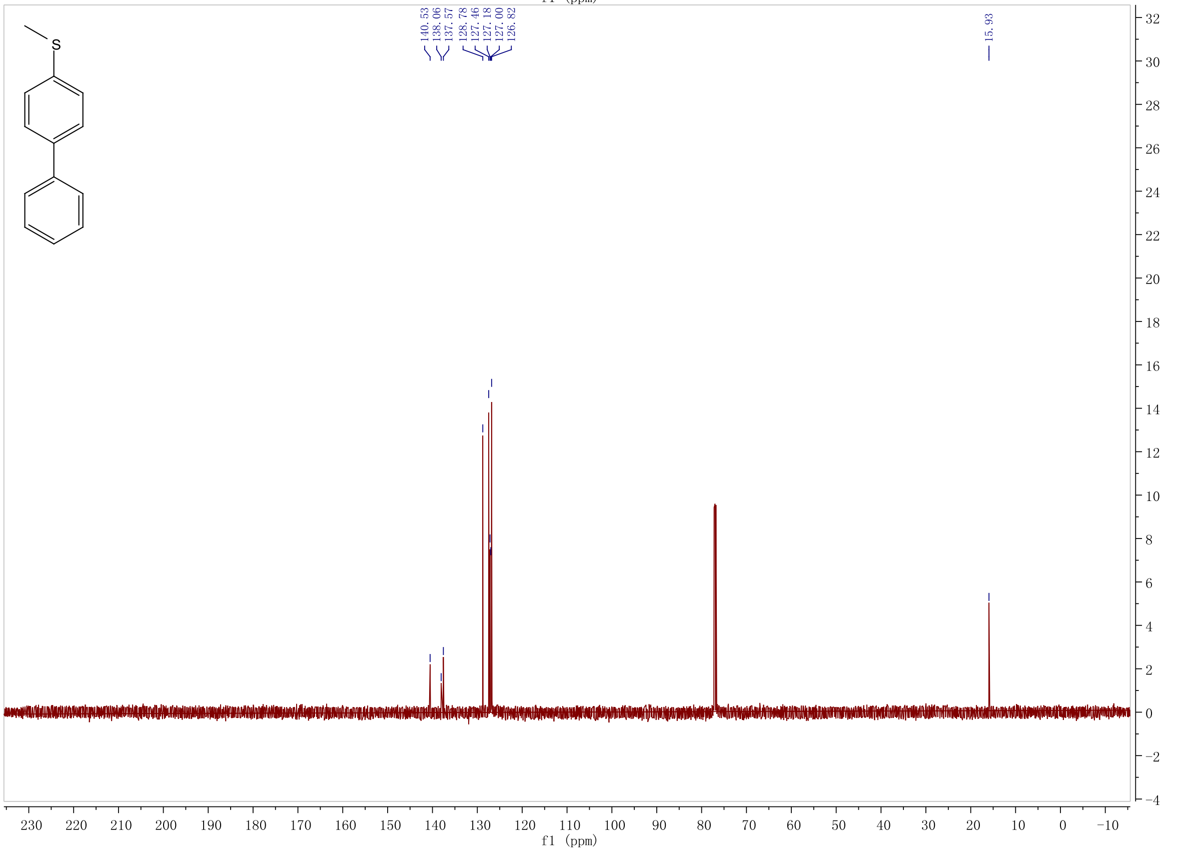
S
140.53
138.06
137.57
128.78
127.46
127.18
127.00
126.82
15.93
f1 (ppm)

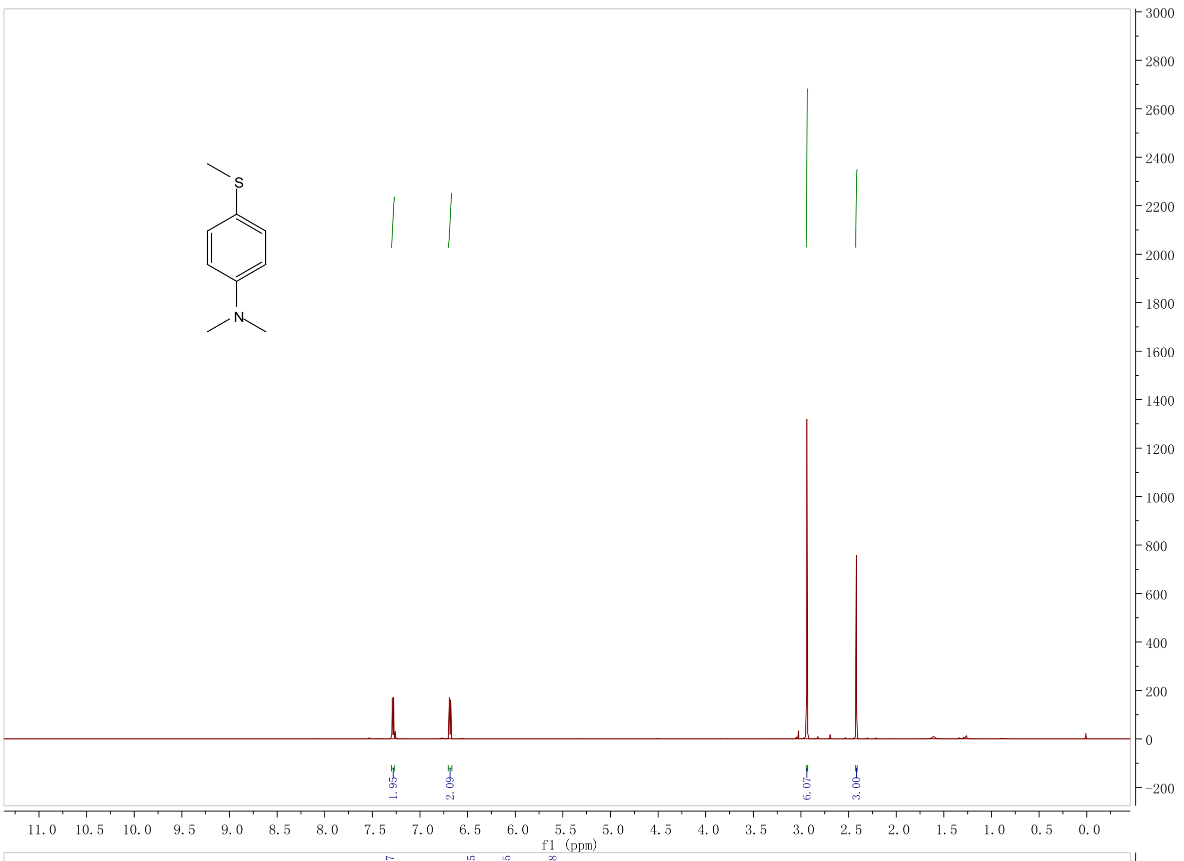

S
N
1.95
2.09
6.07
3.00
f1 (ppm)

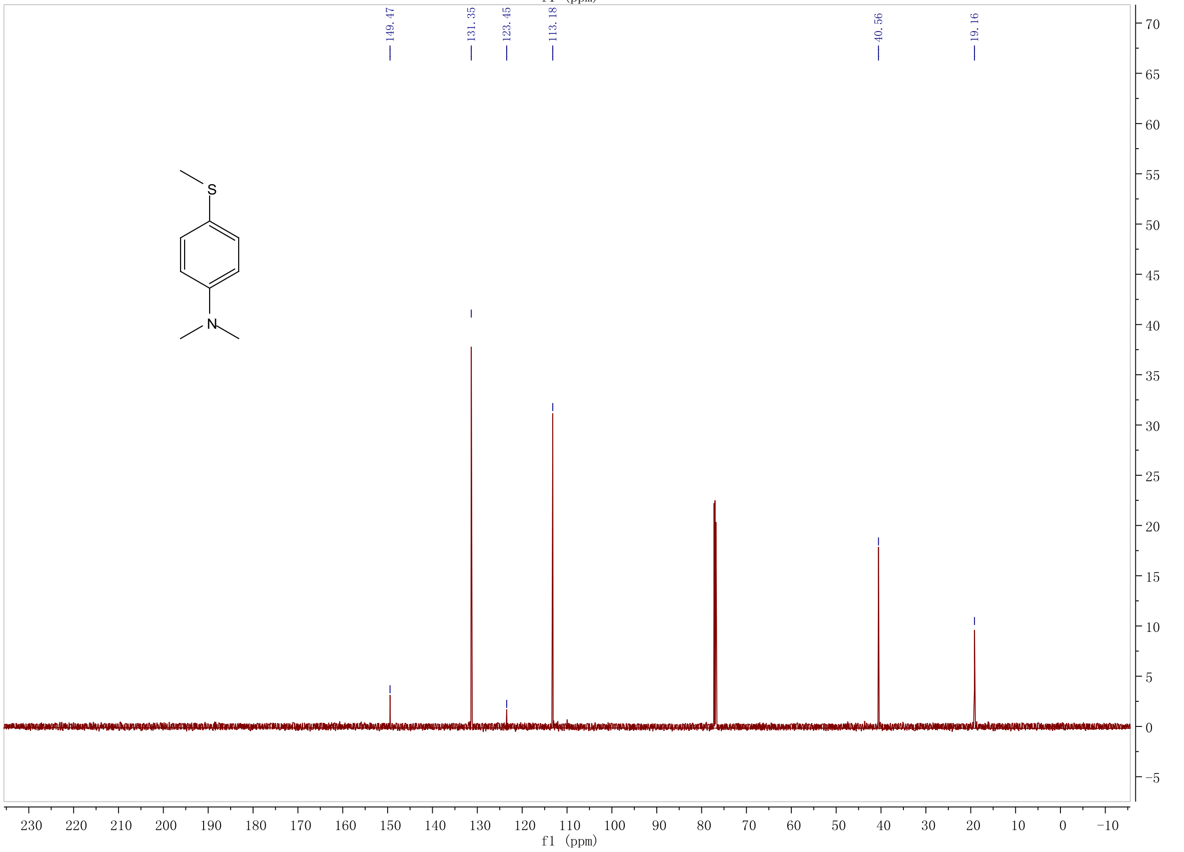

149.47
131.35
123.45
113.18
40.56
19.16
S
N
f1 (ppm)

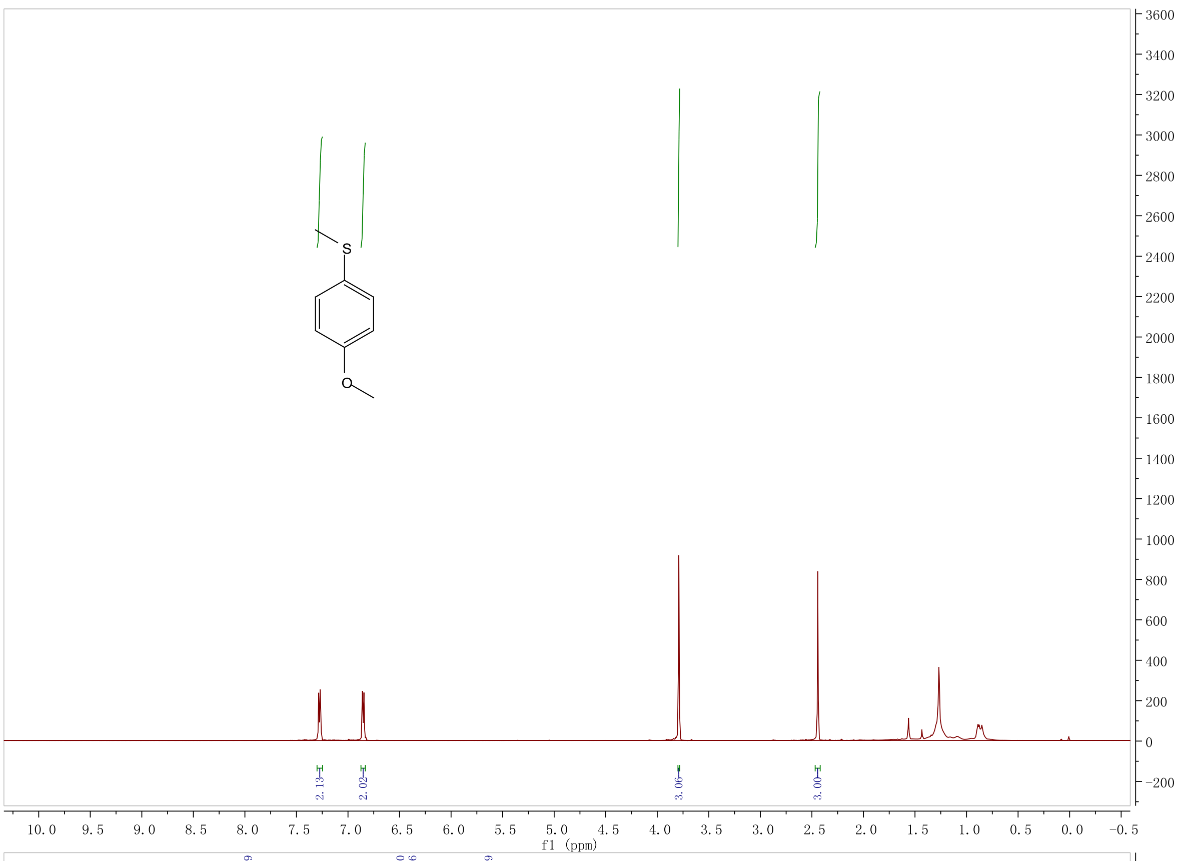
S
O
2.13
2.02
3.06
3.00
f1 (ppm)

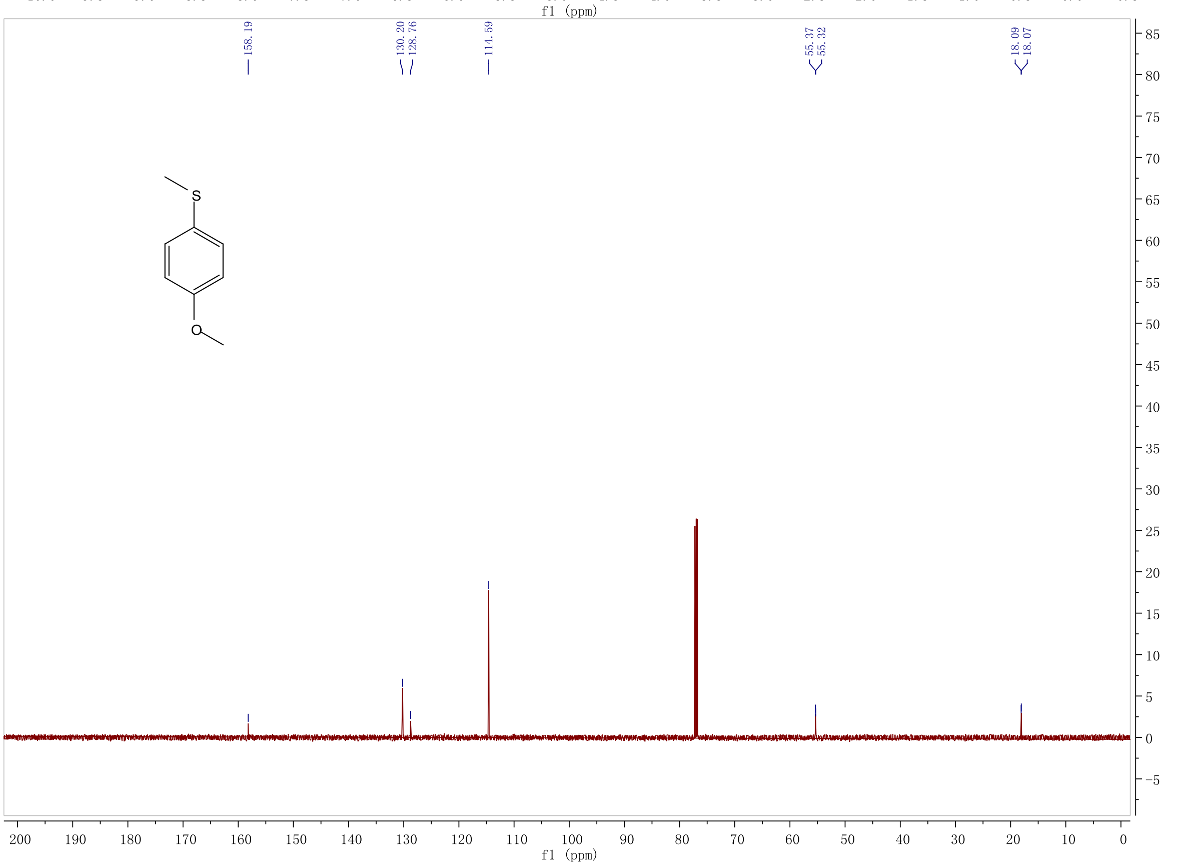
158.19
130.20
128.76
114.59
55.37
55.32
18.09
18.07
S
O
f1 (ppm)

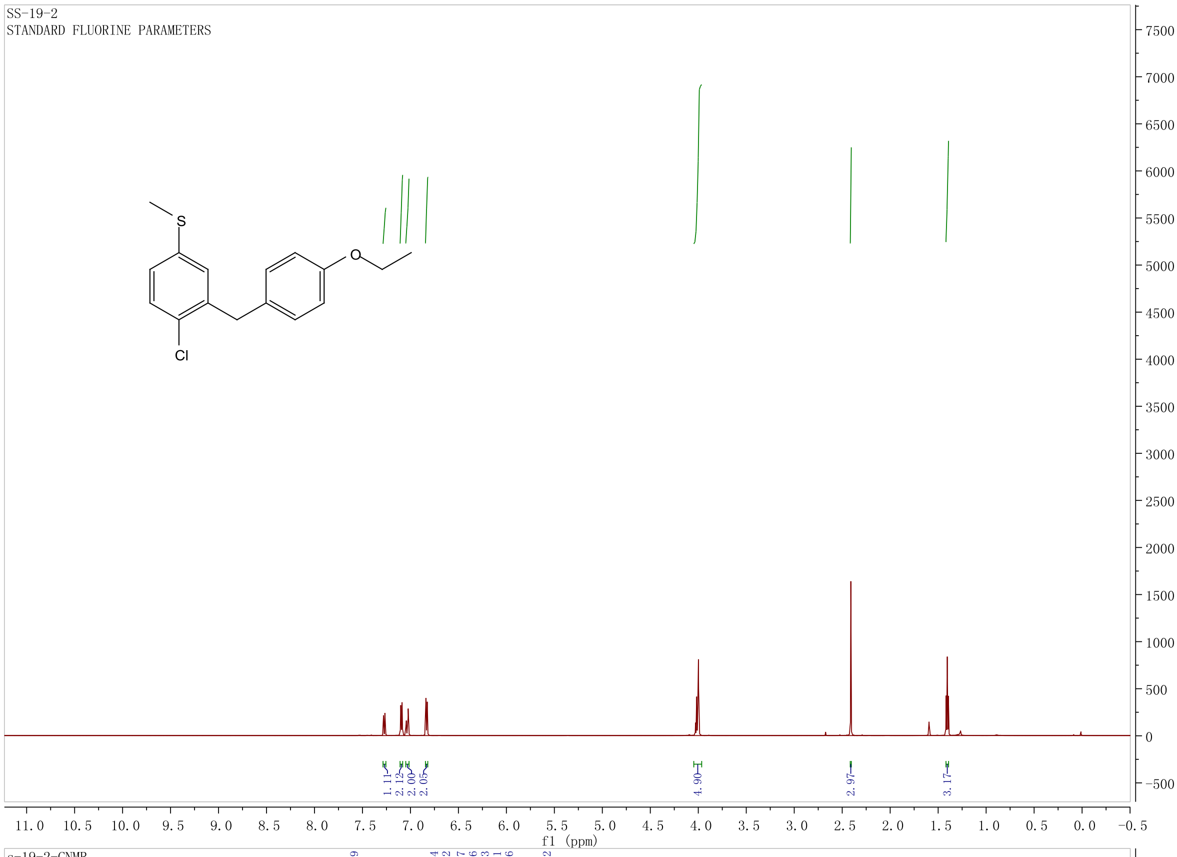
SS-19-2
STANDARD FLUORINE PARAMETERS
S
O
Cl
1.11
2.12
2.00
2.05
4.90
2.97
3.17
f1 (ppm)

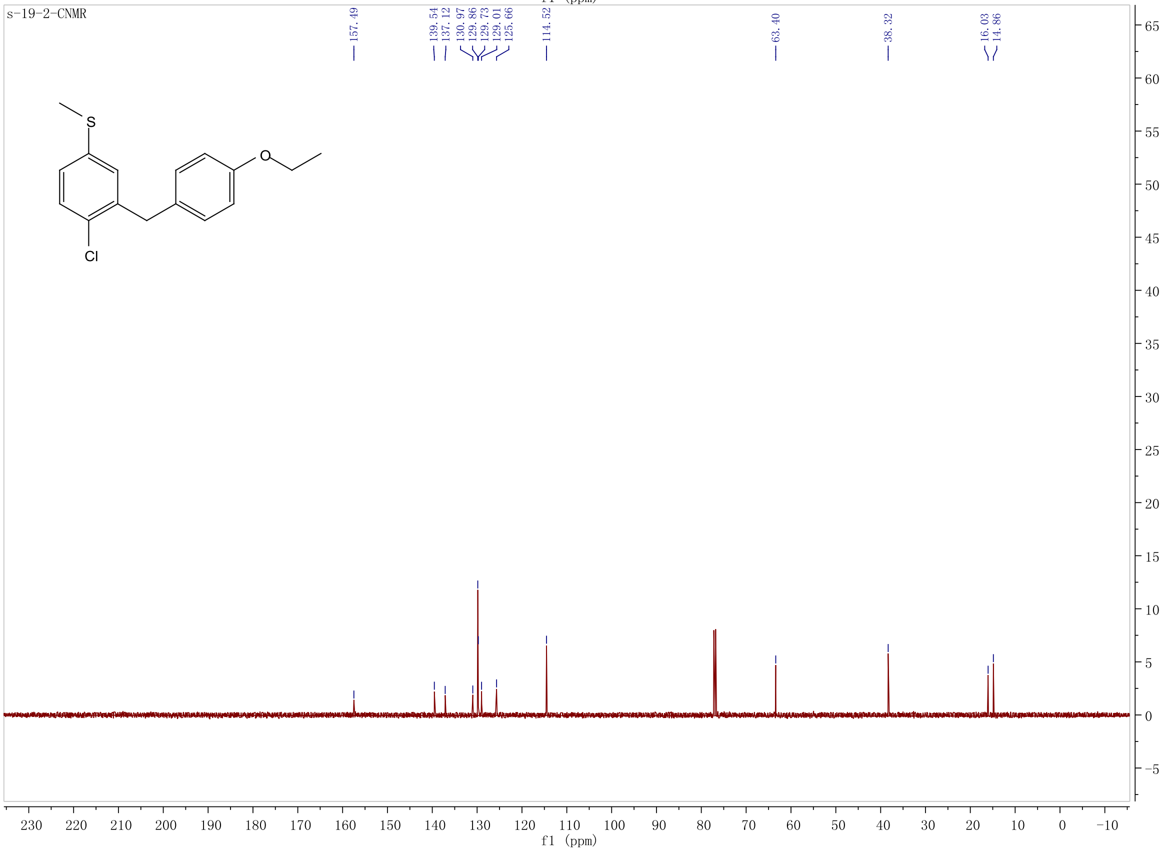
s-19-2-CNMR
157.49
139.54
137.12
130.97
129.86
129.73
129.01
125.66
114.52
63.40
38.32
16.03
14.86
S
O
Cl
f1 (ppm)

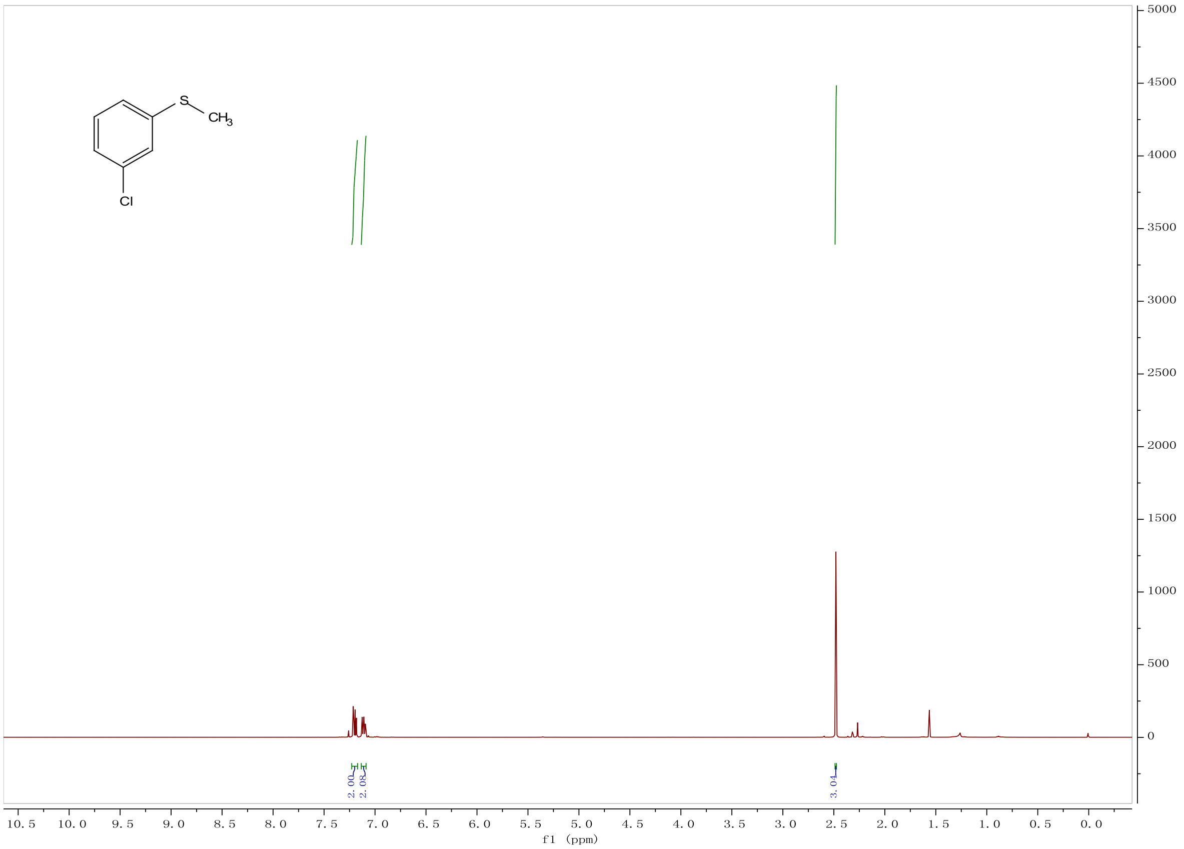
S
CH3
Cl
2.00
2.08
3.04
f1 (ppm)

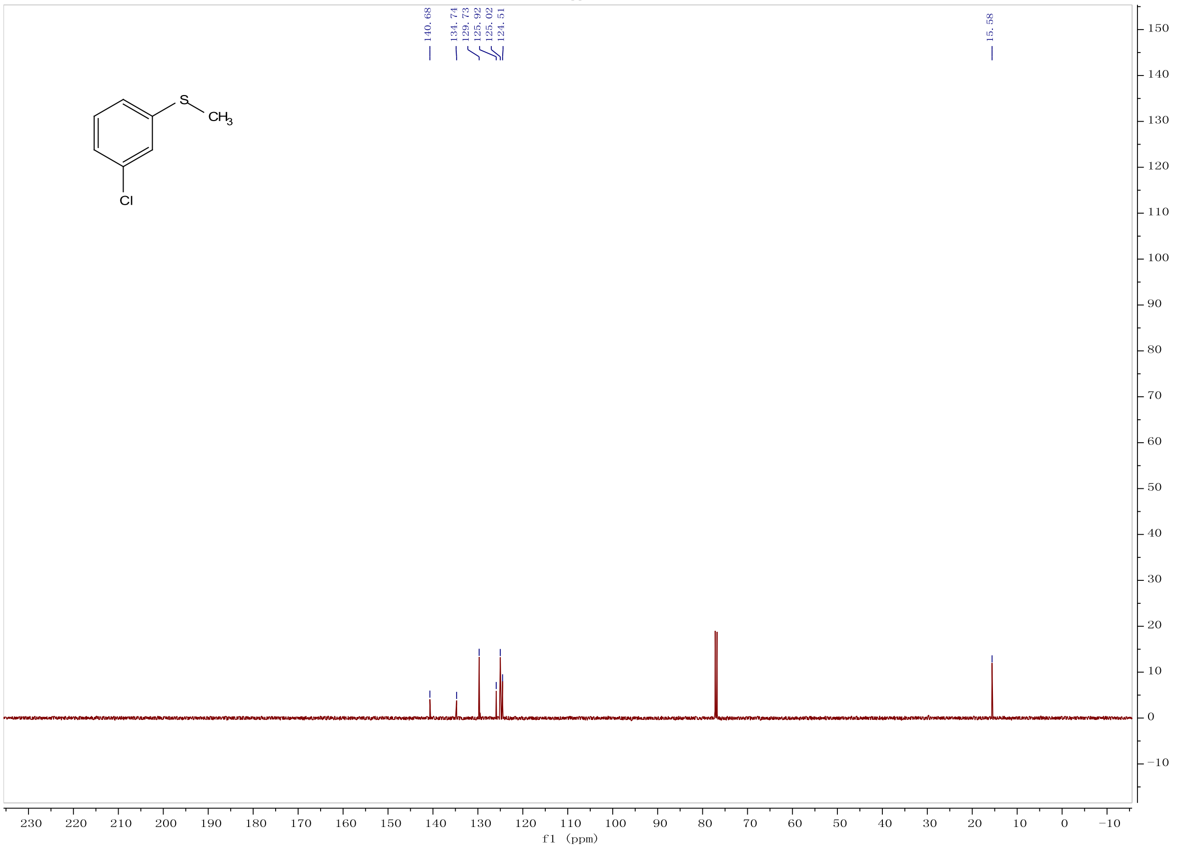
140.68
134.74
129.73
125.92
125.02
124.51
15.58
S
CH3
Cl
f1 (ppm)

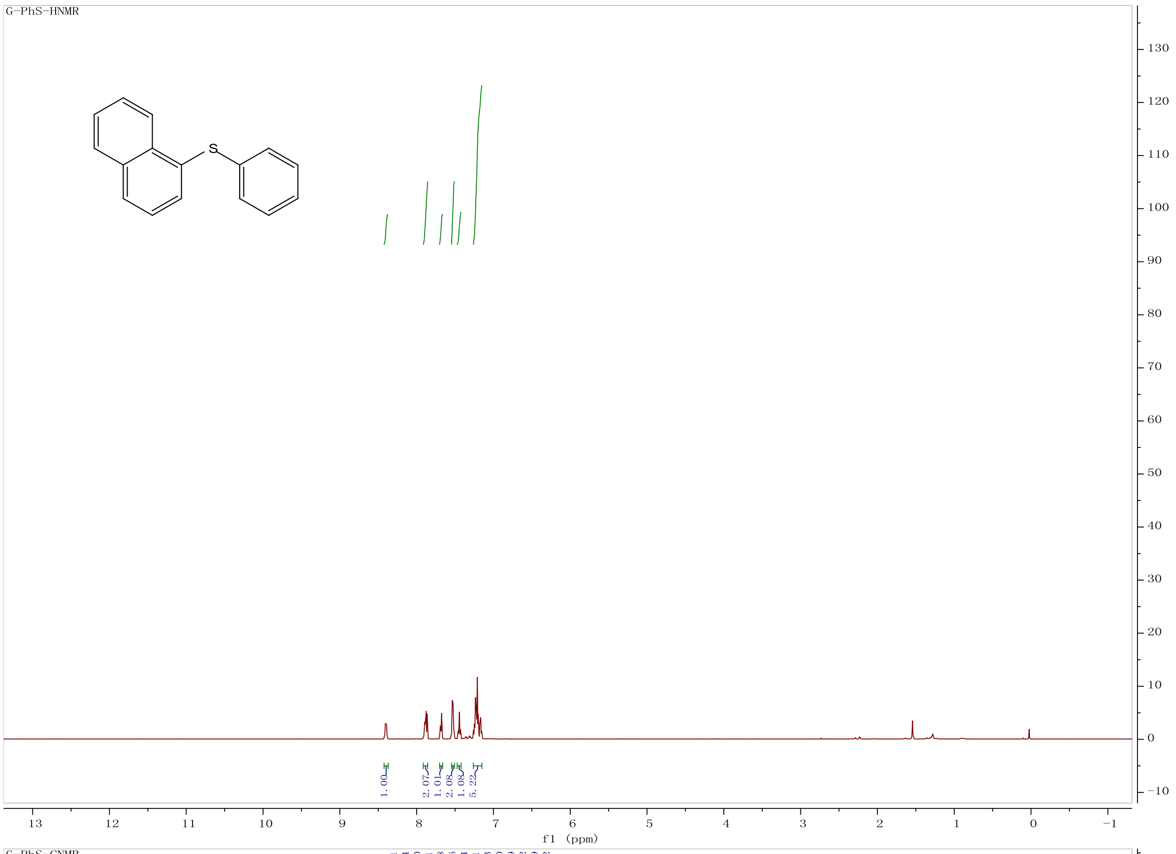
G-PhS-HNMR
S
1.00
2.07
1.01
2.08
1.08
5.22
f1 (ppm)

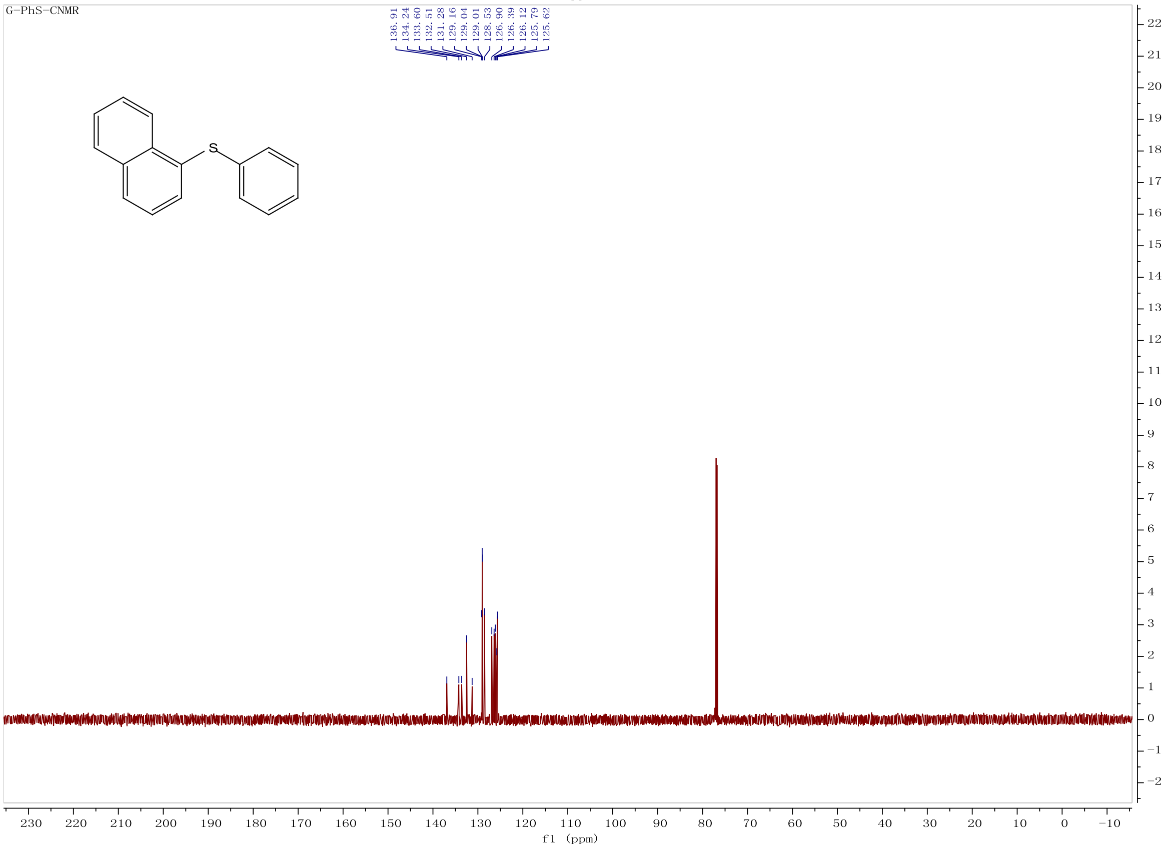
G-PhS-CNMR
136.91
134.24
133.60
132.51
131.28
129.16
129.04
129.01
128.53
126.90
126.39
126.12
125.79
125.62
S
f1 (ppm)

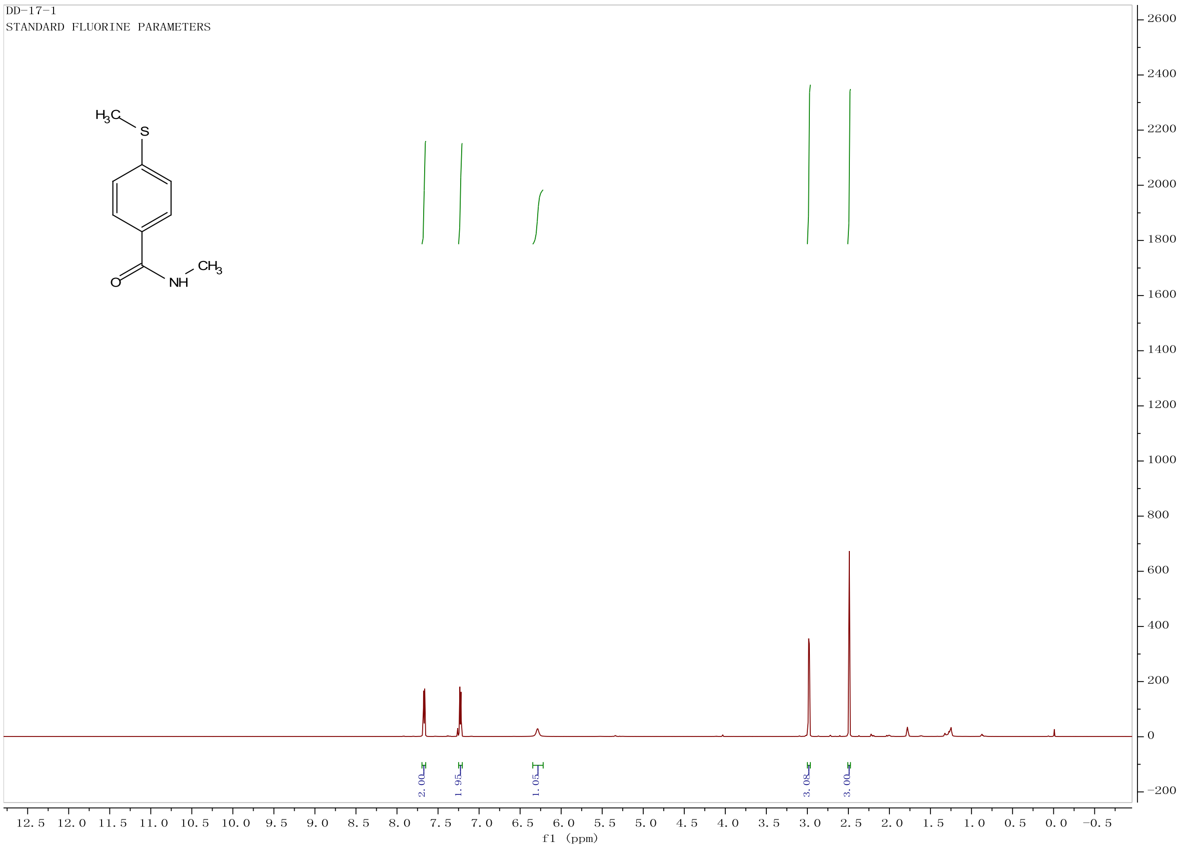
DD-17-1
STANDARD FLUORINE PARAMETERS
H3C
S
O
NH
CH3
2.00
1.95
1.05
3.08
3.00
12.5 12.0 11.5 11.0 10.5 10.0 9.5 9.0 8.5 8.0 7.5 7.0 6.5 6.0 5.5 5.0 4.5 4.0 3.5 3.0 2.5 2.0 1.5 1.0 0.5 0.0 -0.5
f1 (ppm)
2600 2400 2200 2000 1800 1600 1400 1200 1000 800 600 400 200 0 -200

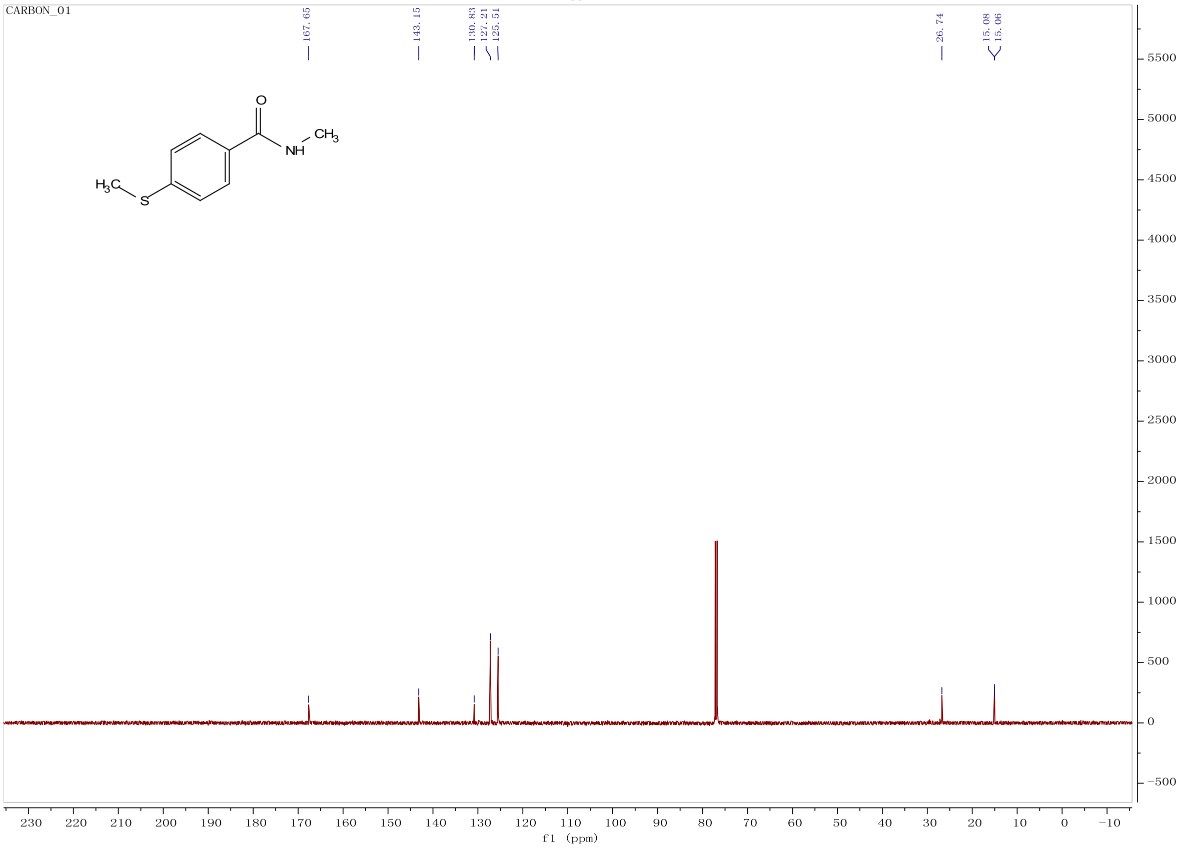
CARBON_01
167.65
143.15
130.83
127.21
125.51
26.74
15.08
15.06
O
NH
CH3
H3C
S
230 220 210 200 190 180 170 160 150 140 130 120 110 100 90 80 70 60 50 40 30 20 10 0 -10
f1 (ppm)
5500 5000 4500 4000 3500 3000 2500 2000 1500 1000 500 0 -500

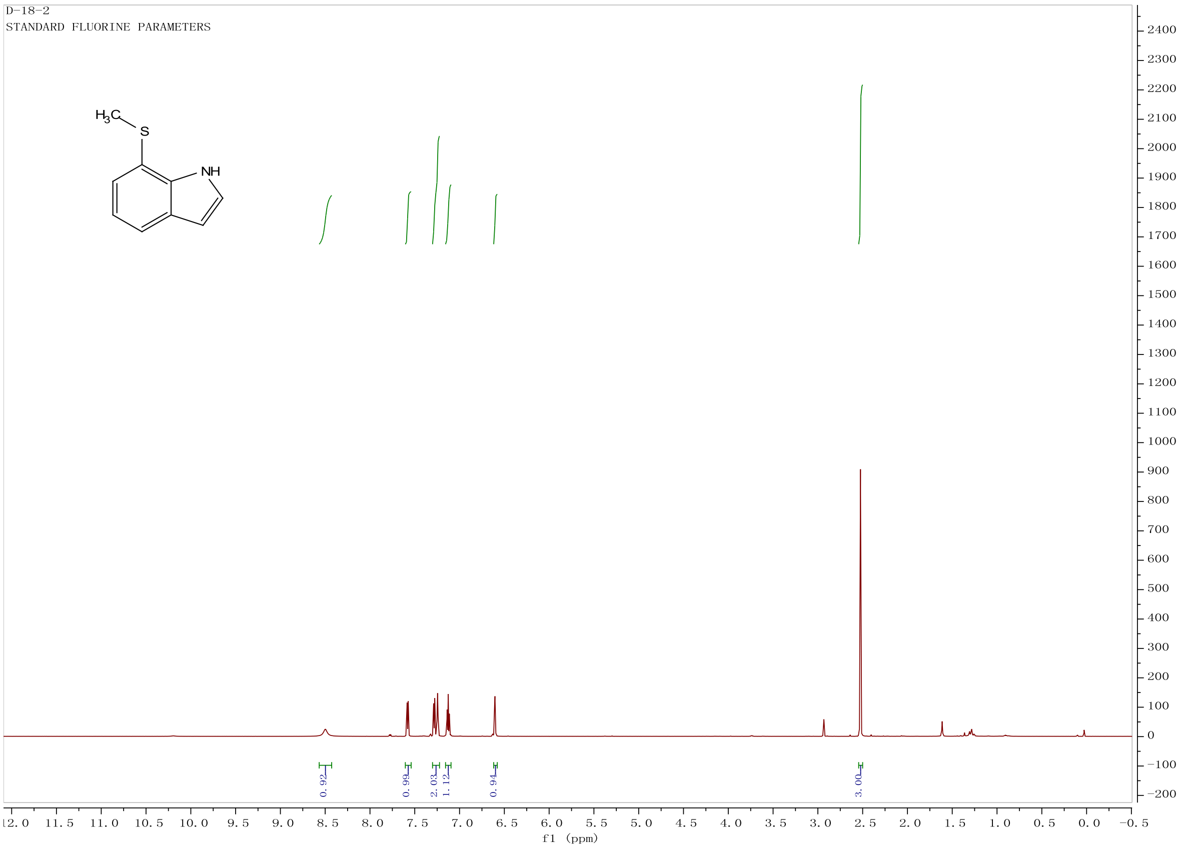
D-18-2
STANDARD FLUORINE PARAMETERS
H3C
S
NH
0.92
0.99
2.03
1.12
0.94
3.00
f1 (ppm)

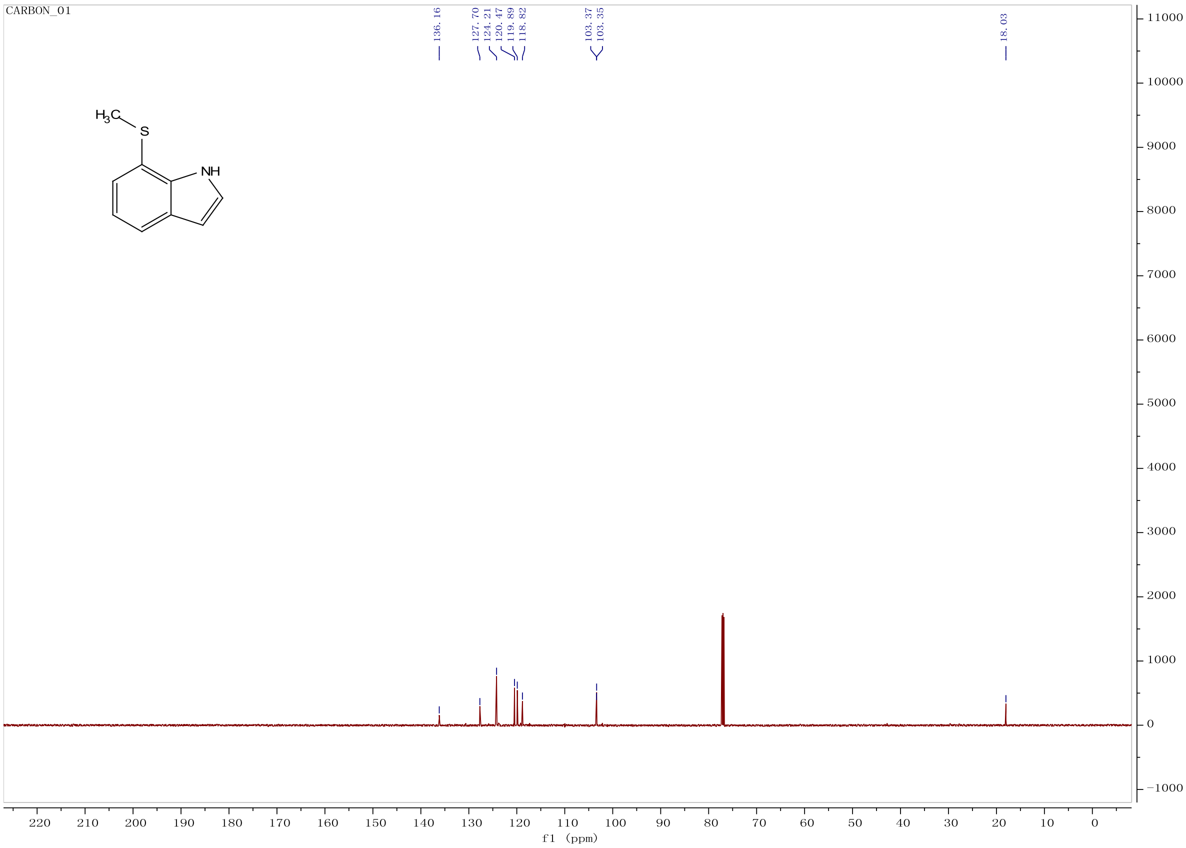
CARBON_01
136.16
127.70
124.21
120.47
119.89
118.82
103.37
103.35
18.03
H3C
S
NH
f1 (ppm)

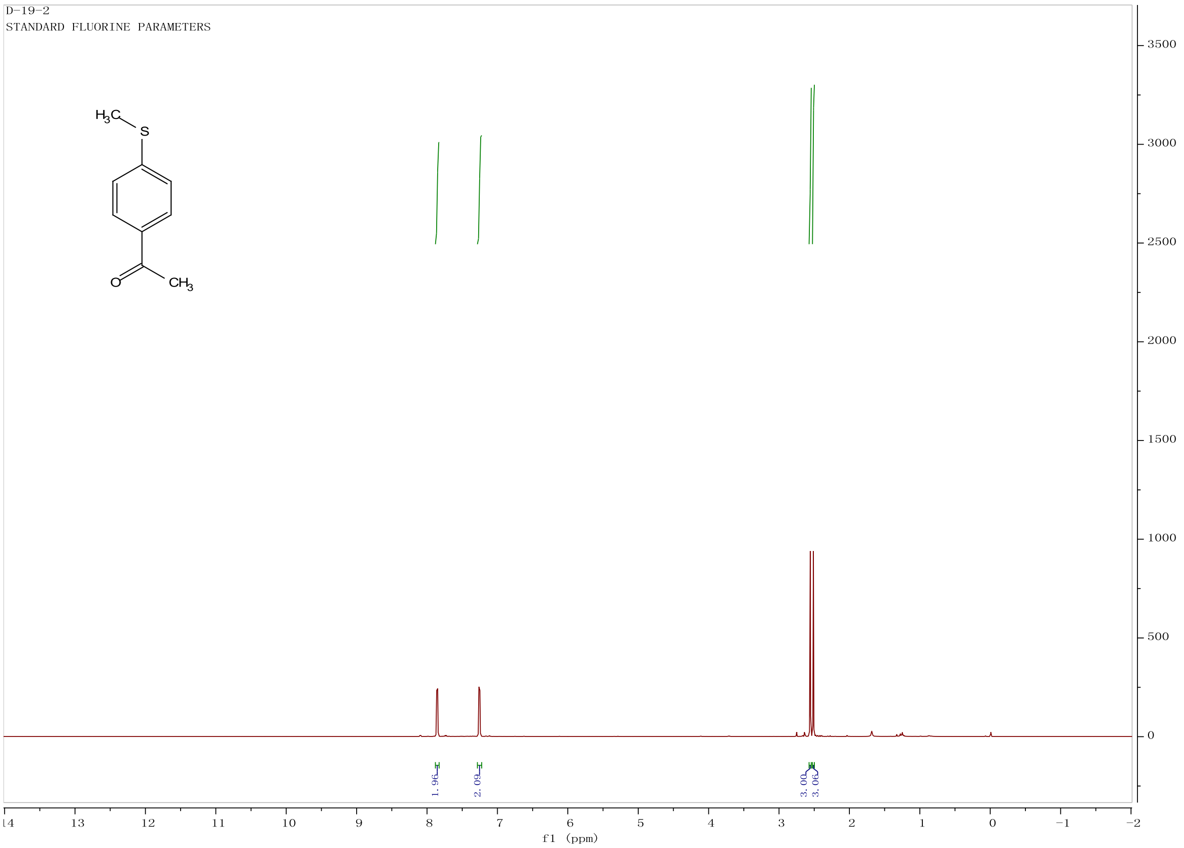
D-19-2
STANDARD FLUORINE PARAMETERS
1.96
2.09
3.00
3.06
f1 (ppm)

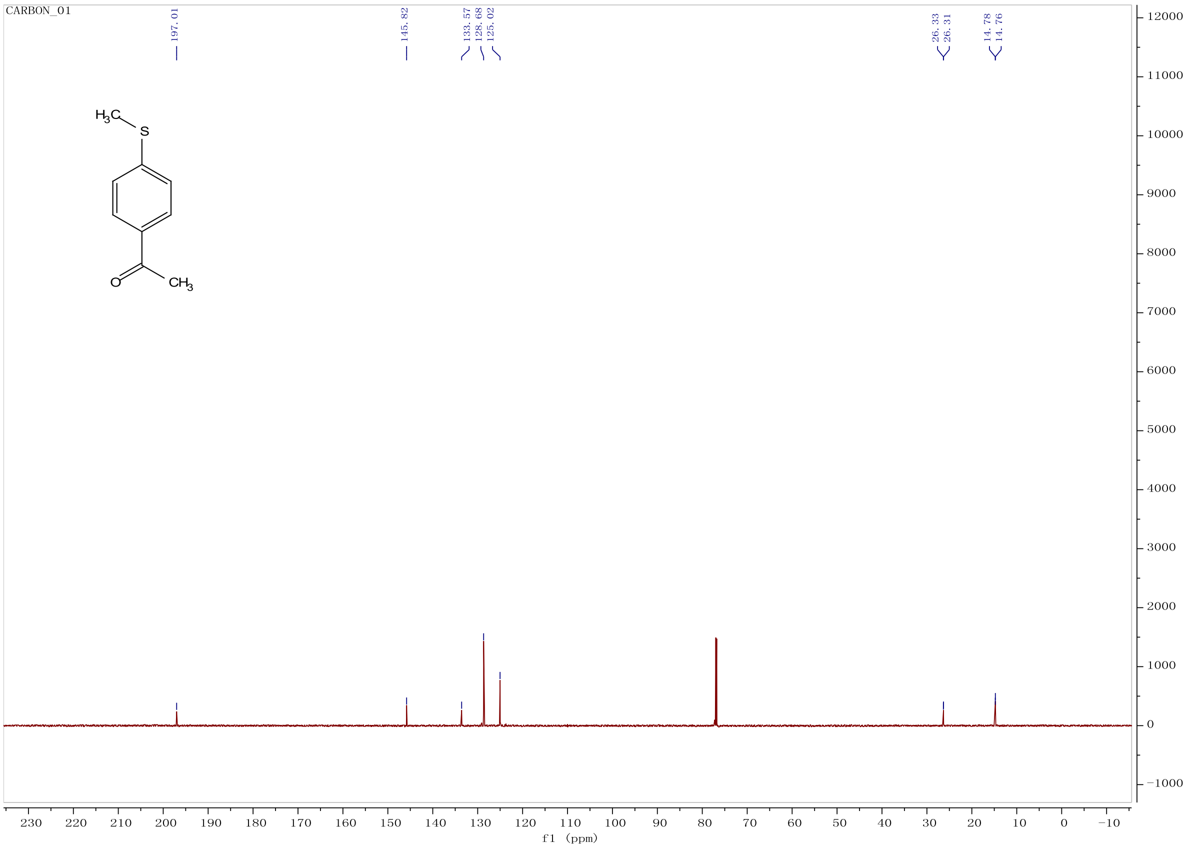
CARBON_01
197.01
145.82
133.57
128.68
125.02
26.33
26.31
14.78
14.76
f1 (ppm)

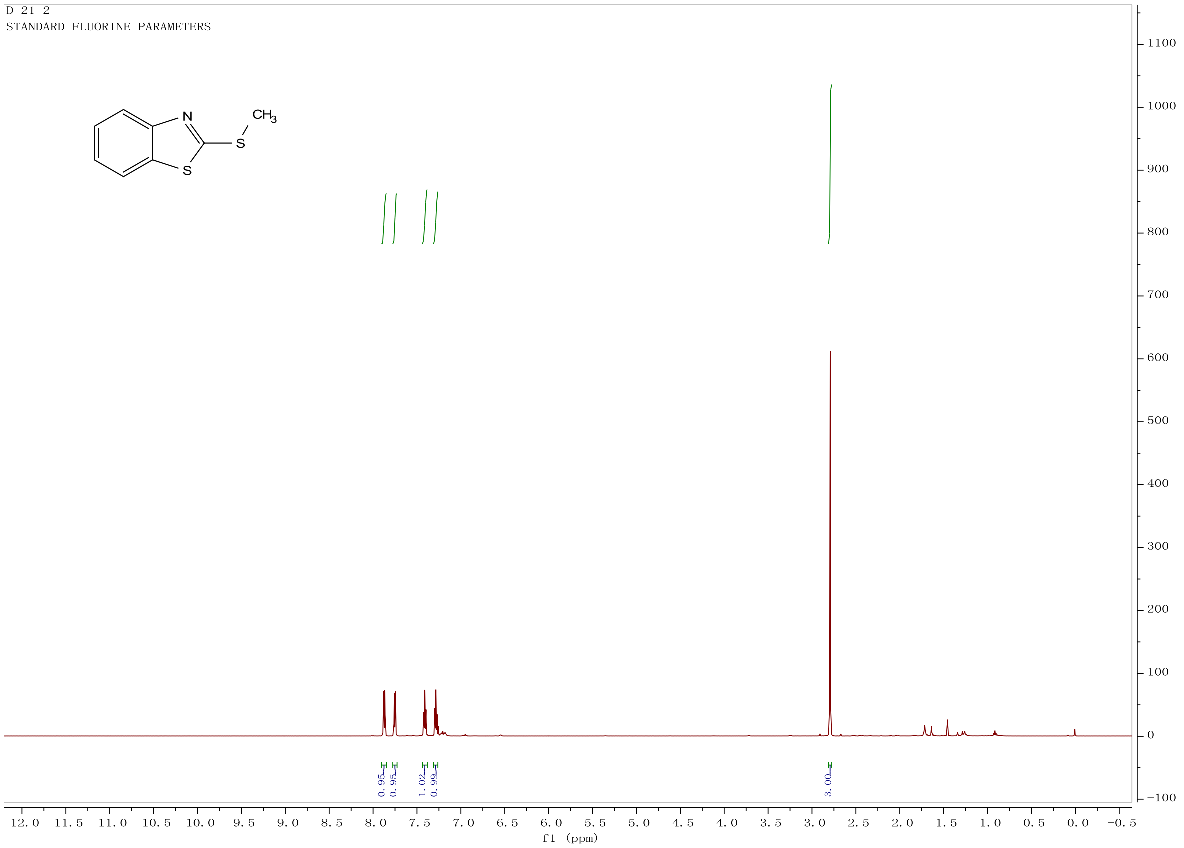
D-21-2
STANDARD FLUORINE PARAMETERS
N
S
S
CH3
0.95
0.95
1.02
0.99
3.00
f1 (ppm)

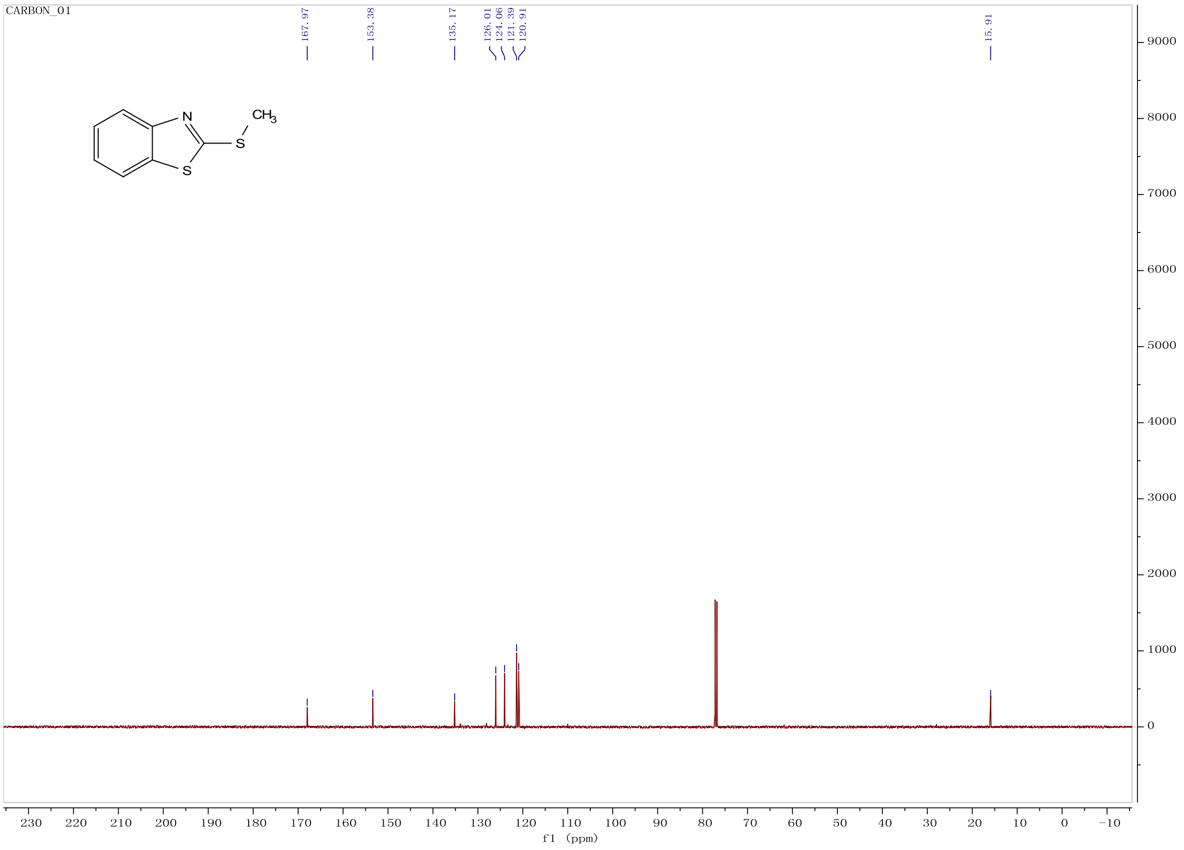
CARBON_01
167.97
153.38
135.17
126.01
124.06
121.39
120.91
15.91
N
S
S
CH3
f1 (ppm)

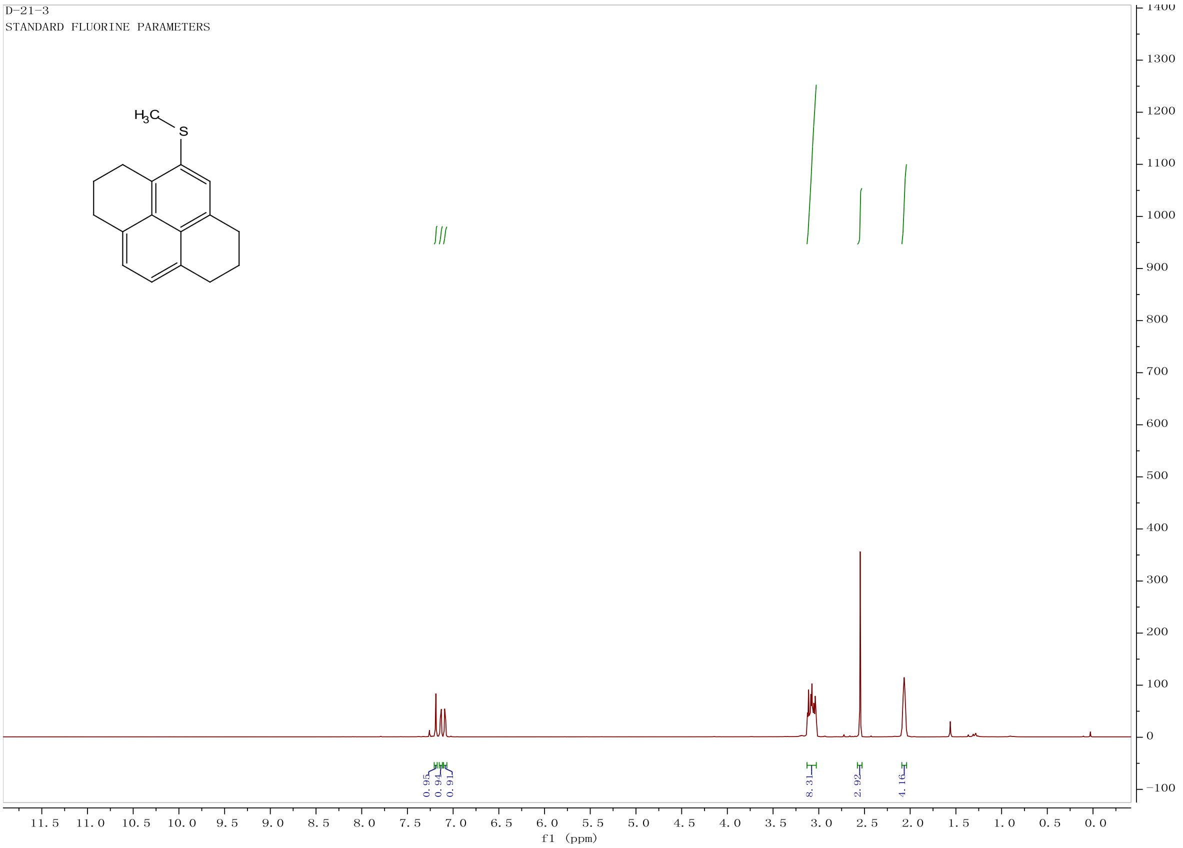
D-21-3
STANDARD FLUORINE PARAMETERS
H3C
S
0.95
0.94
0.91
8.31
2.92
4.16
f1 (ppm)

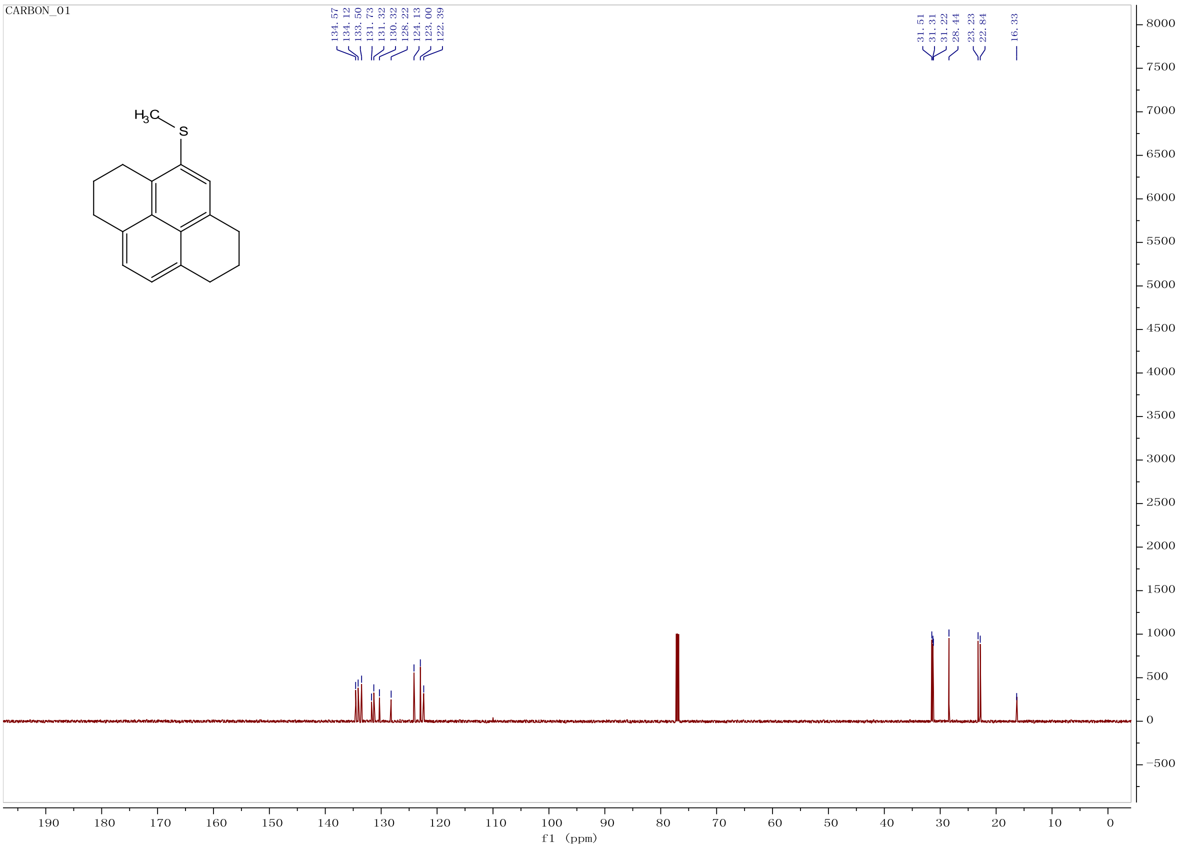
CARBON_01
134.57
134.12
133.50
131.73
131.32
130.32
128.22
124.13
123.00
122.39
31.51
31.31
31.22
28.44
23.23
22.84
16.33
H3C
S
f1 (ppm)

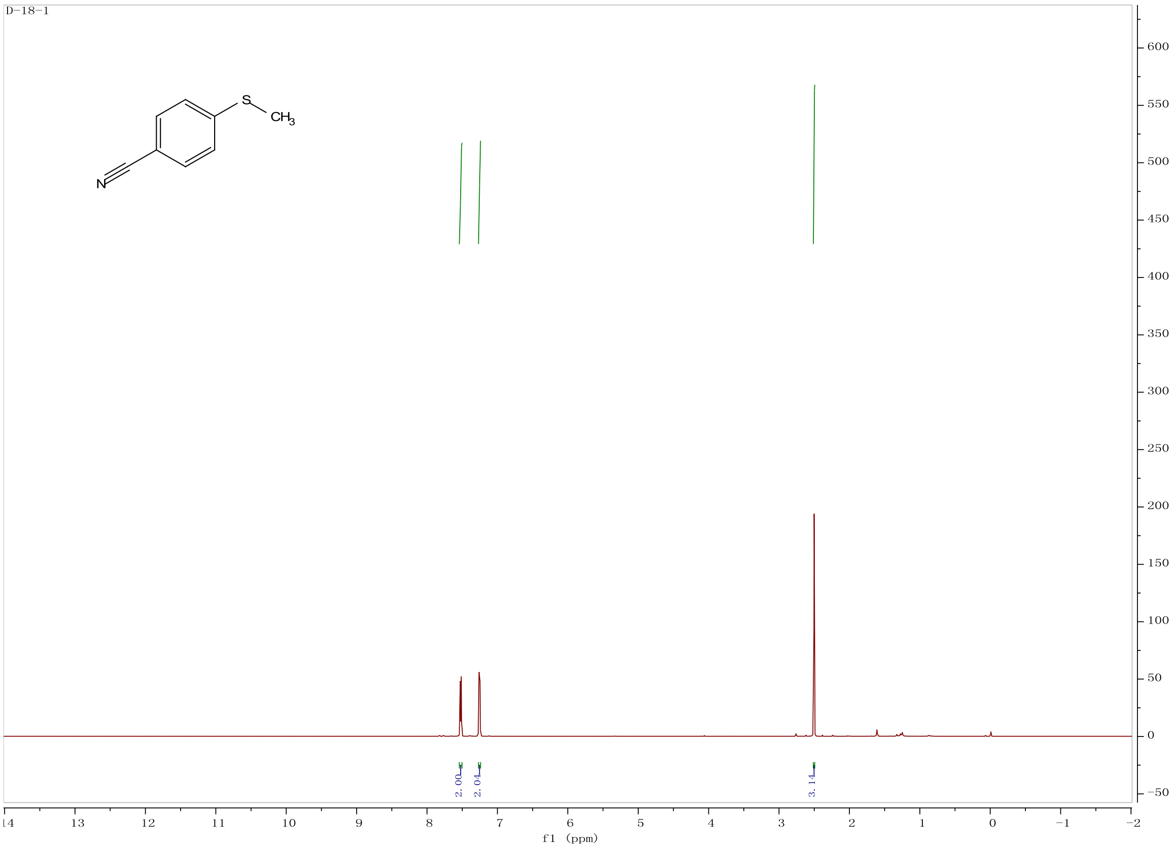
D-18-1
S
CH3
N
2.00
2.04
3.14
14 13 12 11 10 9 8 7 6 5 4 3 2 1 0 −1 −2
f1 (ppm)
600 550 500 450 400 350 300 250 200 150 100 50 0 −50

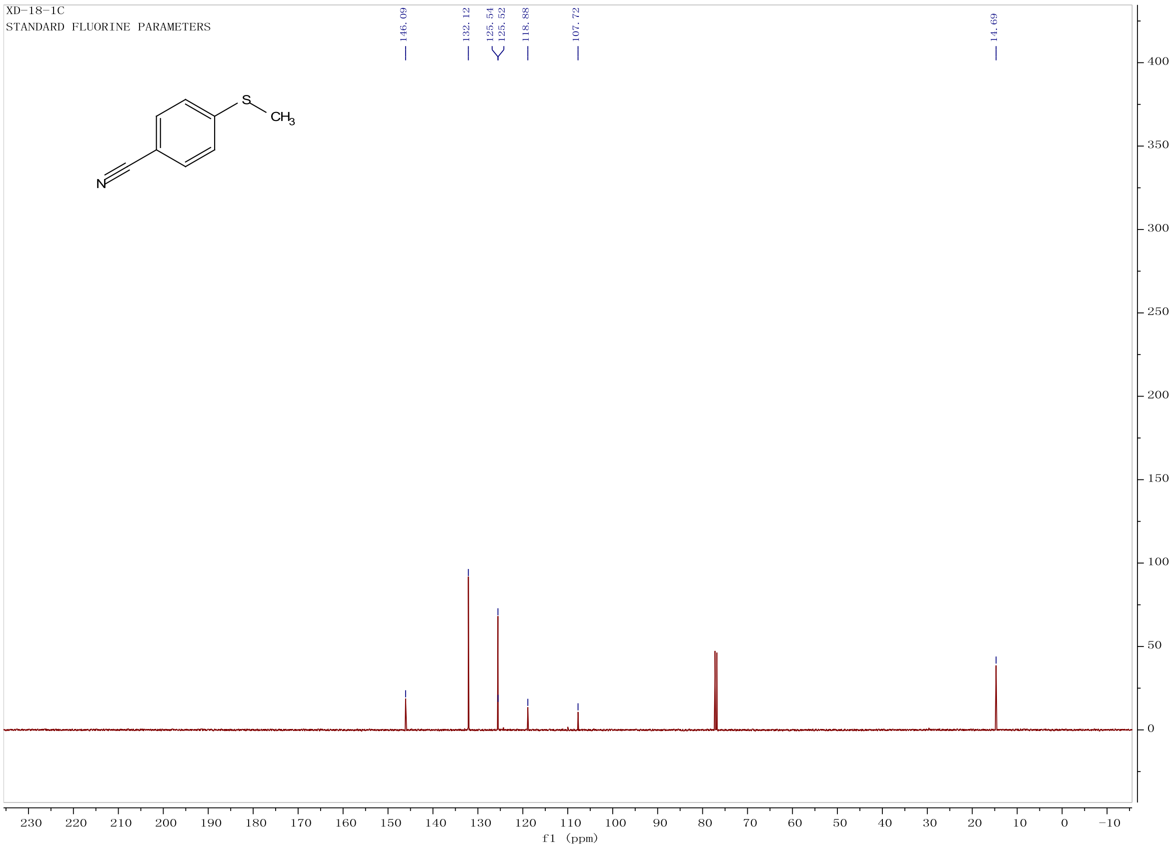
XD-18-1C
STANDARD FLUORINE PARAMETERS
146.09
132.12
125.54
125.52
118.88
107.72
14.69
S
CH3
N
230 220 210 200 190 180 170 160 150 140 130 120 110 100 90 80 70 60 50 40 30 20 10 0 −10
f1 (ppm)
400 350 300 250 200 150 100 50 0

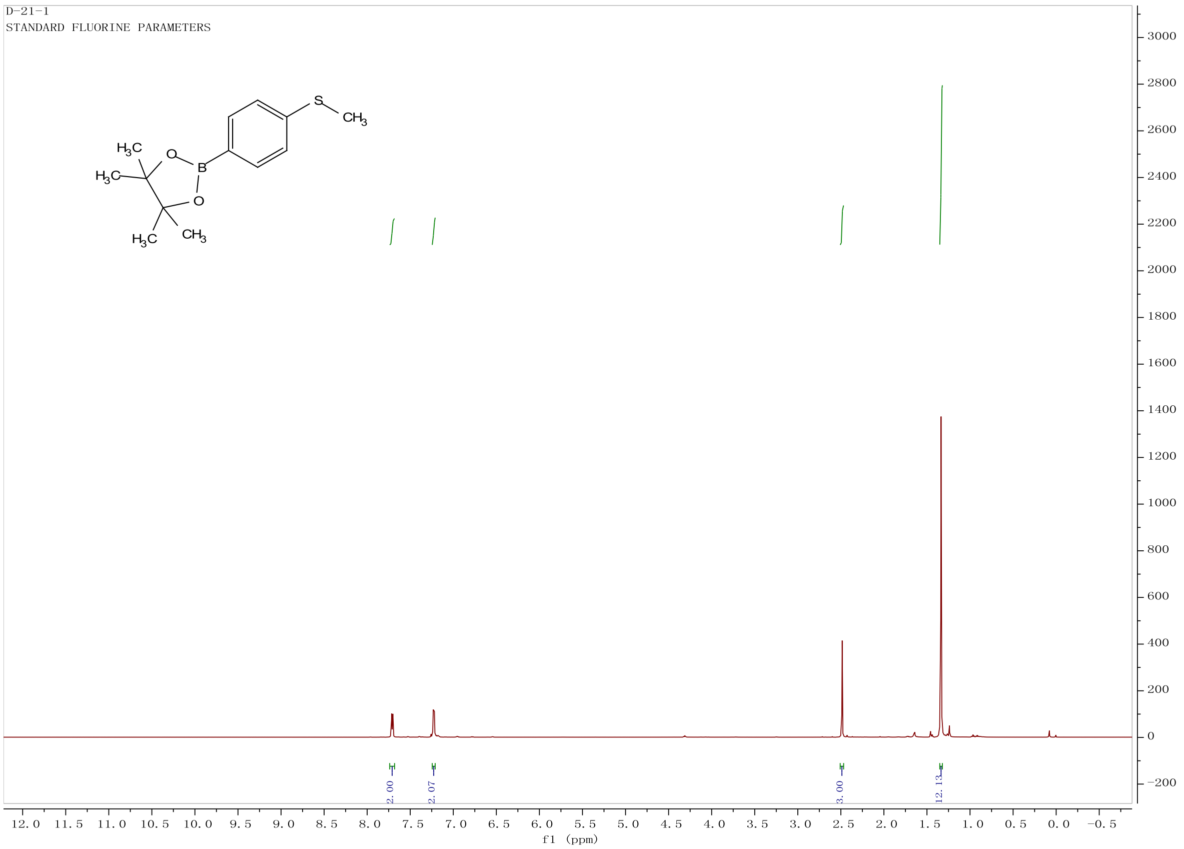
D-21-1
STANDARD FLUORINE PARAMETERS
2.00
2.07
3.00
12.13
f1 (ppm)

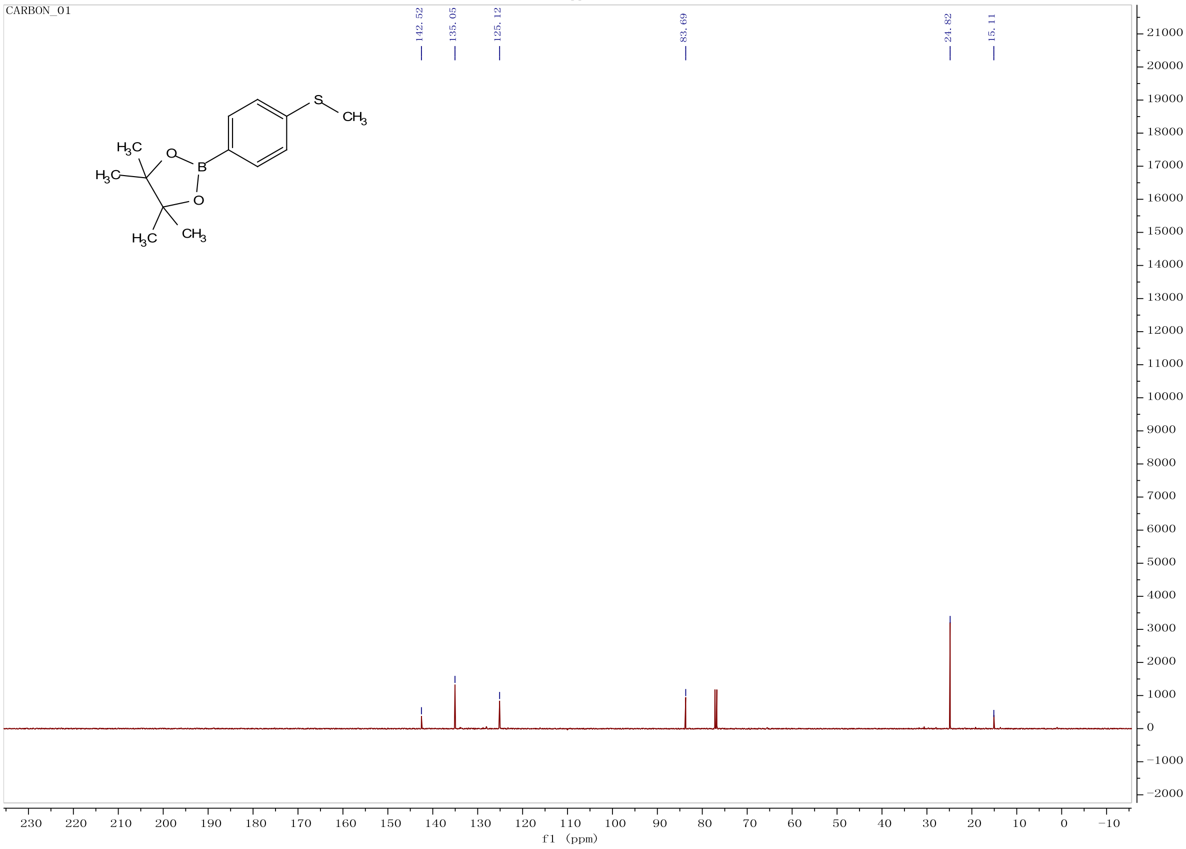
CARBON_01
142.52
135.05
125.12
83.69
24.82
15.11
f1 (ppm)

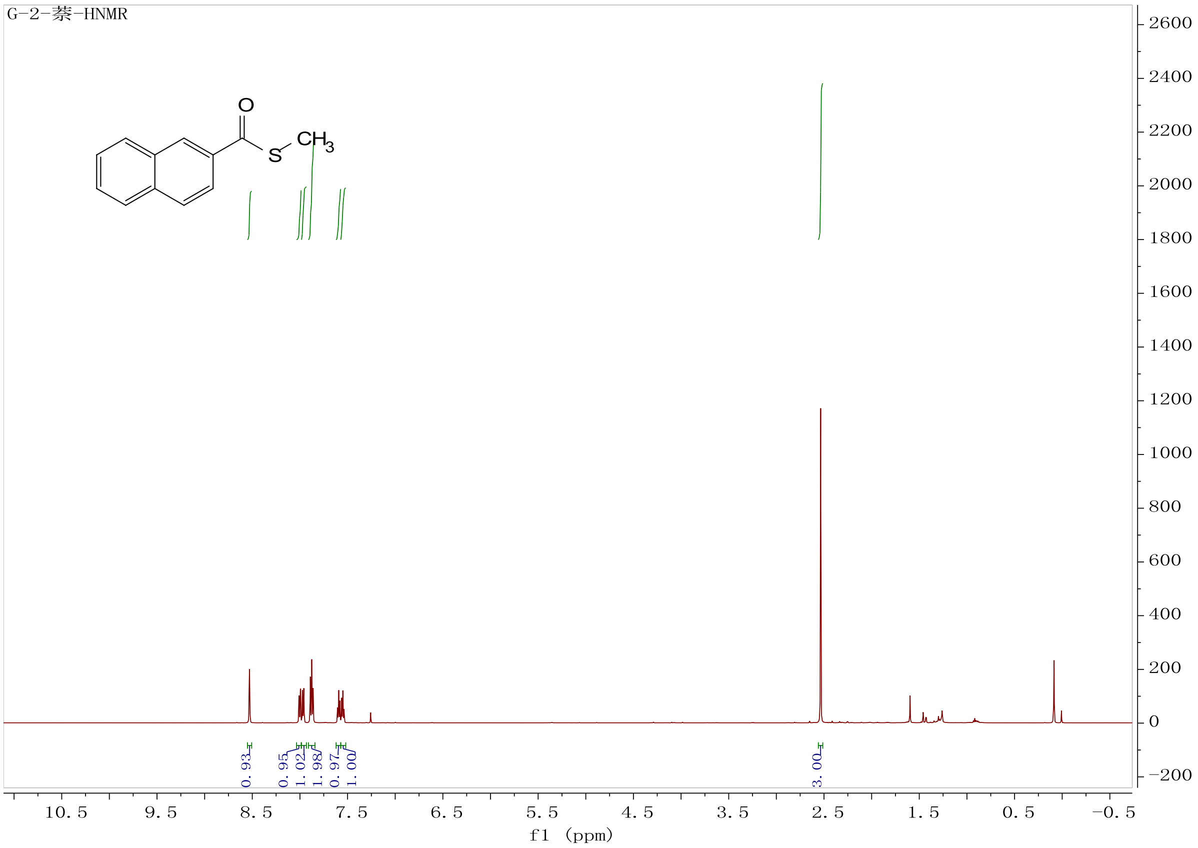
G-2-萘-HNMR
O
S
CH3
0.93
0.95
1.02
1.98
0.97
1.00
3.00
10.5 9.5 8.5 7.5 6.5 5.5 4.5 3.5 2.5 1.5 0.5 −0.5
f1 (ppm)
2600 2400 2200 2000 1800 1600 1400 1200 1000 800 600 400 200 0 −200

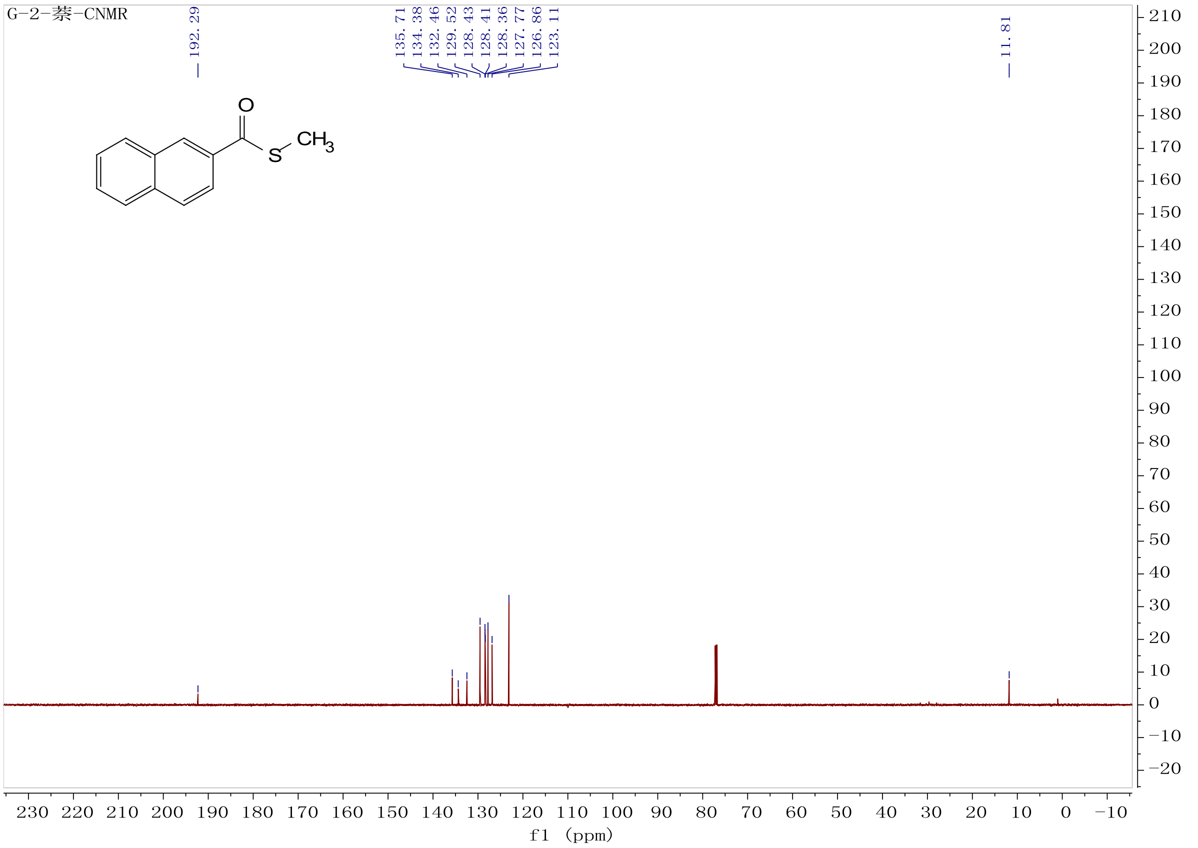
G-2-萘-CNMR
192.29
135.71
134.38
132.46
129.52
128.43
128.41
128.36
127.77
126.86
123.11
11.81
O
S
CH3
230 220 210 200 190 180 170 160 150 140 130 120 110 100 90 80 70 60 50 40 30 20 10 0 −10
f1 (ppm)
210 200 190 180 170 160 150 140 130 120 110 100 90 80 70 60 50 40 30 20 10 0 −10 −20

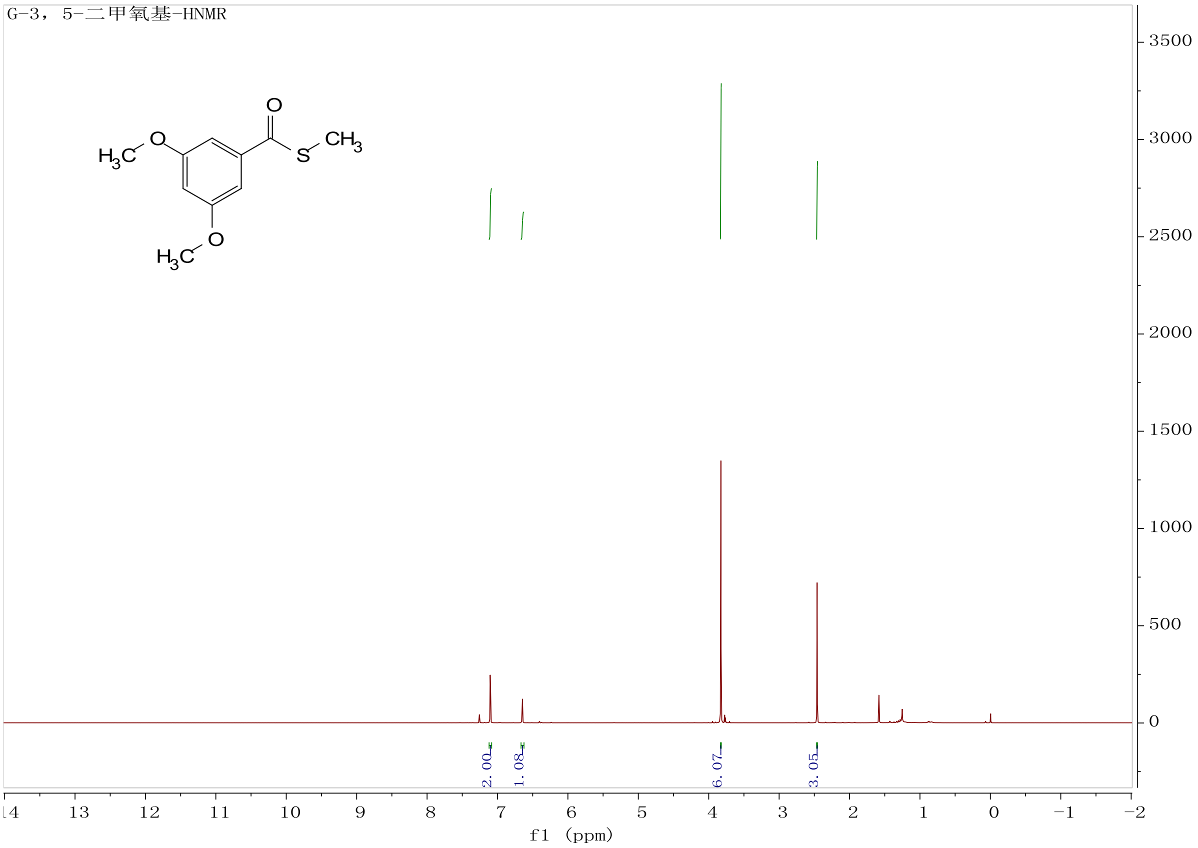
G-3，5-二甲氧基-HNMR
O
H_3C O
S CH_3
H_3C O
2.00
1.08
6.07
3.05
f1 (ppm)

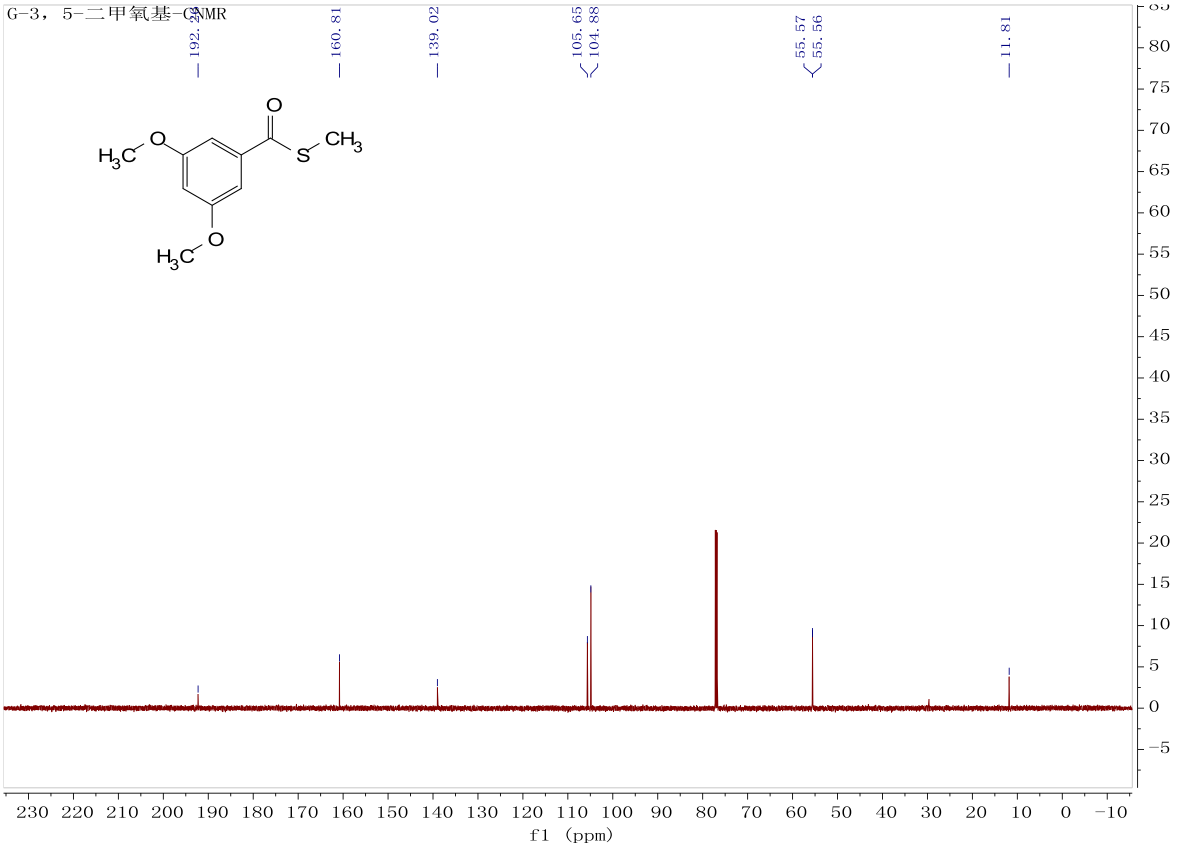
G-3，5-二甲氧基-CNMR
192.2
160.81
139.02
105.65
104.88
55.57
55.56
11.81
O
H_3C O
S CH_3
H_3C O
f1 (ppm)

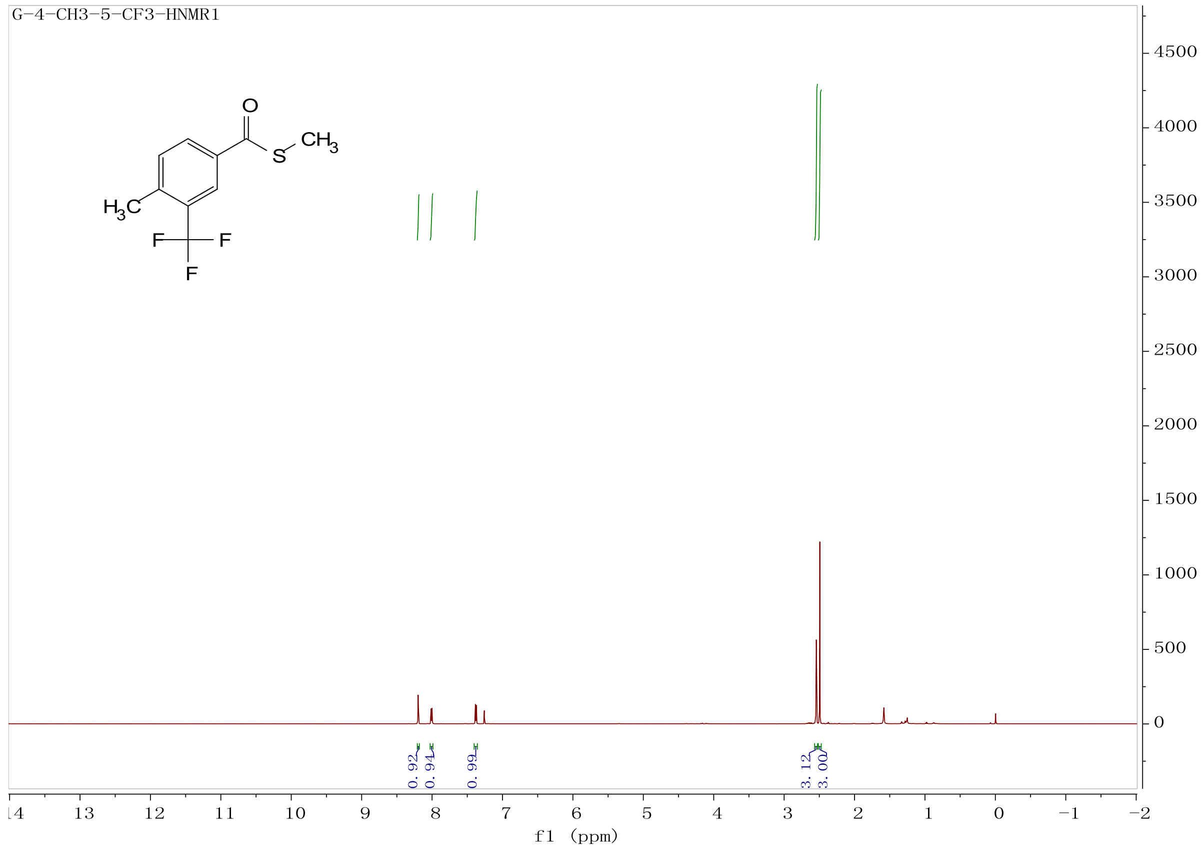
G-4-CH3-5-CF3-HNMR1
O
S
CH3
H3C
F
F
F
0.92
0.94
0.99
3.12
3.00
f1 (ppm)

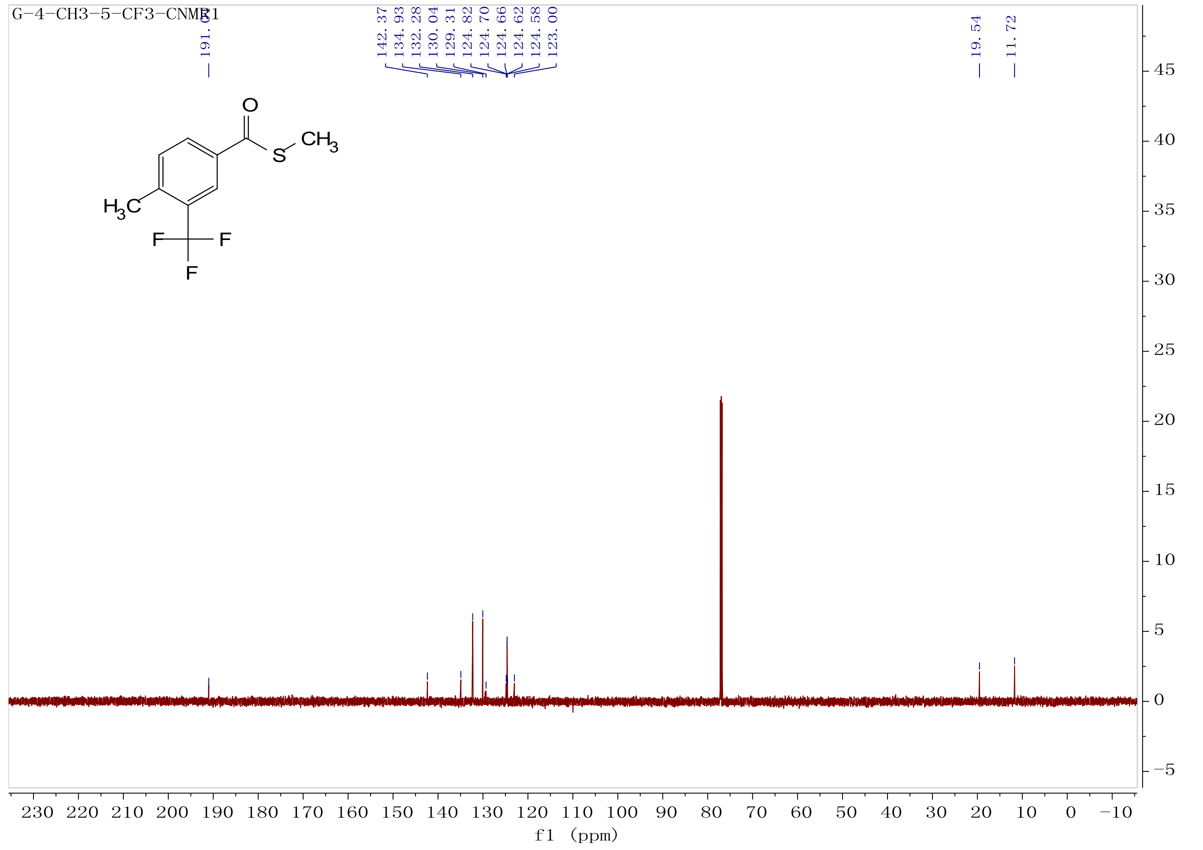
G-4-CH3-5-CF3-CNMR1
142.37
134.93
132.28
130.04
129.31
124.82
124.70
124.66
124.62
124.58
123.00
19.54
11.72
O
S
CH3
H3C
F
F
F
f1 (ppm)

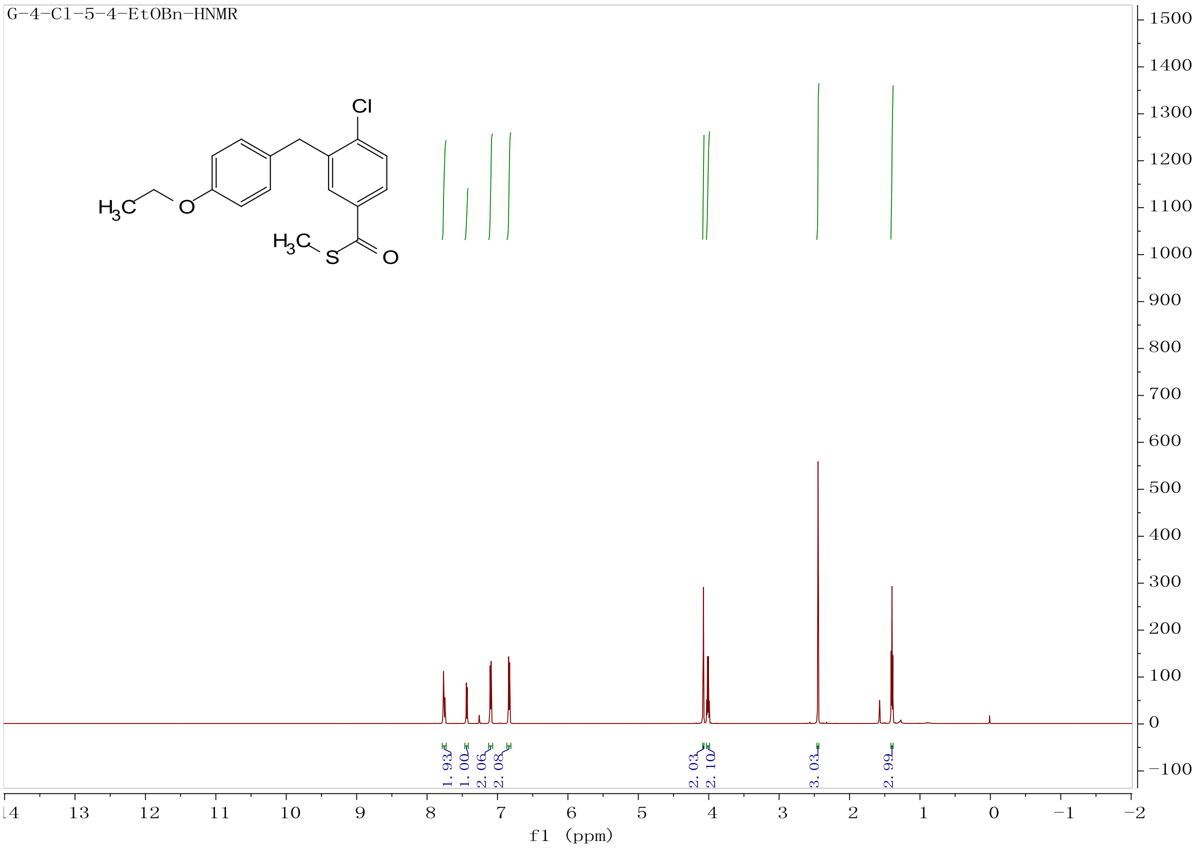
G-4-Cl-5-4-EtOBn-HNMR
Cl
H_3C
O
H_3C
S
O
1.93
1.00
2.06
2.08
2.03
2.10
3.03
2.99
f1 (ppm)

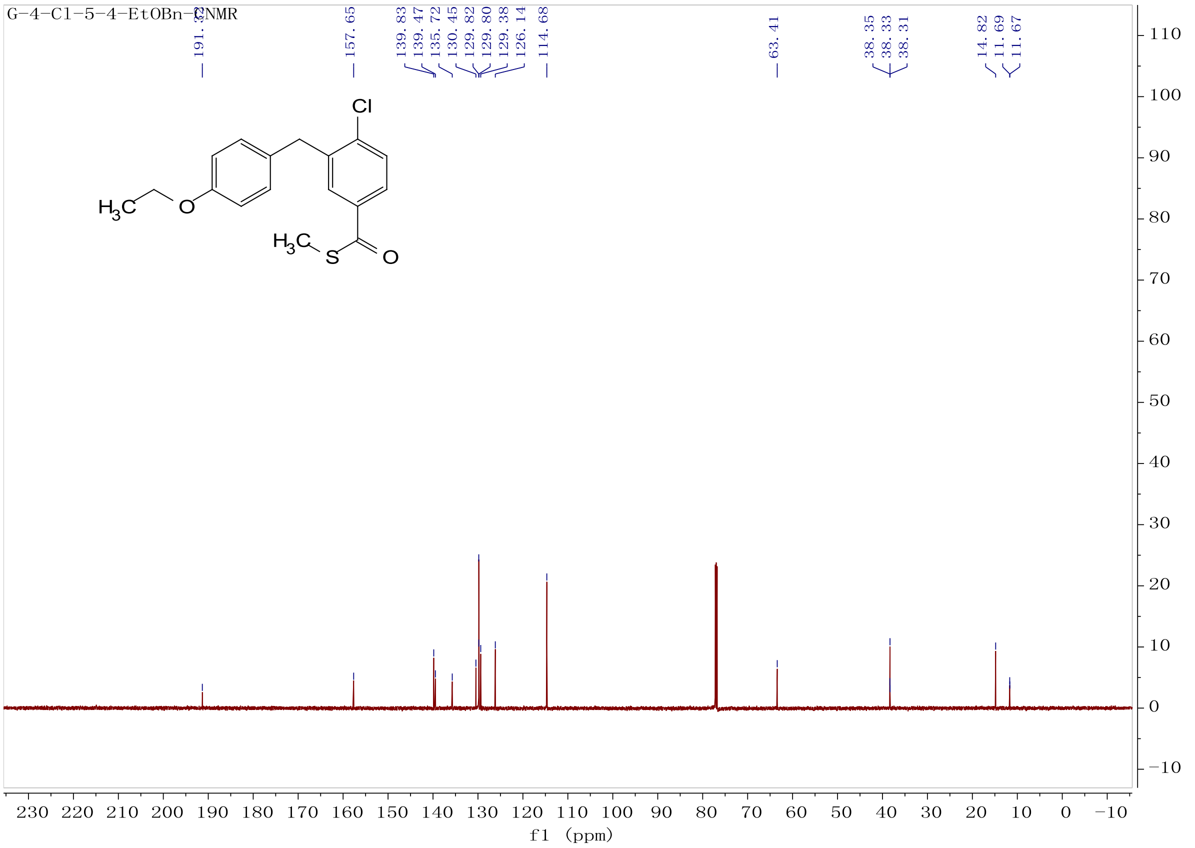
G-4-Cl-5-4-EtOBn-CNMR
191.32
157.65
139.83
139.47
135.72
130.45
129.82
129.80
129.38
126.14
114.68
63.41
38.35
38.33
38.31
14.82
11.69
11.67
Cl
H_3C
O
H_3C
S
O
f1 (ppm)

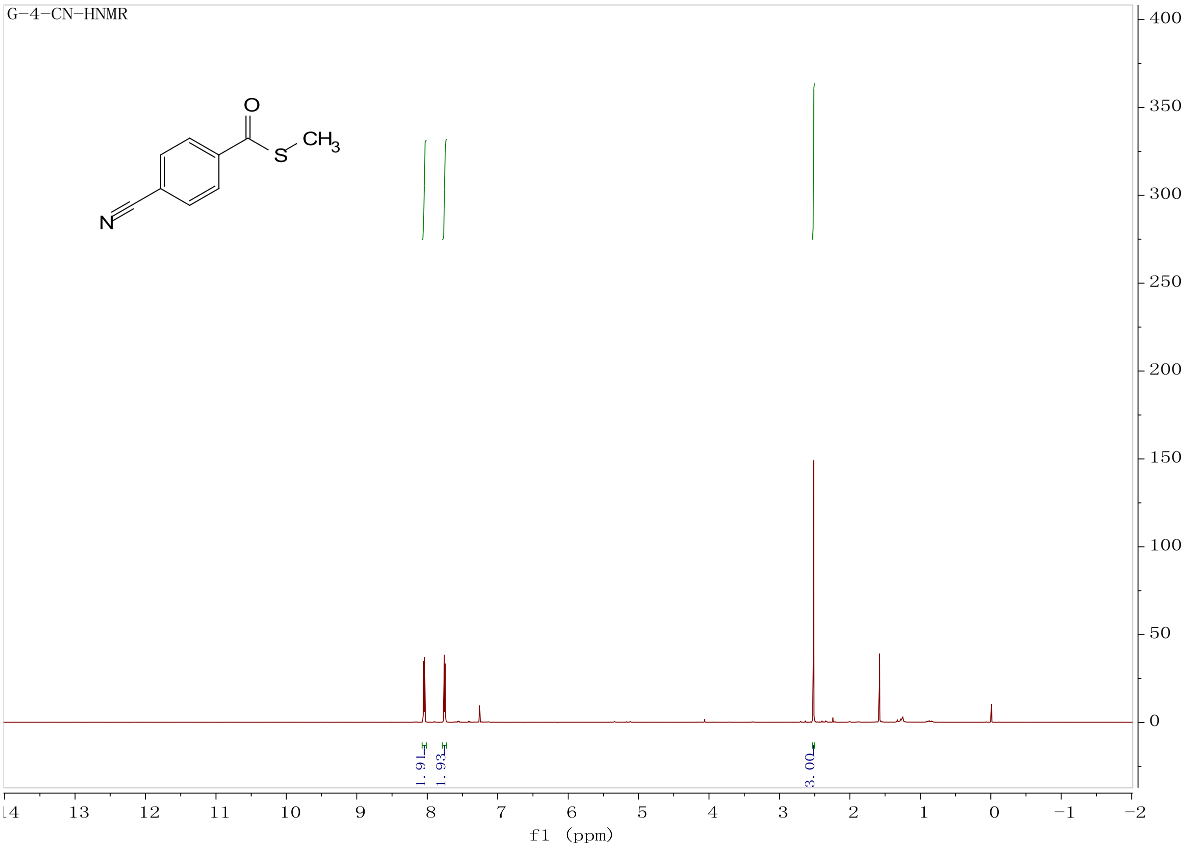
G-4-CN-HNMR
O
S
CH3
N
1.91
1.93
3.00
f1 (ppm)

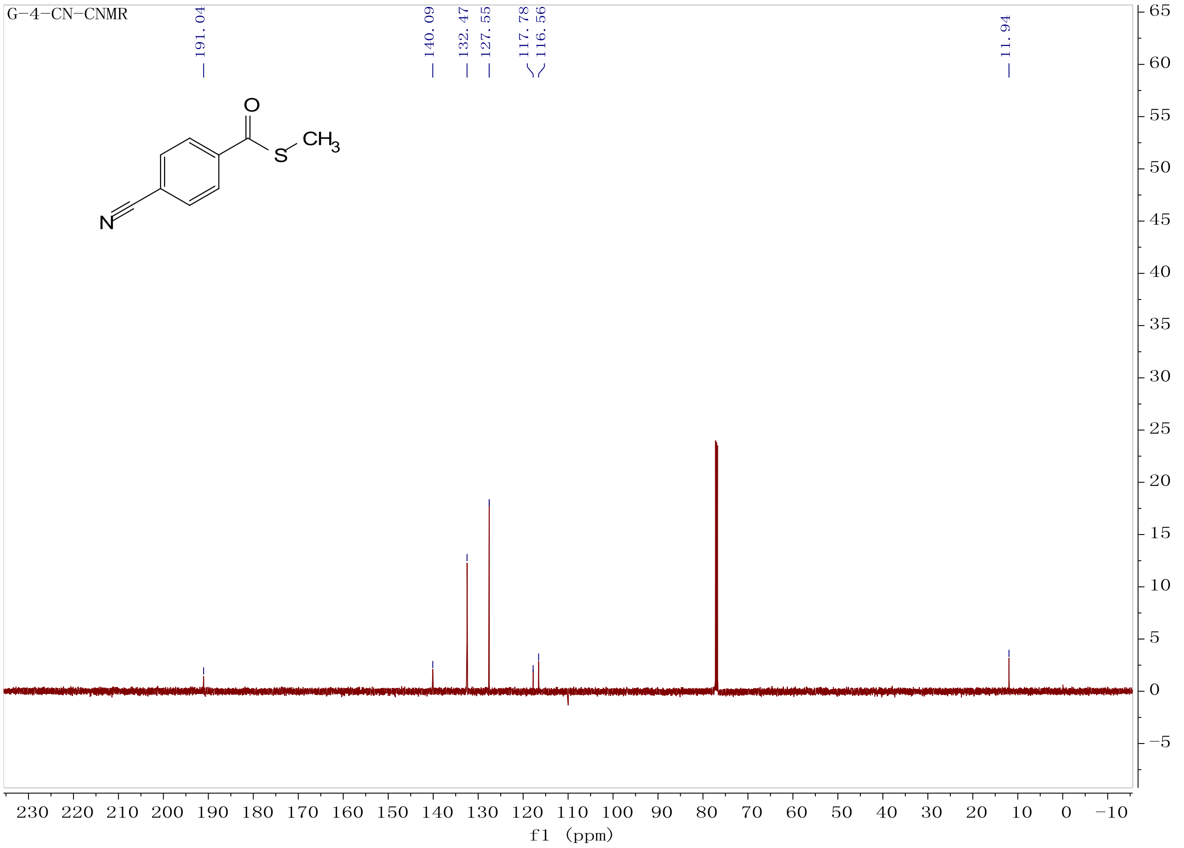
G-4-CN-CNMR
191.04
140.09
132.47
127.55
117.78
116.56
11.94
O
S
CH3
N
f1 (ppm)

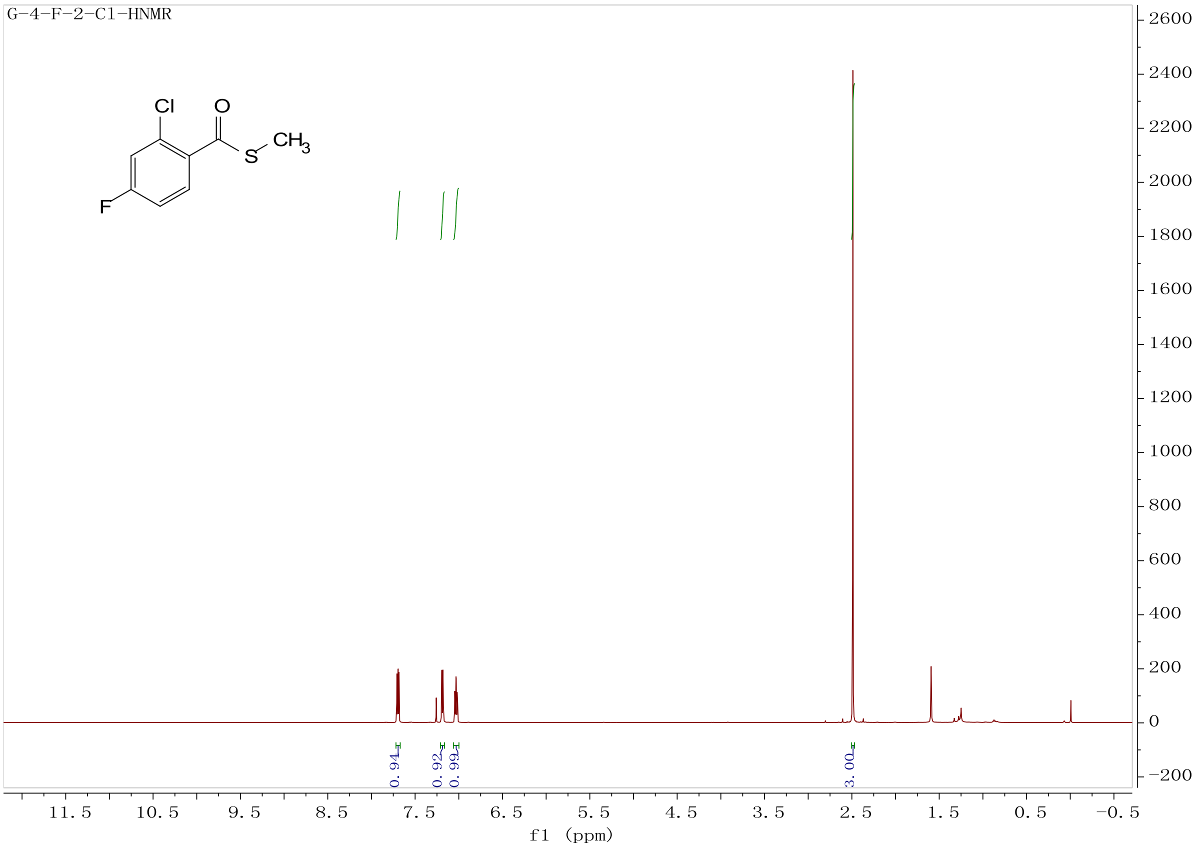
G-4-F-2-Cl-HNMR
Cl
O
S
CH3
F
0.94
0.92
0.99
3.00
11.5 10.5 9.5 8.5 7.5 6.5 5.5 4.5 3.5 2.5 1.5 0.5 −0.5
f1 (ppm)
2600 2400 2200 2000 1800 1600 1400 1200 1000 800 600 400 200 0 −200

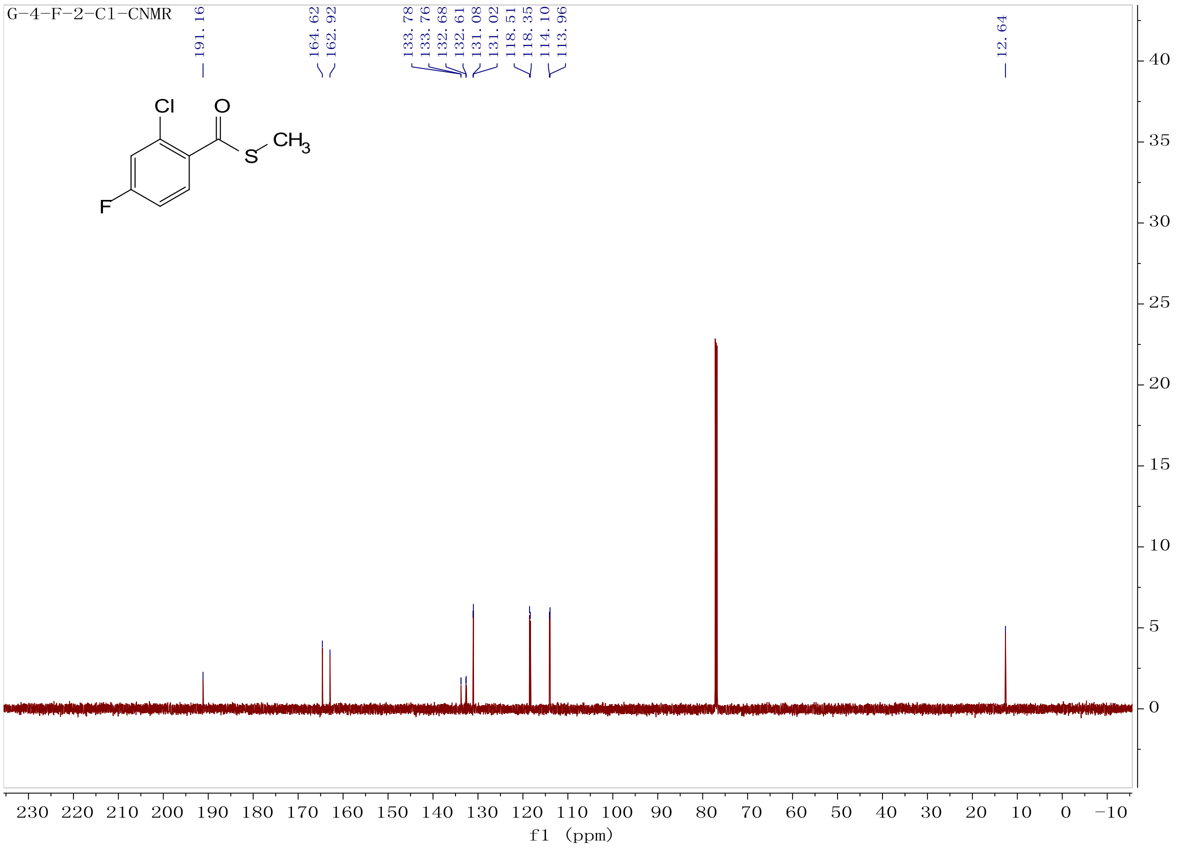
G-4-F-2-Cl-CNMR
191.16
164.62
162.92
133.78
133.76
132.68
132.61
131.08
131.02
118.51
118.35
114.10
113.96
12.64
Cl
O
S
CH3
F
230 220 210 200 190 180 170 160 150 140 130 120 110 100 90 80 70 60 50 40 30 20 10 0 −10
f1 (ppm)
40 35 30 25 20 15 10 5 0

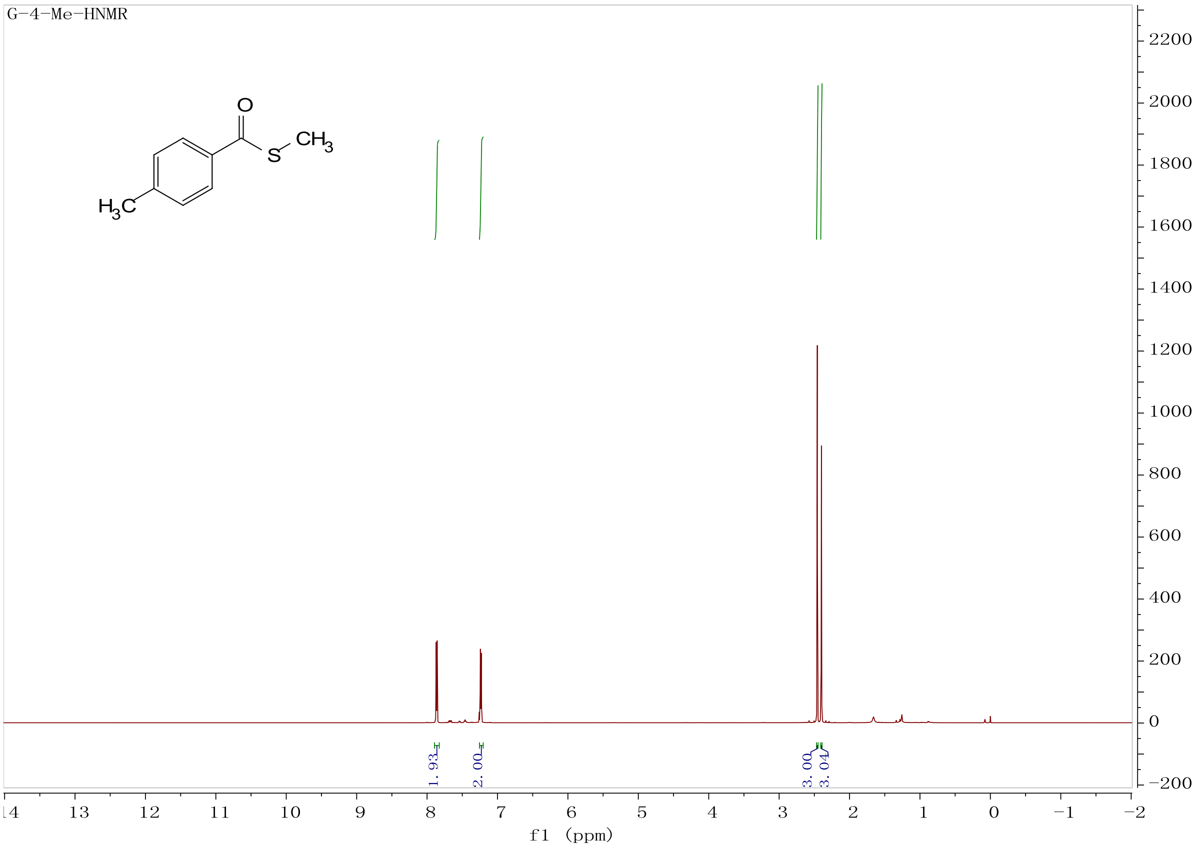
G-4-Me-HNMR
O
CH3
S
H3C
1.93
2.00
3.00
3.04
f1 (ppm)

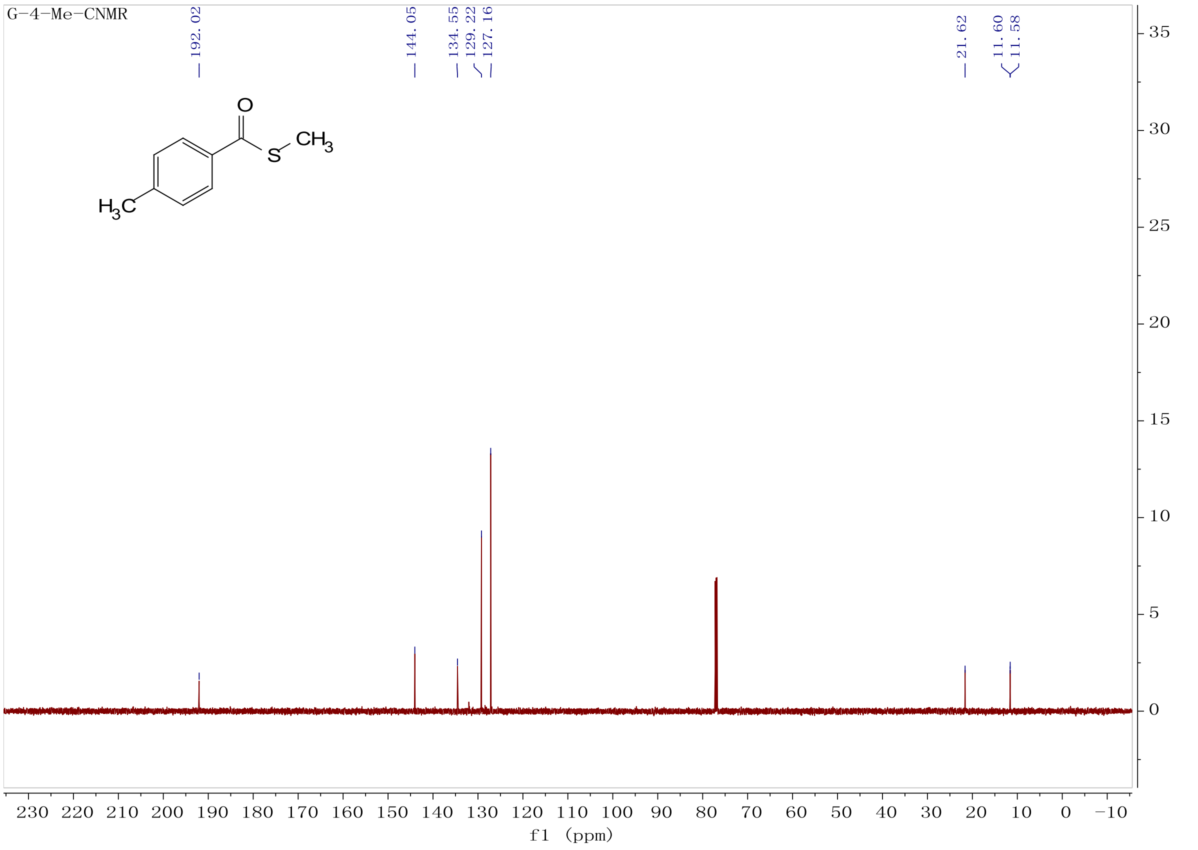
G-4-Me-CNMR
192.02
144.05
134.55
129.22
127.16
21.62
11.60
11.58
O
CH3
S
H3C
f1 (ppm)

G-4-N，N-二甲基-HNMR

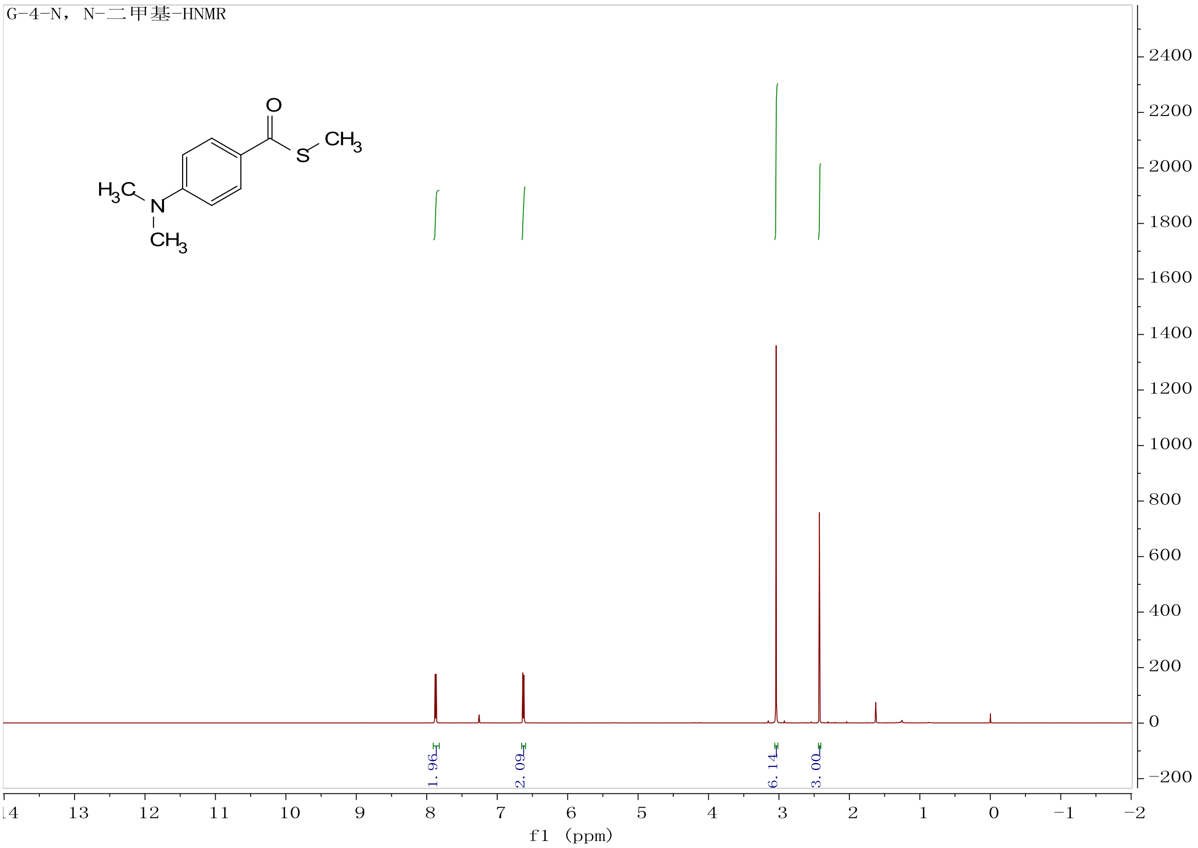

G-4-N，N-二甲基-CNMR

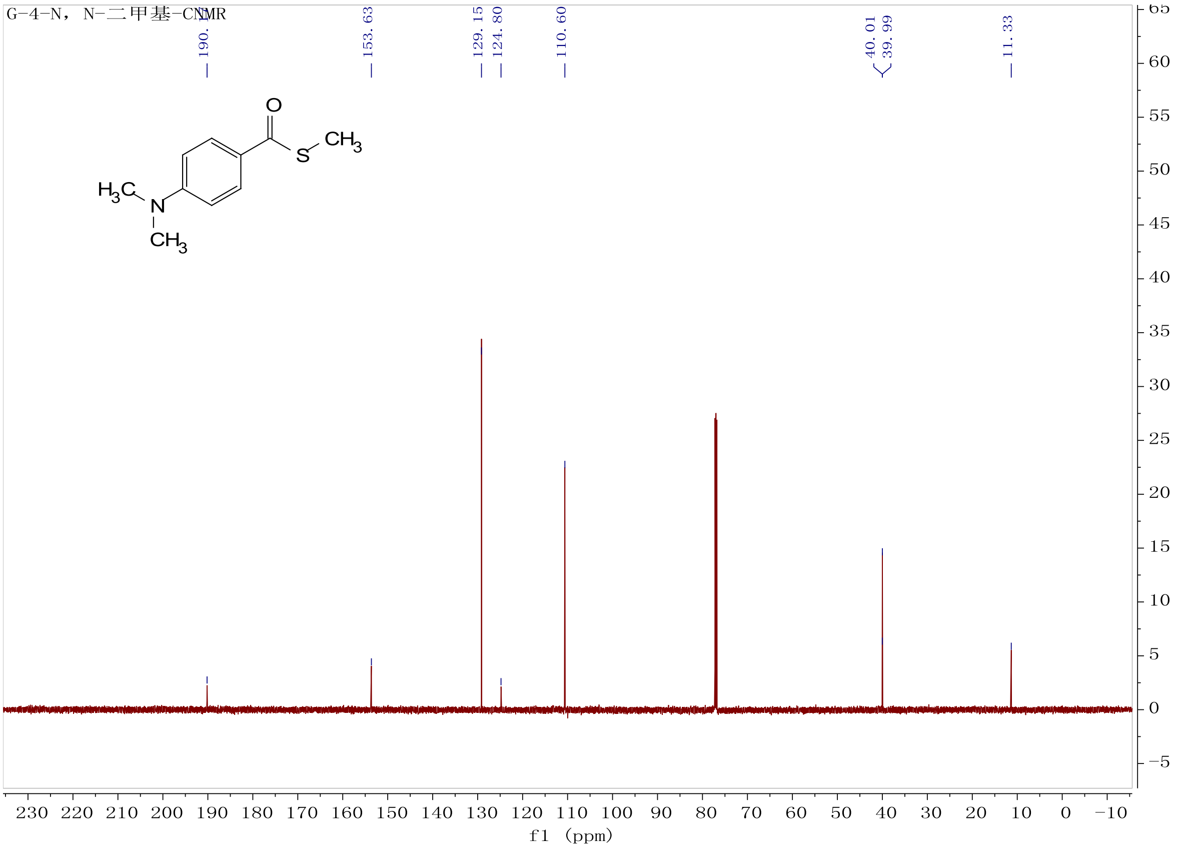

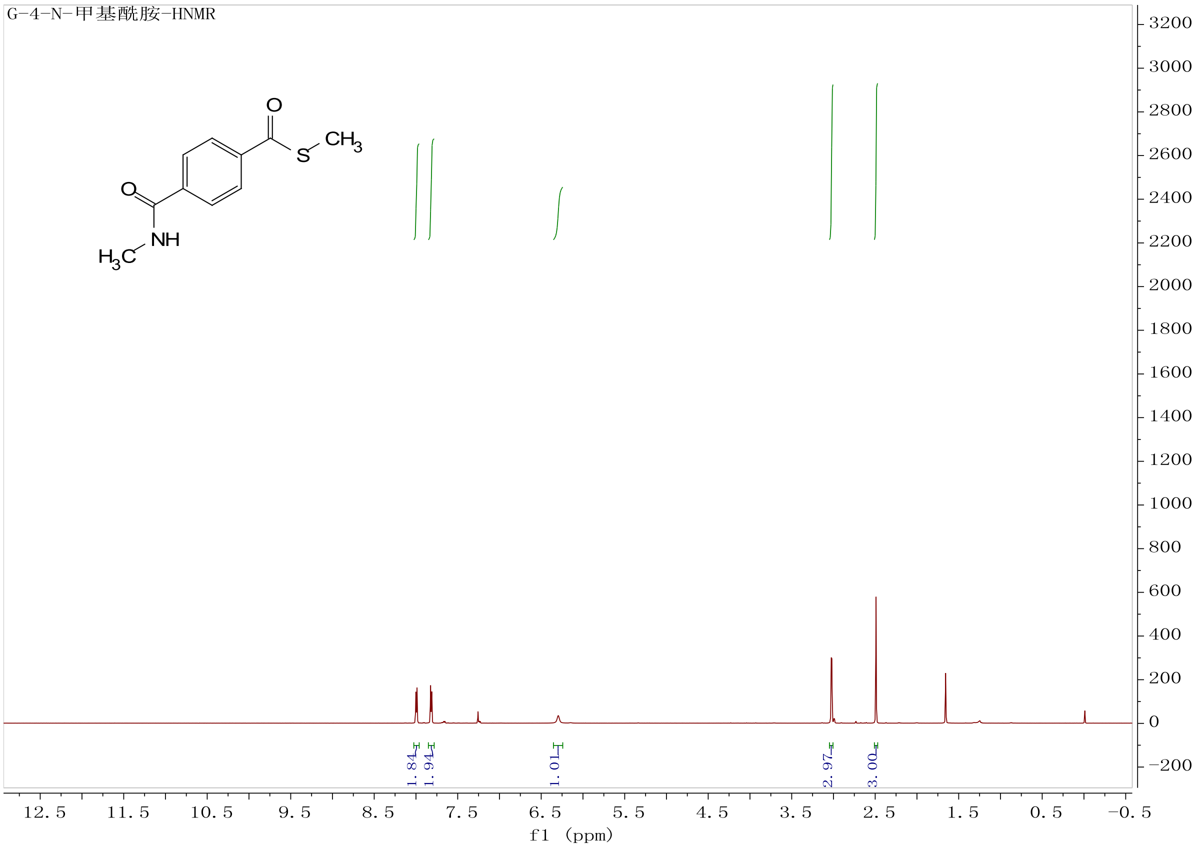
G-4-N-甲基酰胺-HNMR
O
S
CH3
O
NH
H3C
1.84
1.94
1.01
2.97
3.00
f1 (ppm)

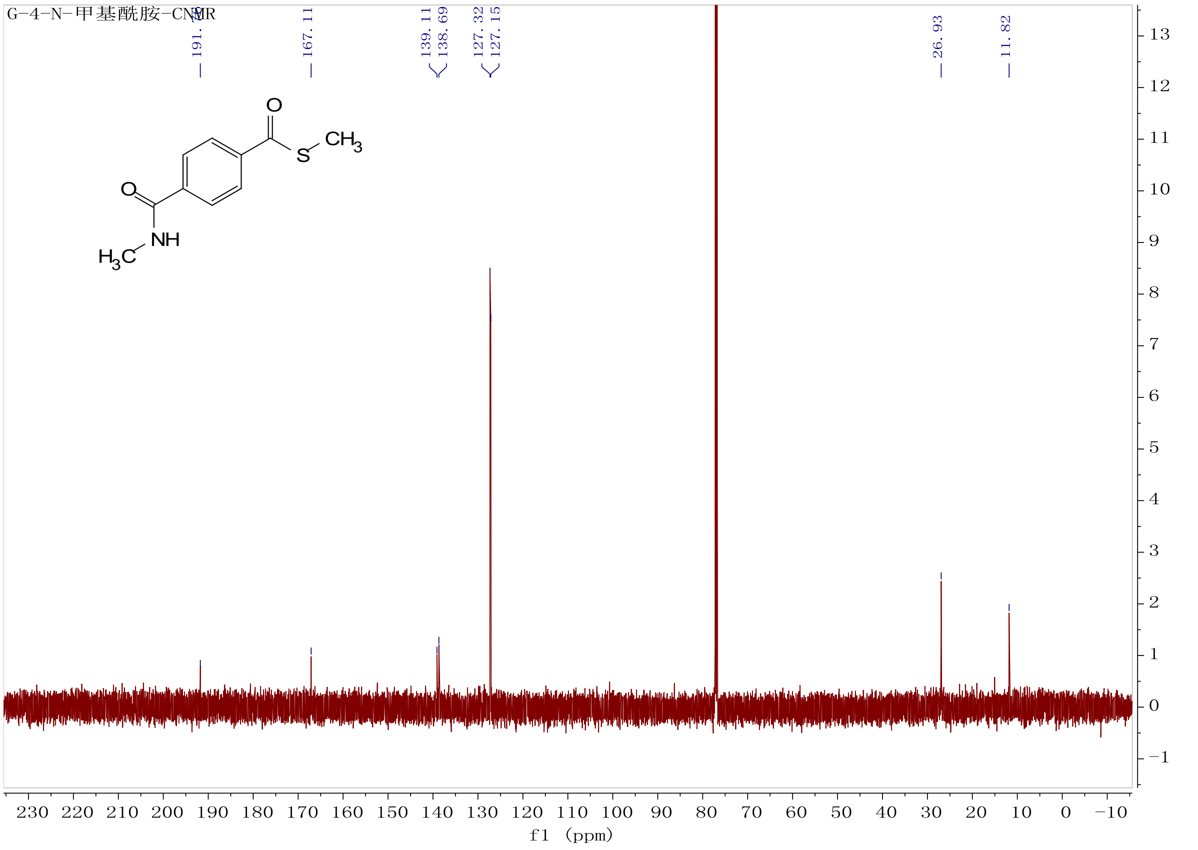
G-4-N-甲基酰胺-CNMR
191.
167.11
139.11
138.69
127.32
127.15
26.93
11.82
O
S
CH3
O
NH
H3C
f1 (ppm)

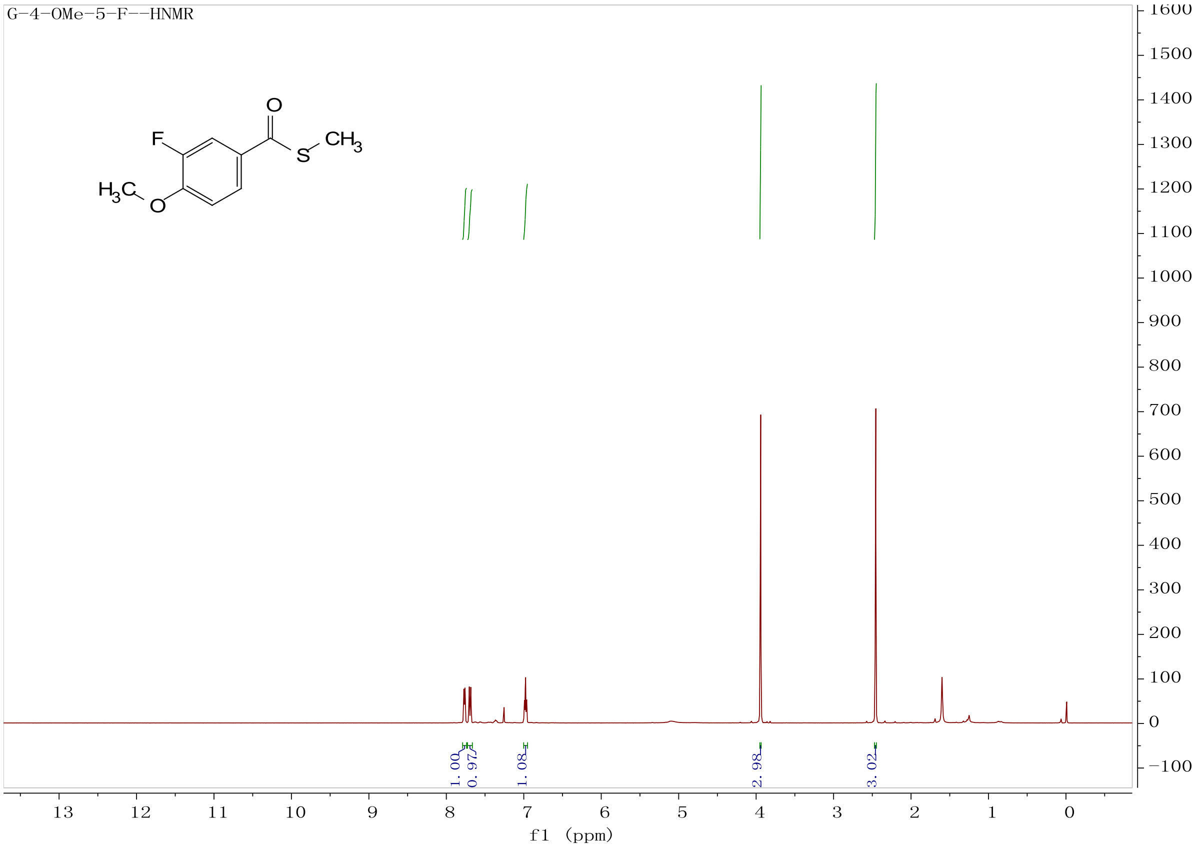
G-4-OMe-5-F--HNMR
F
O
S
CH3
H3C
O
1.00
0.97
1.08
2.98
3.02
f1 (ppm)

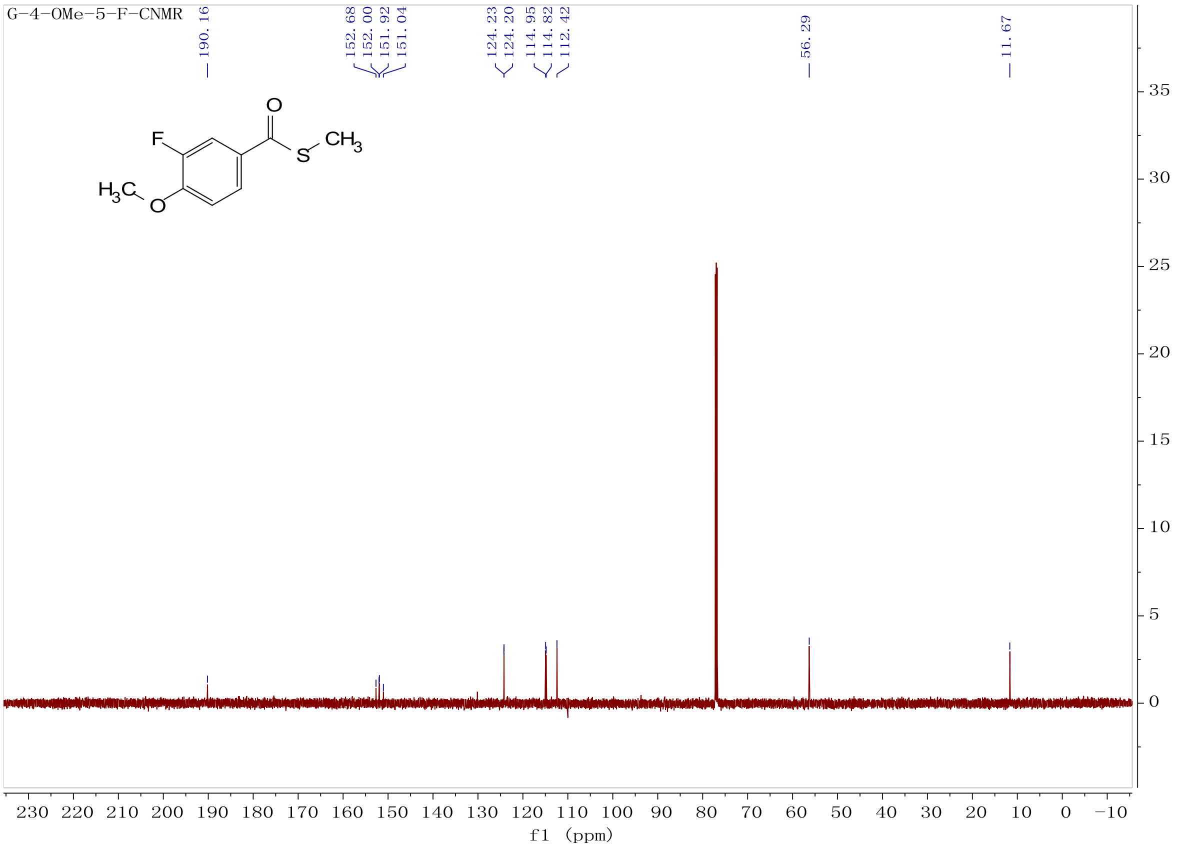
G-4-OMe-5-F-CNMR
190.16
152.68
152.00
151.92
151.04
124.23
124.20
114.95
114.82
112.42
56.29
11.67
F
O
S
CH3
H3C
O
f1 (ppm)

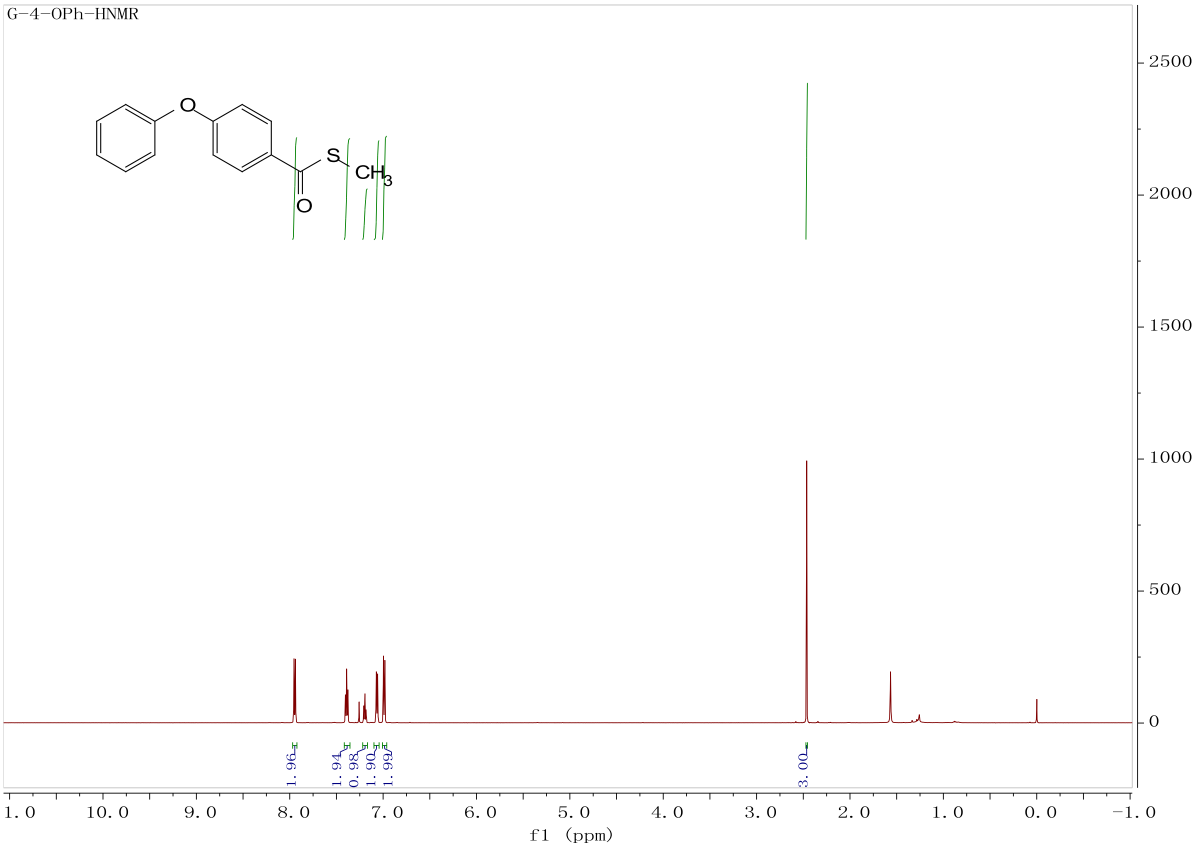
G-4-OPh-HNMR
O
S
CH3
O
1.96
1.94
0.98
1.90
1.99
3.00
f1 (ppm)

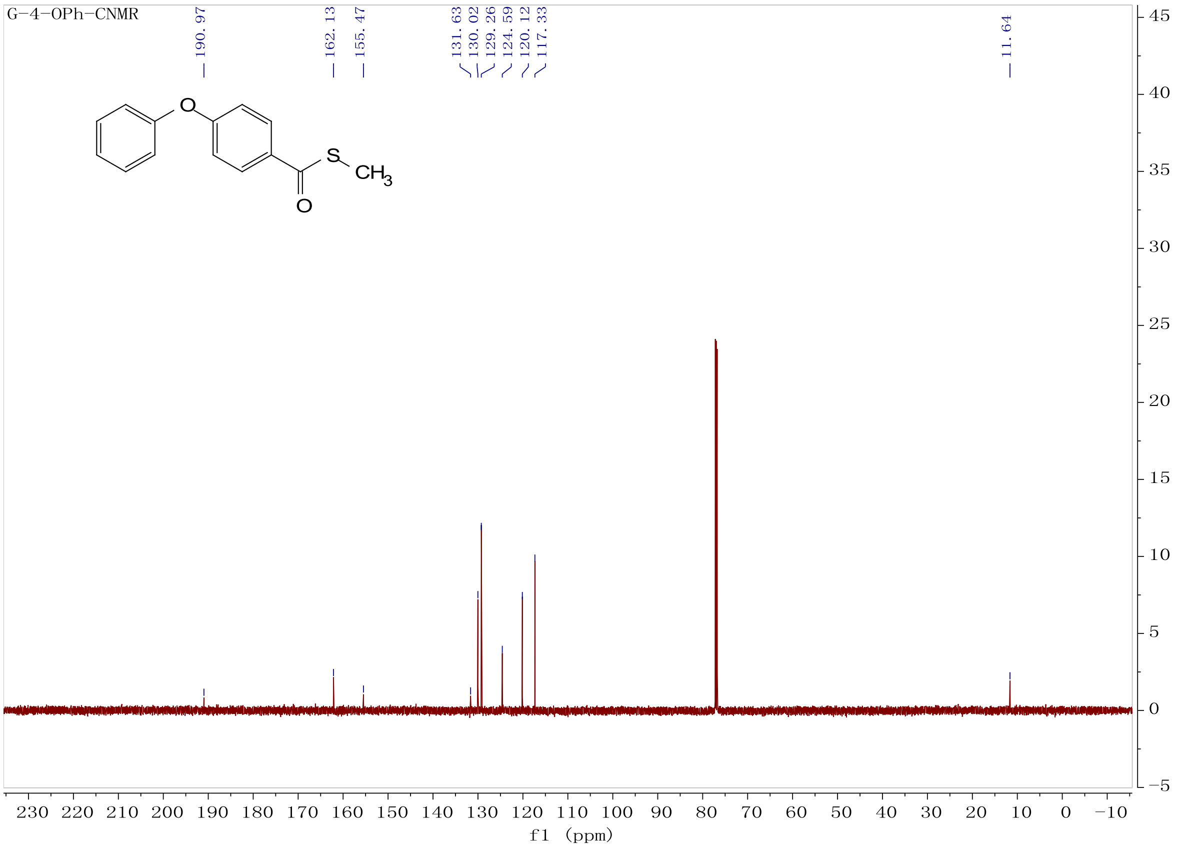
G-4-OPh-CNMR
190.97
162.13
155.47
131.63
130.02
129.26
124.59
120.12
117.33
11.64
O
S
CH3
O
f1 (ppm)

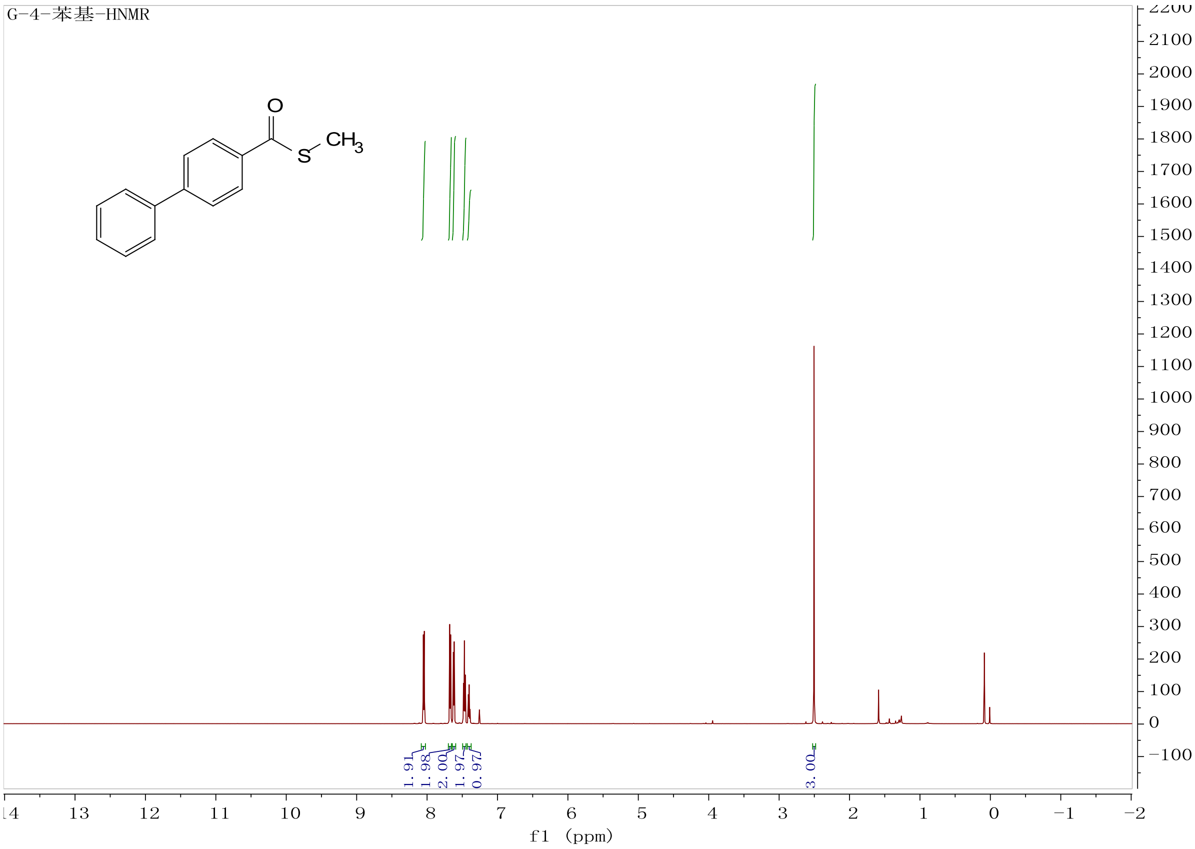
G-4-苯基-HNMR
O
S
CH3
1.91
1.98
2.00
1.97
0.97
3.00
f1 (ppm)

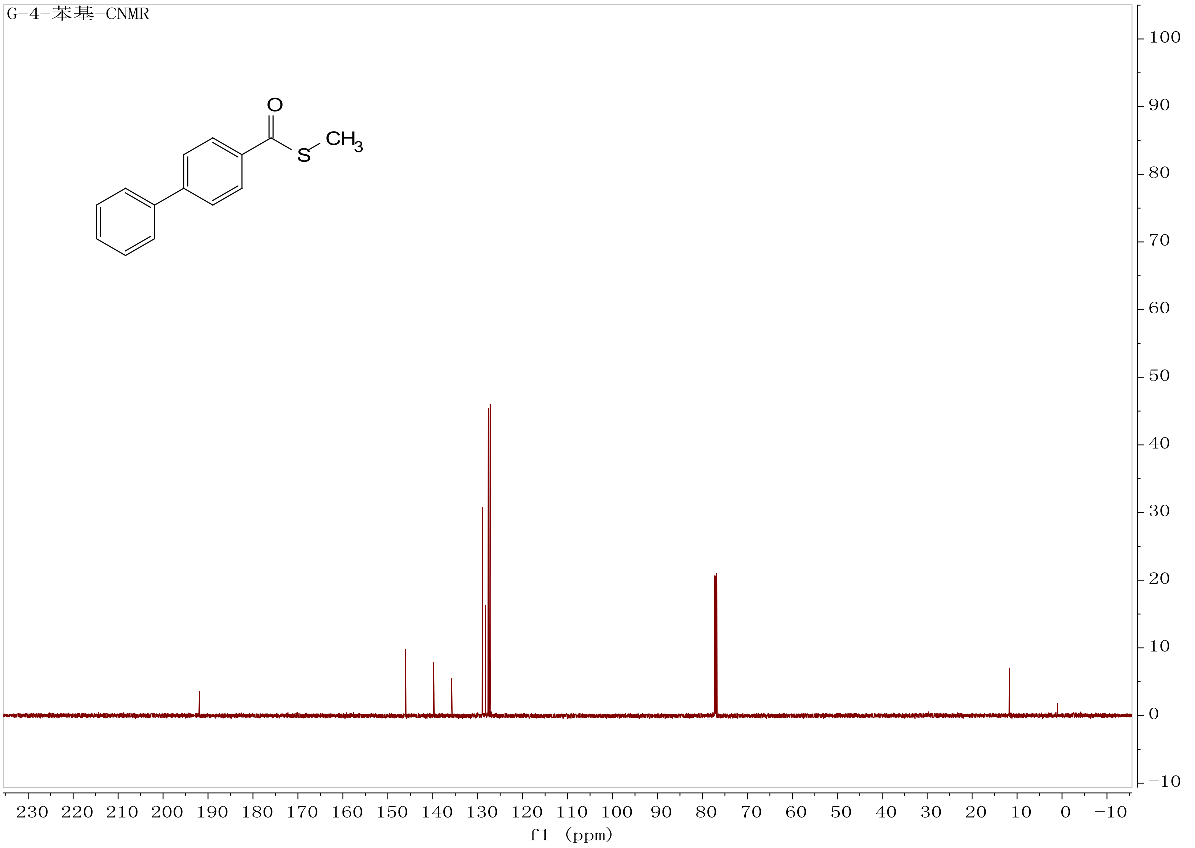
G-4-苯基-CNMR
O
S
CH3
f1 (ppm)

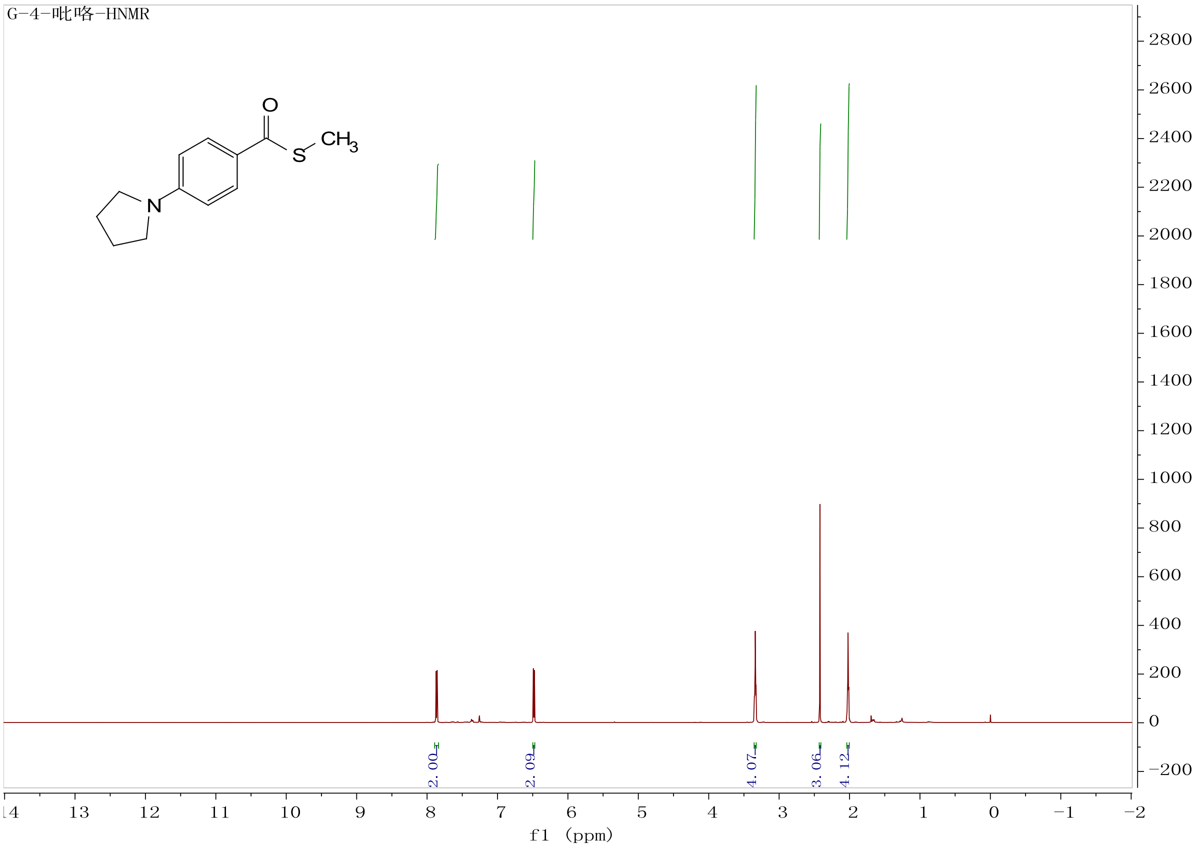
G-4-吡咯-HNMR
O
S
CH3
N
2.00
2.09
4.07
3.06
4.12
f1 (ppm)

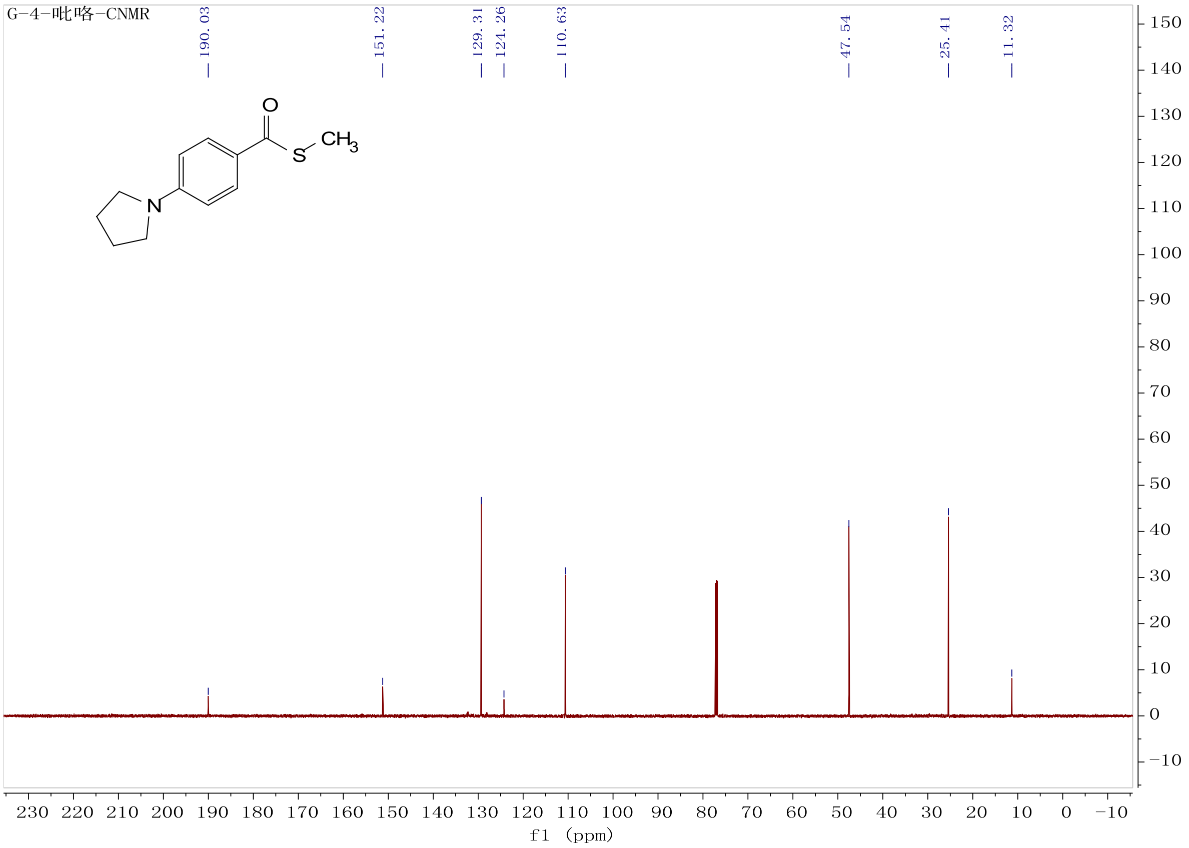
G-4-吡咯-CNMR
190.03
151.22
129.31
124.26
110.63
47.54
25.41
11.32
O
S
CH3
N
f1 (ppm)

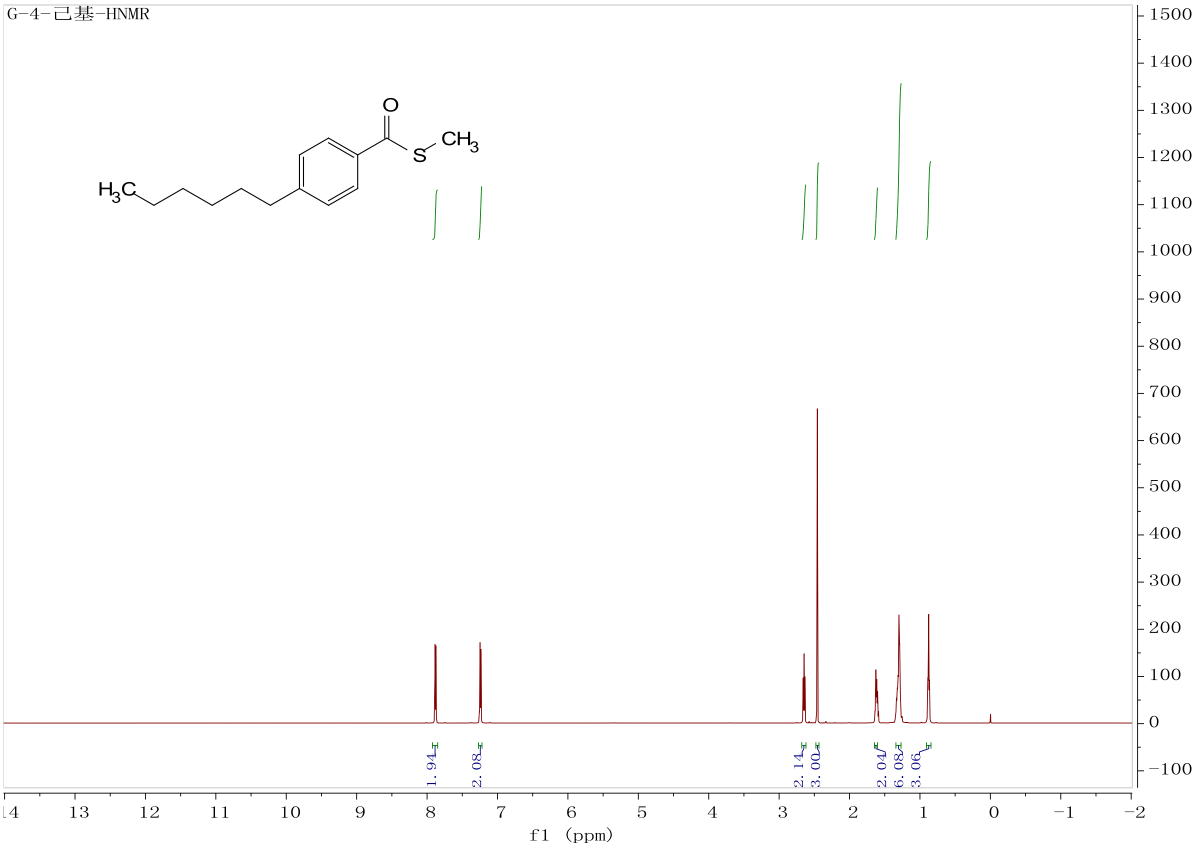

G-4-己基-HNMR
O
S
CH3
H3C
1.94
2.08
2.14
3.00
2.04
6.08
3.06
f1 (ppm)

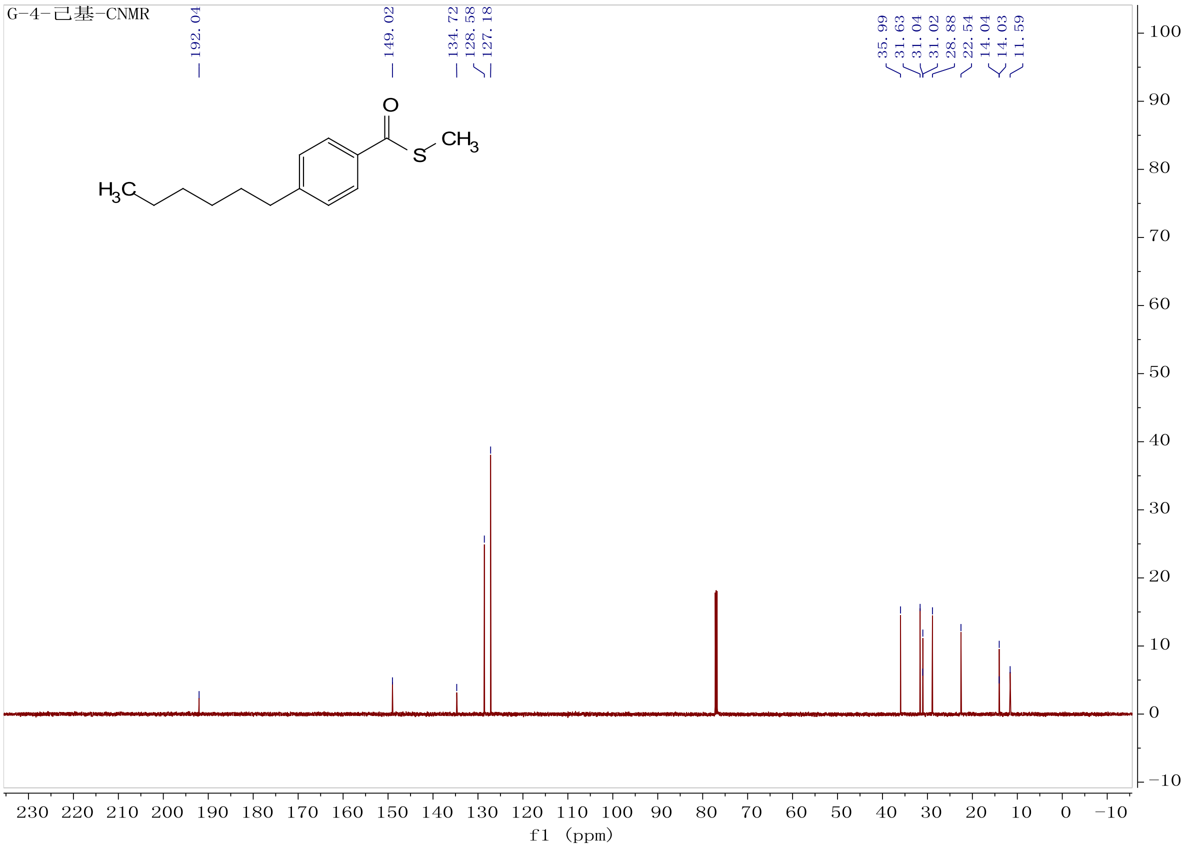

G-4-己基-CNMR
192.04
149.02
134.72
128.58
127.18
35.99
31.63
31.04
31.02
28.88
22.54
14.04
14.03
11.59
O
S
CH3
H3C
f1 (ppm)

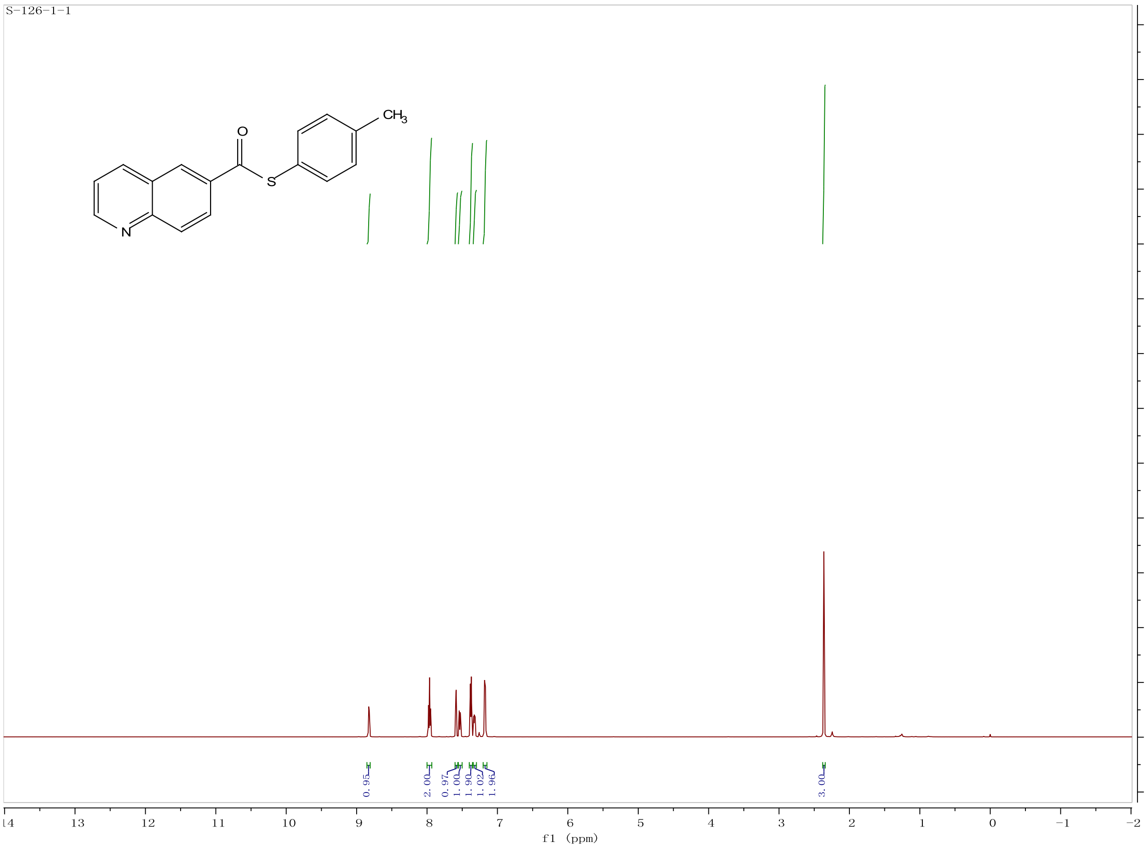
S-126-1-1
CH3
O
S
N
0.95
2.00
0.97
1.00
1.90
1.02
1.96
3.00
f1 (ppm)

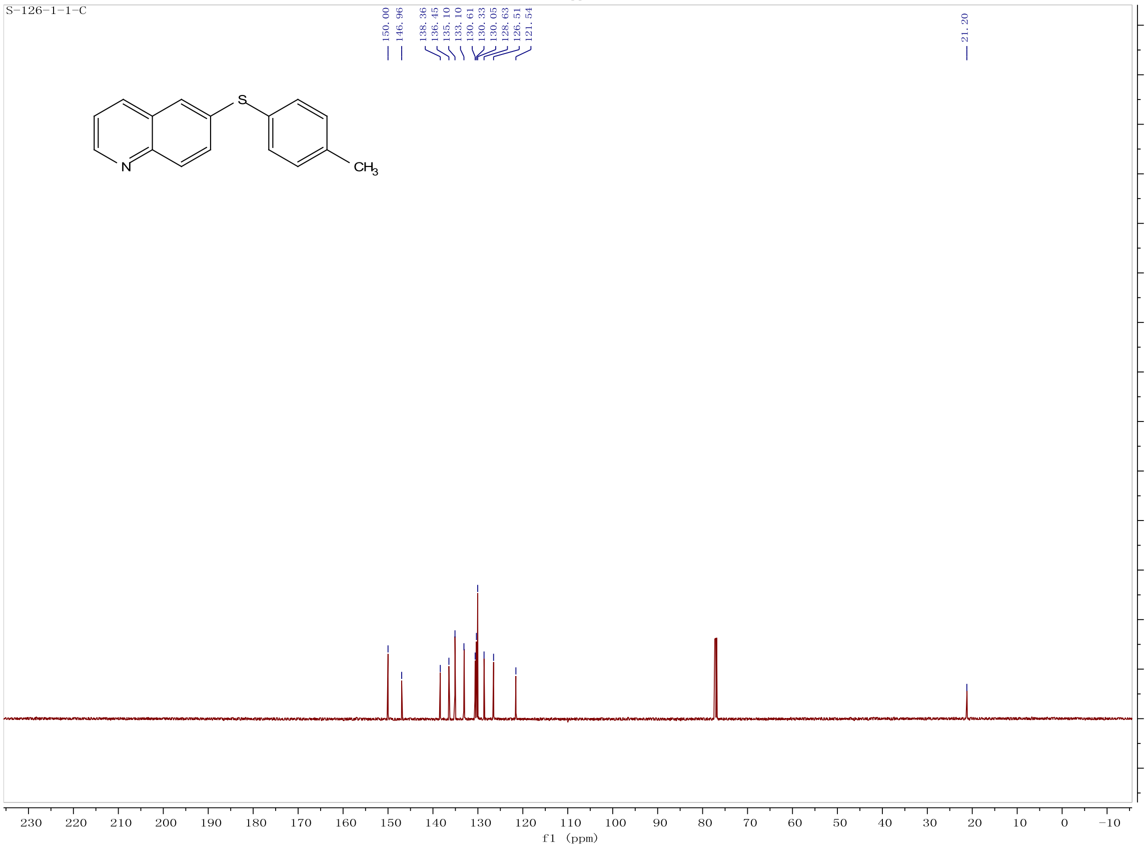
S-126-1-1-C
150.00
146.96
138.36
136.45
135.10
133.10
130.61
130.33
130.05
128.63
126.51
121.54
21.20
S
N
CH3
f1 (ppm)

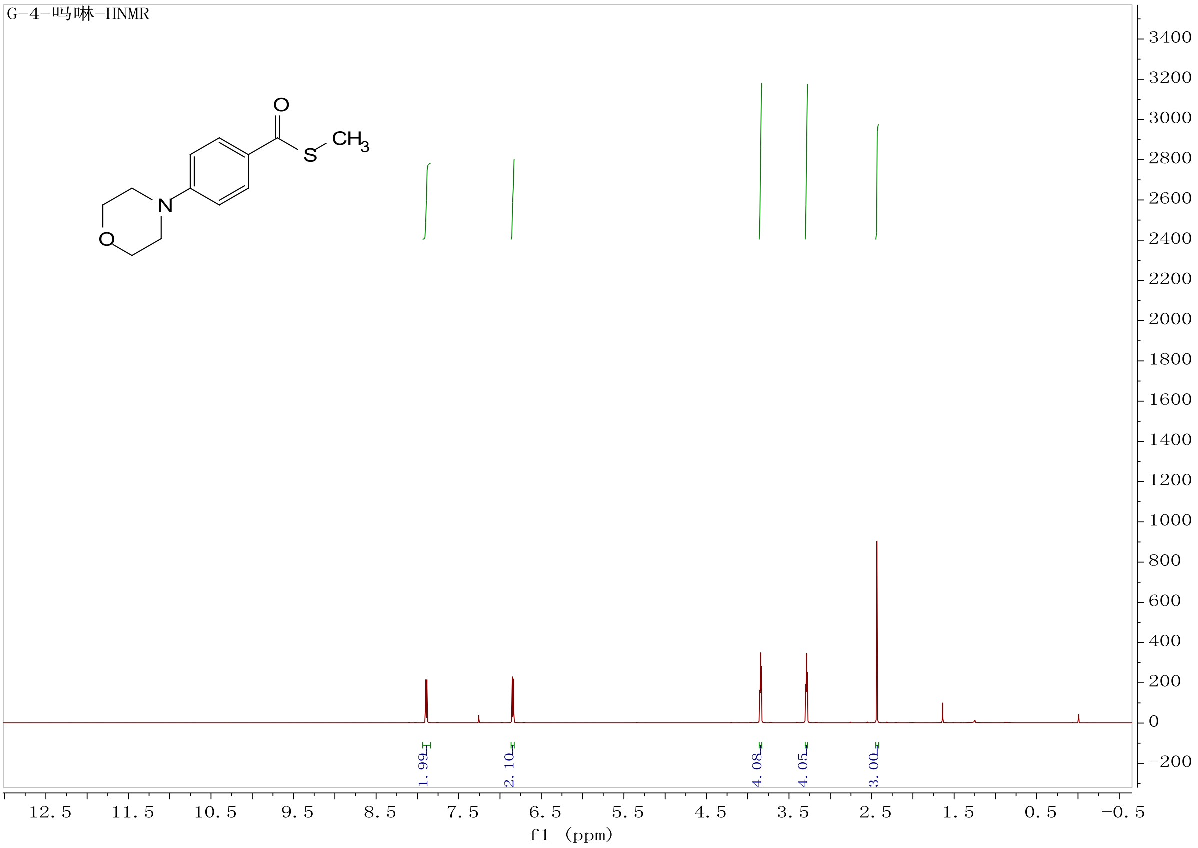
G-4-吗啉-HNMR
1.99
2.10
4.08
4.05
3.00
f1 (ppm)

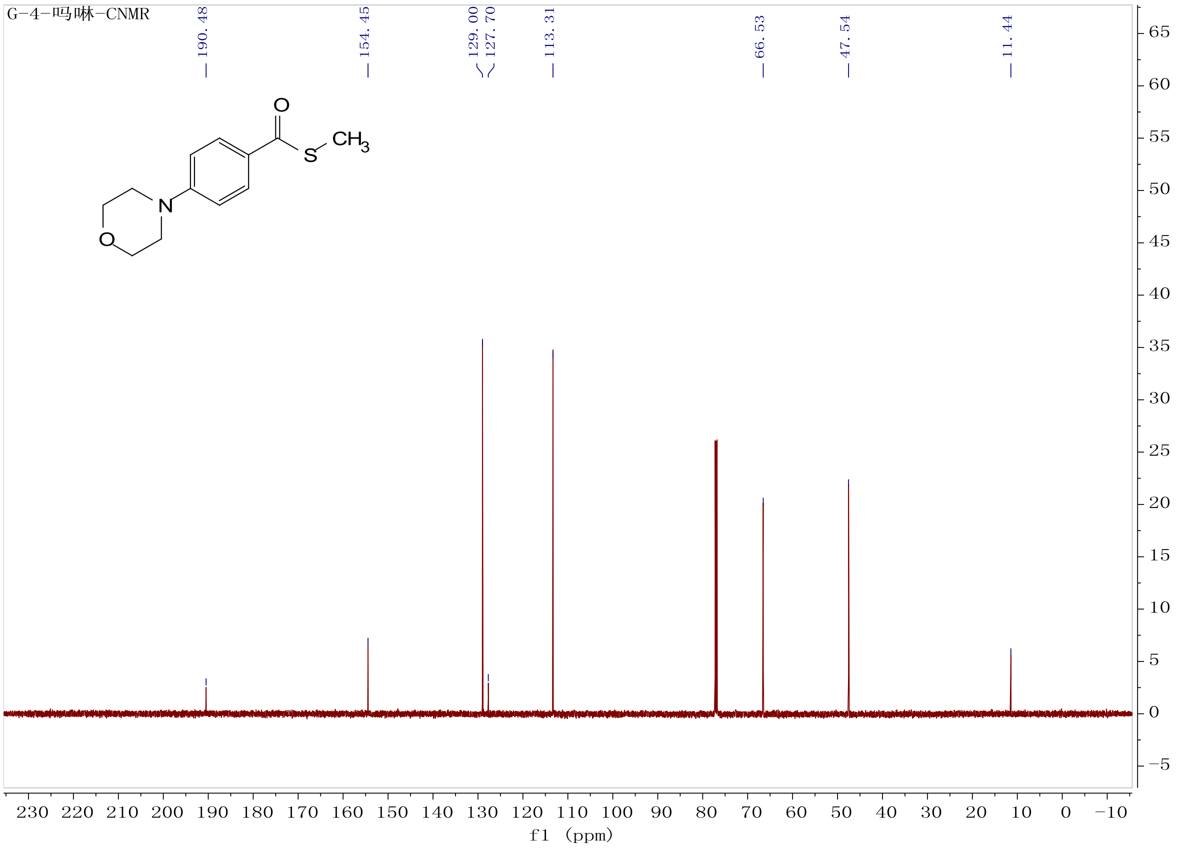
G-4-吗啉-CNMR
190.48
154.45
129.00
127.70
113.31
66.53
47.54
11.44
f1 (ppm)

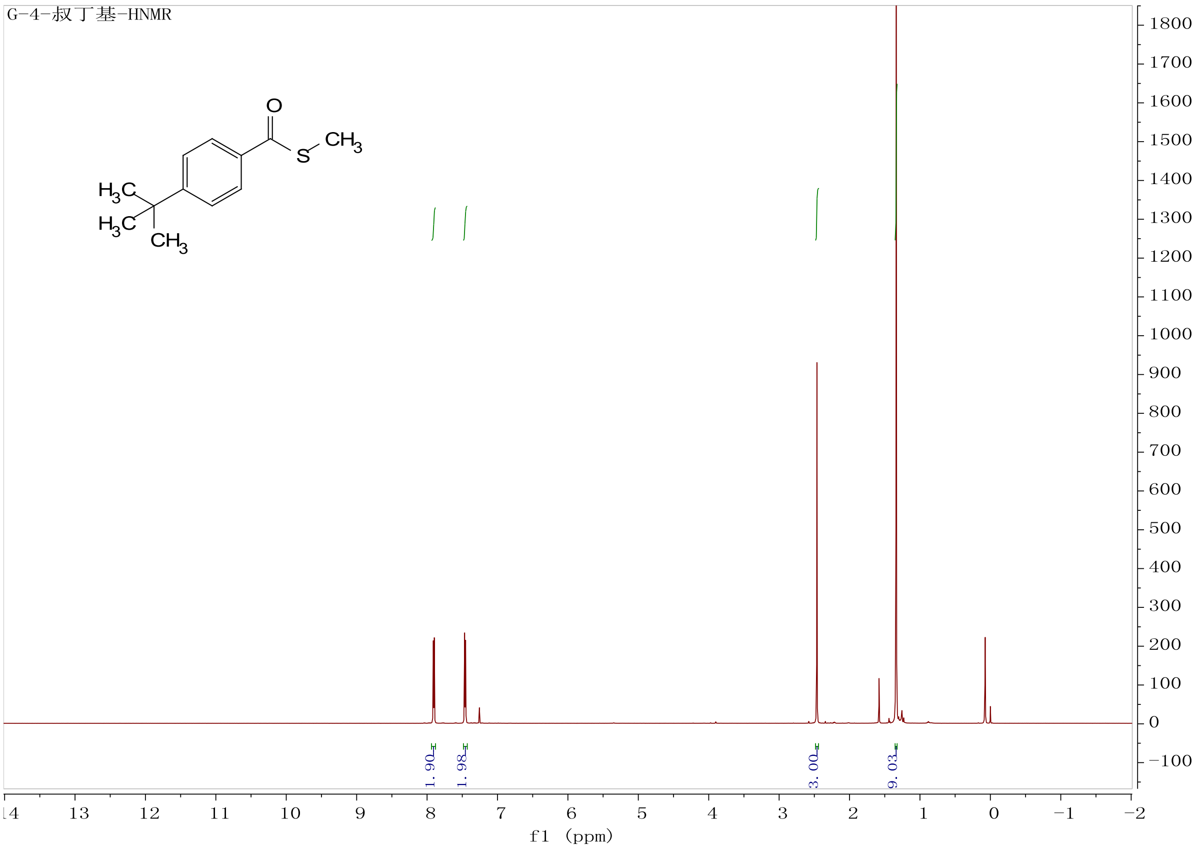
G-4-叔丁基-HNMR
1.90
1.98
3.00
9.03
f1 (ppm)

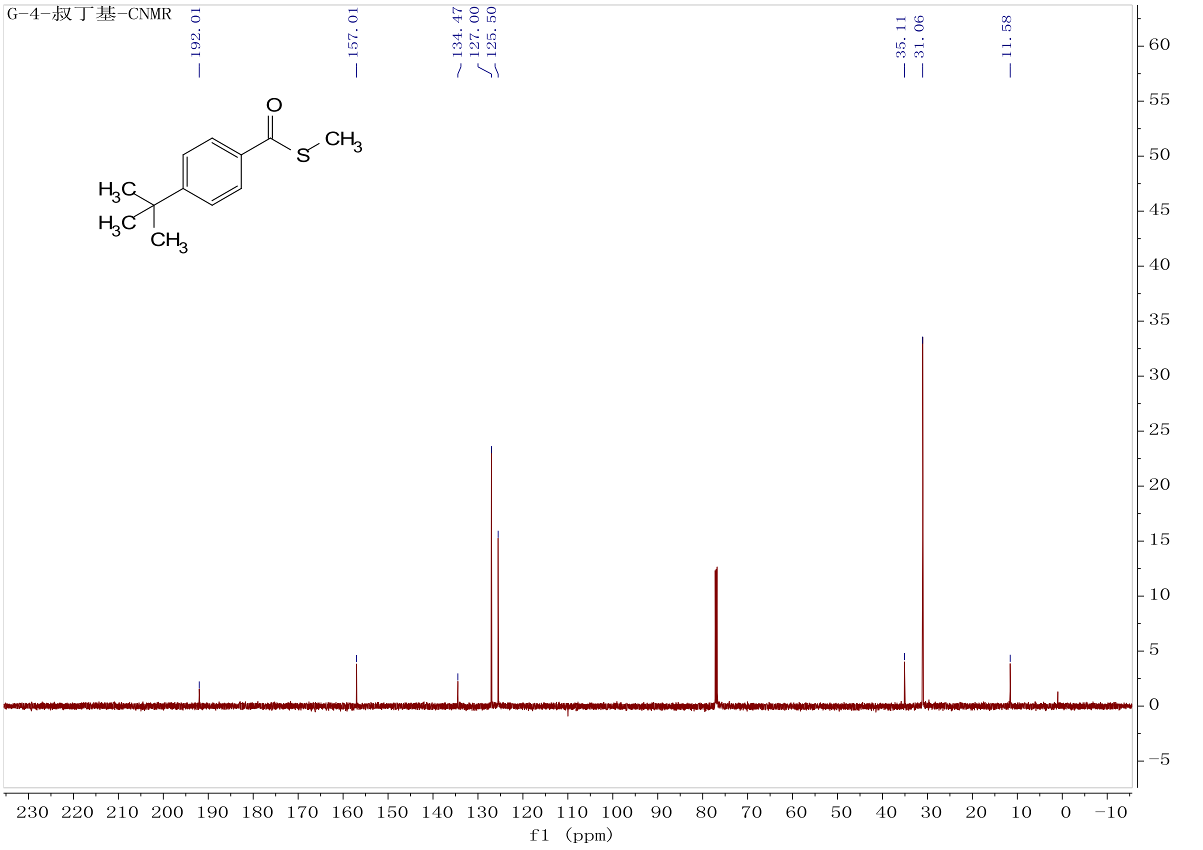
G-4-叔丁基-CNMR
192.01
157.01
134.47
127.00
125.50
35.11
31.06
11.58
f1 (ppm)

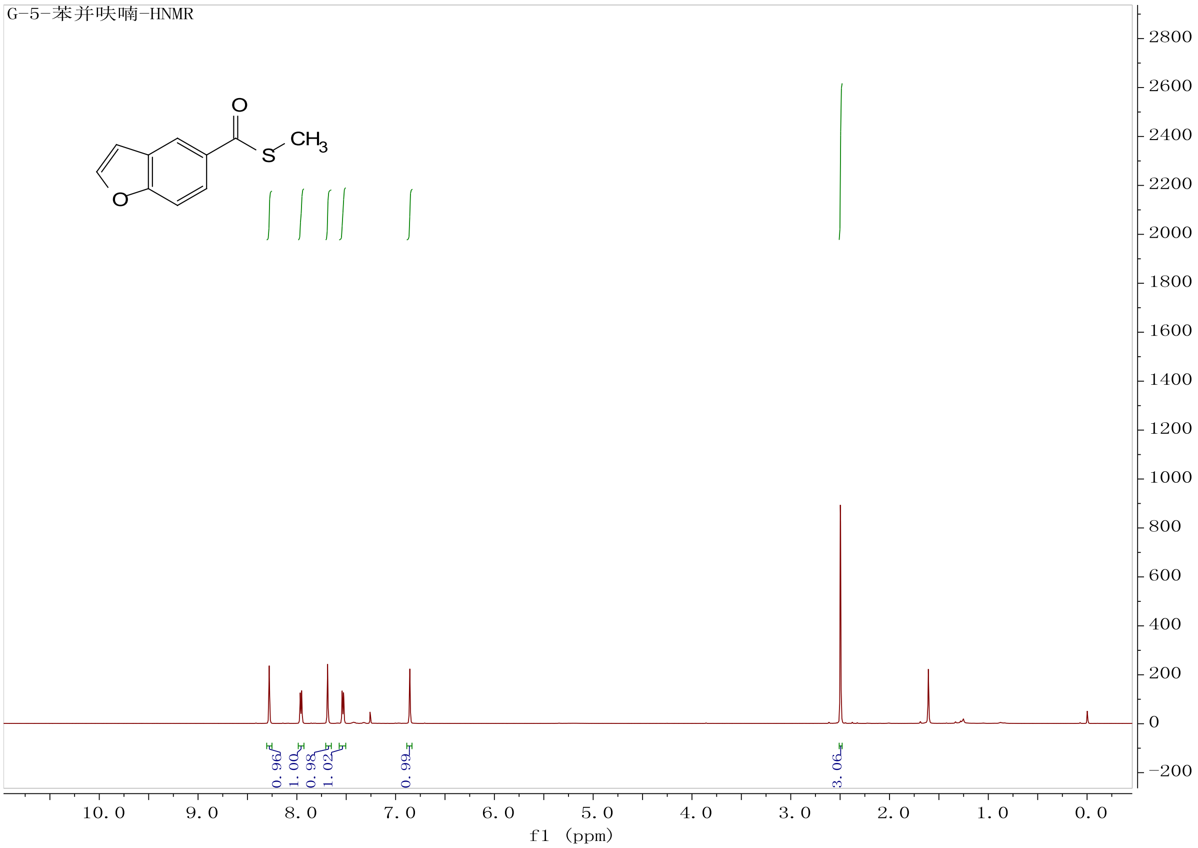
G-5-苯并呋喃-HNMR
O
S
CH3
0.96
1.00
0.98
1.02
0.99
3.06
10.0 9.0 8.0 7.0 6.0 5.0 4.0 3.0 2.0 1.0 0.0
f1 (ppm)
2800 2600 2400 2200 2000 1800 1600 1400 1200 1000 800 600 400 200 0 -200

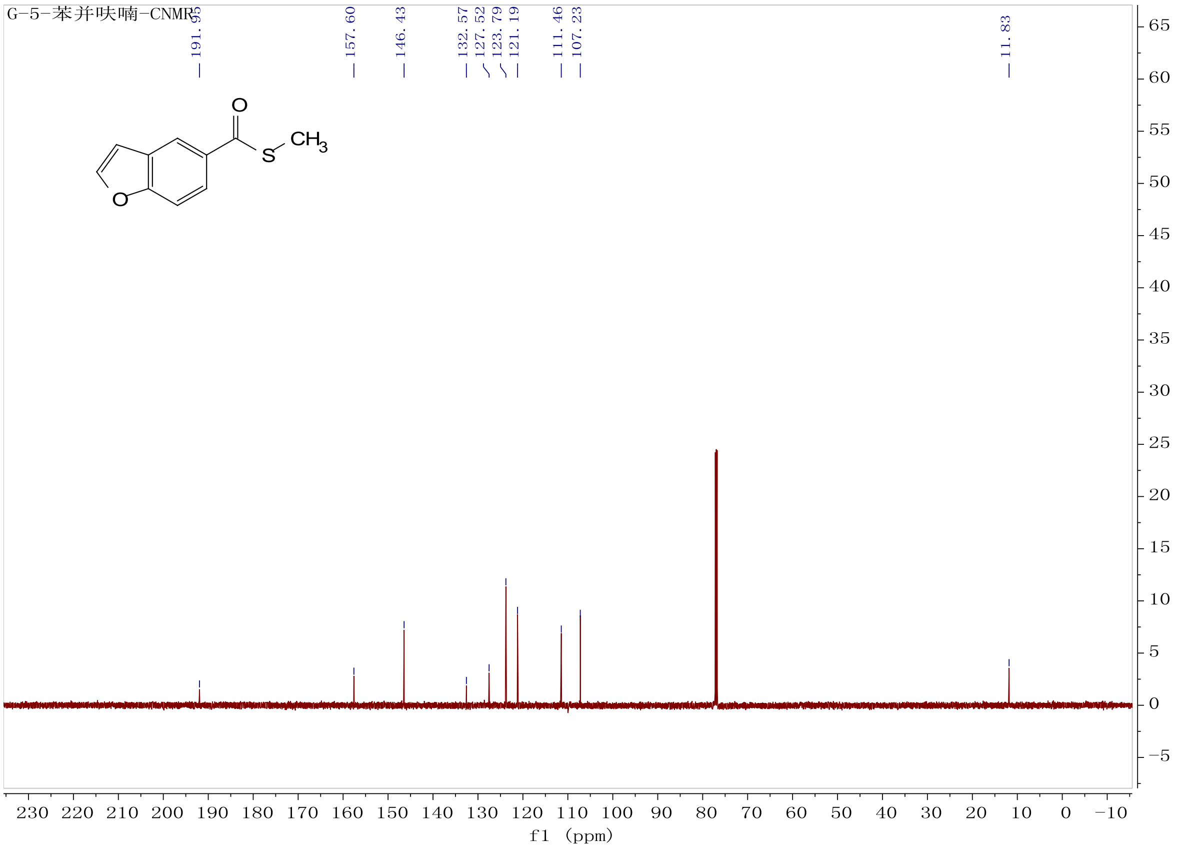
G-5-苯并呋喃-CNMR
191.95
157.60
146.43
132.57
127.52
123.79
121.19
111.46
107.23
11.83
O
S
CH3
230 220 210 200 190 180 170 160 150 140 130 120 110 100 90 80 70 60 50 40 30 20 10 0 -10
f1 (ppm)
65 60 55 50 45 40 35 30 25 20 15 10 5 0 -5

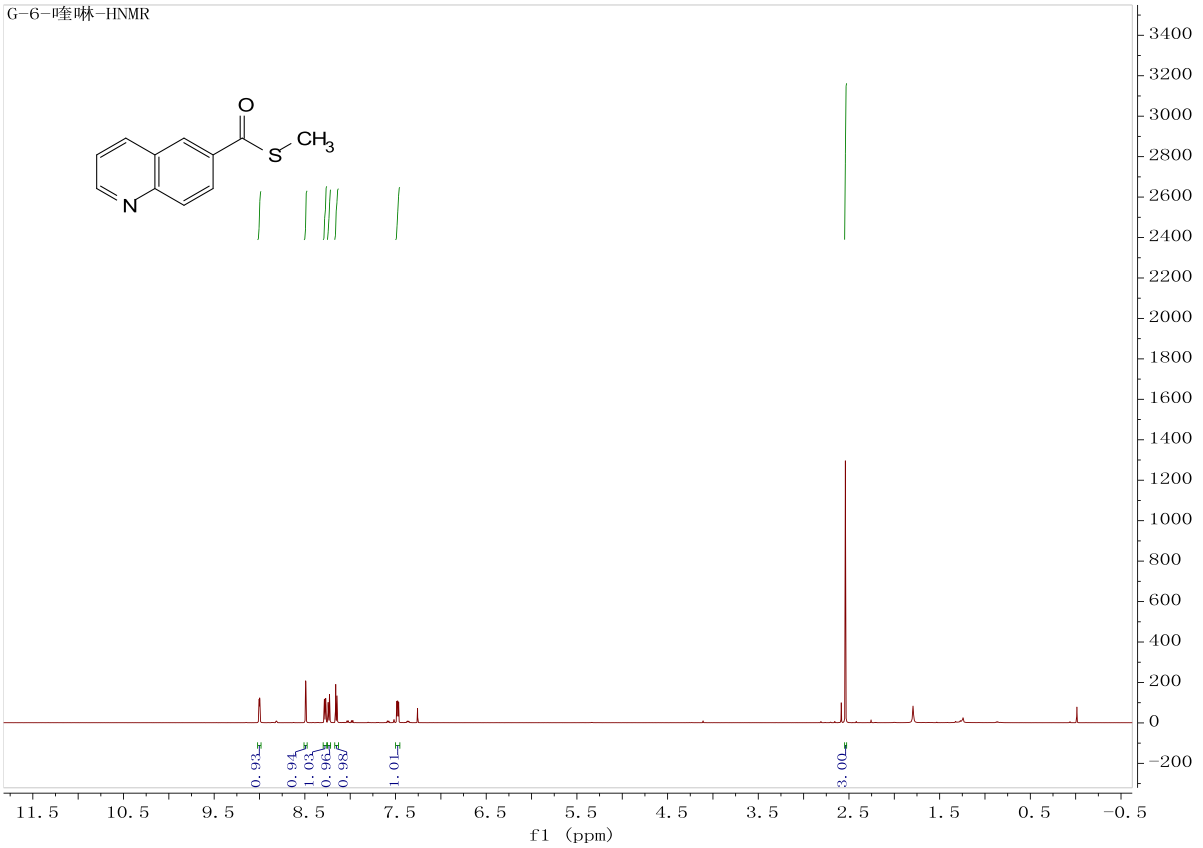
G-6-喹啉-HNMR
O
S
CH3
N
0.93
0.94
1.03
0.96
0.98
1.01
3.00
11.5 10.5 9.5 8.5 7.5 6.5 5.5 4.5 3.5 2.5 1.5 0.5 -0.5
f1 (ppm)

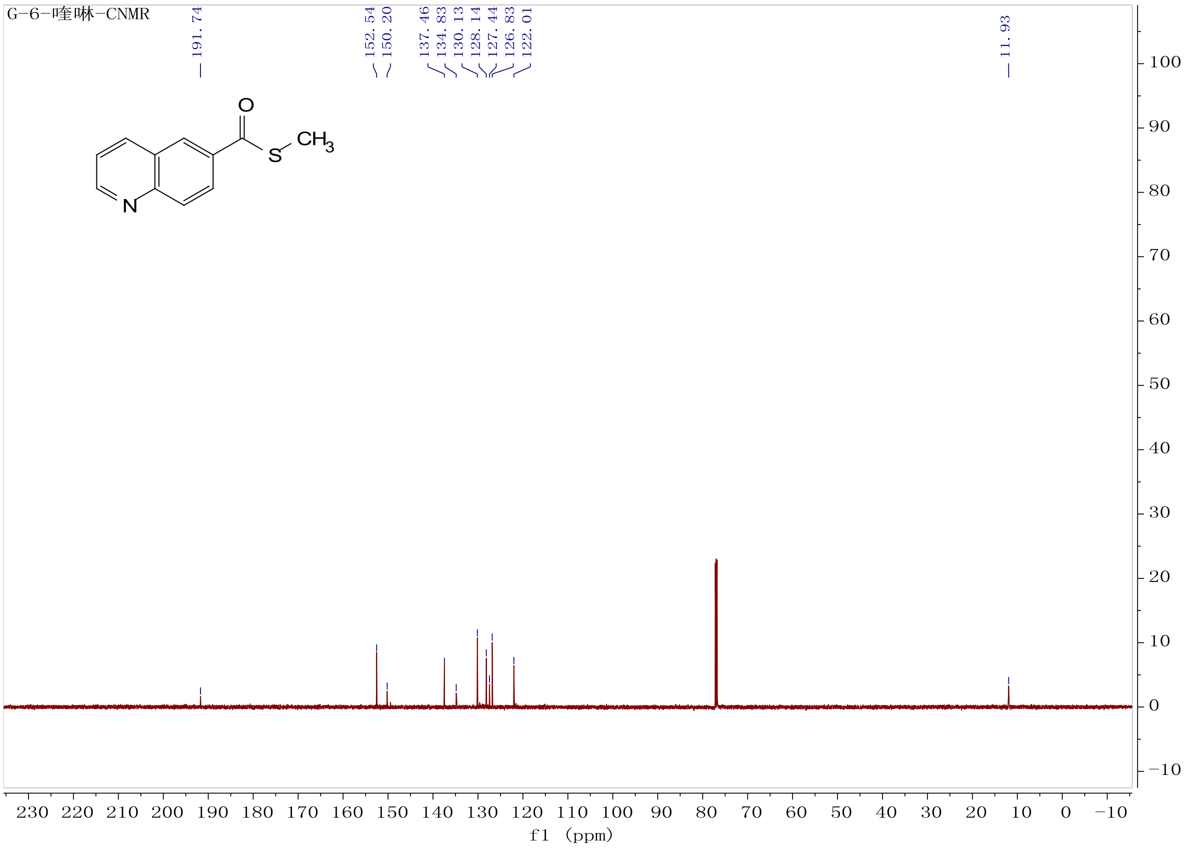
G-6-喹啉-CNMR
191.74
152.54
150.20
137.46
134.83
130.13
128.14
127.44
126.83
122.01
11.93
O
S
CH3
N
230 220 210 200 190 180 170 160 150 140 130 120 110 100 90 80 70 60 50 40 30 20 10 0 -10
f1 (ppm)

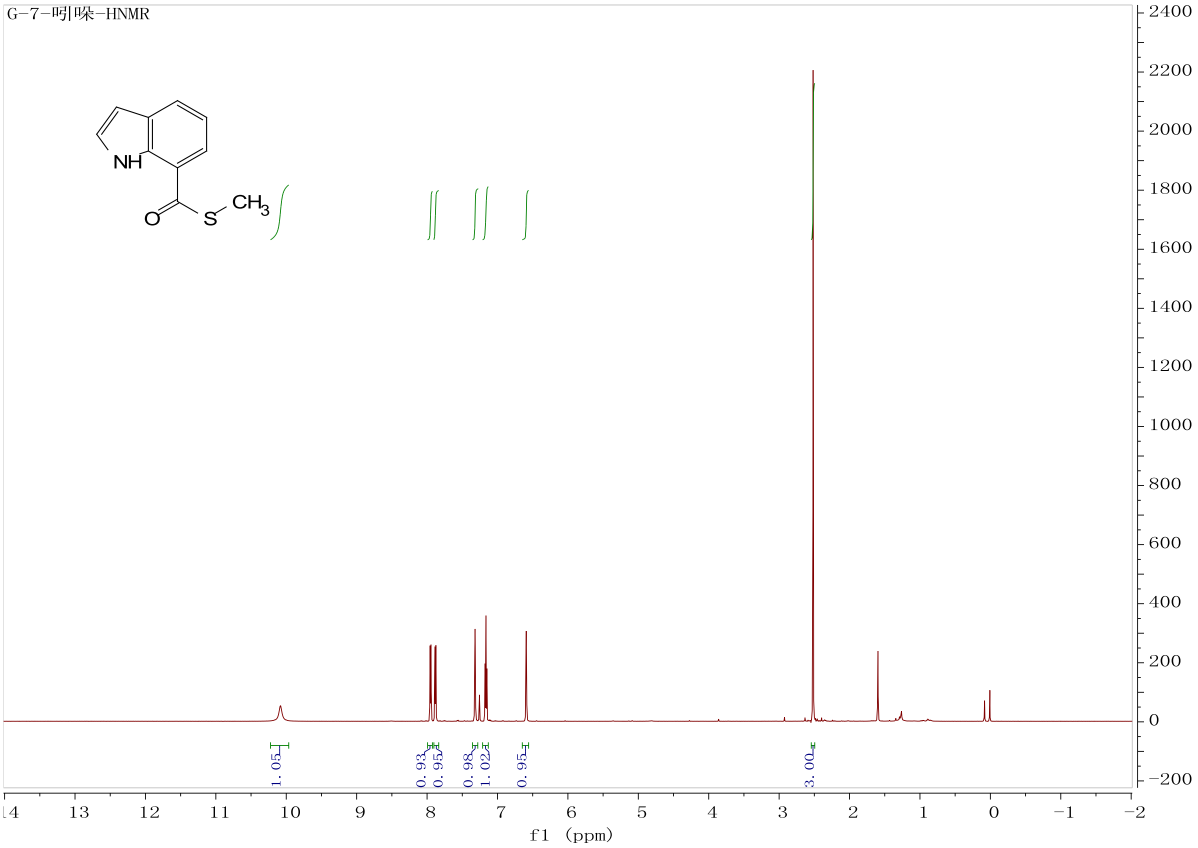

G-7-吲哚-HNMR
NH
O
S
CH3
1.05
0.93
0.95
0.98
1.02
0.95
3.00
f1 (ppm)

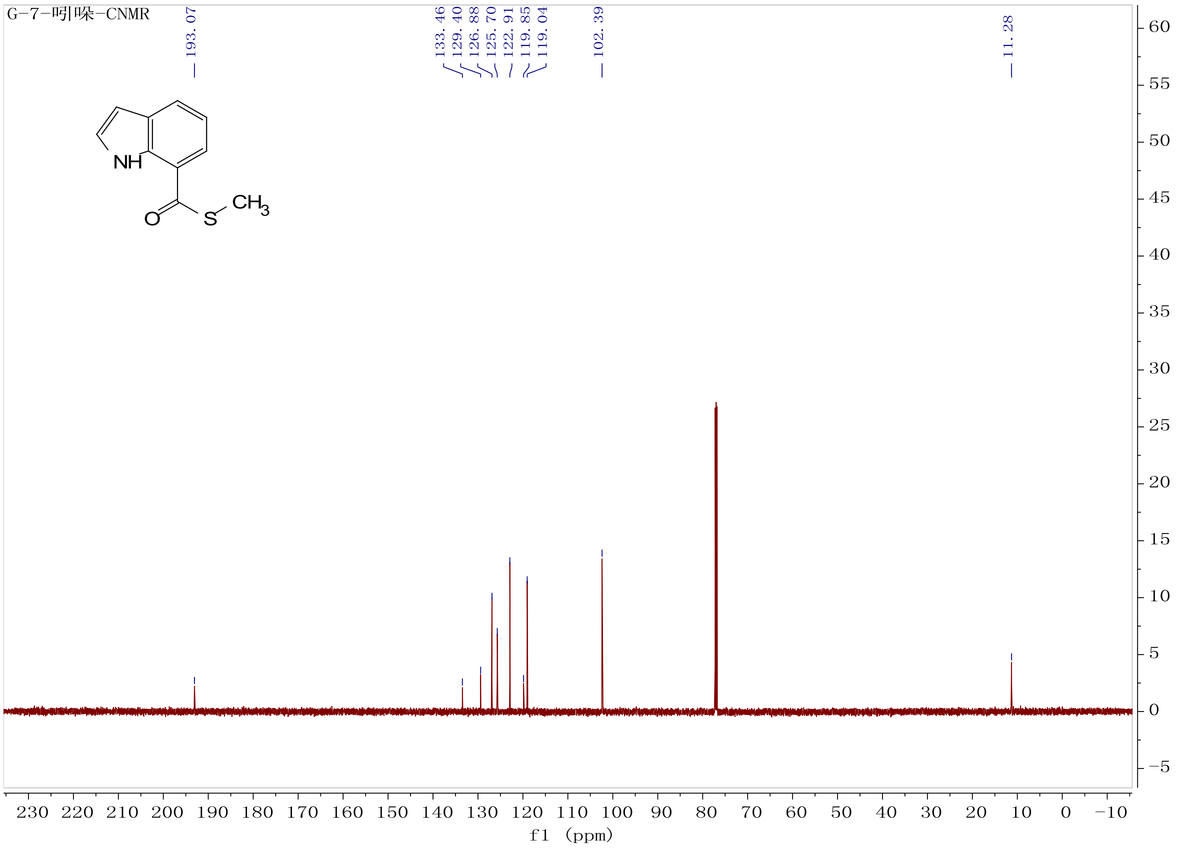

G-7-吲哚-CNMR
193.07
133.46
129.40
126.88
125.70
122.91
119.85
119.04
102.39
11.28
NH
O
S
CH3
f1 (ppm)

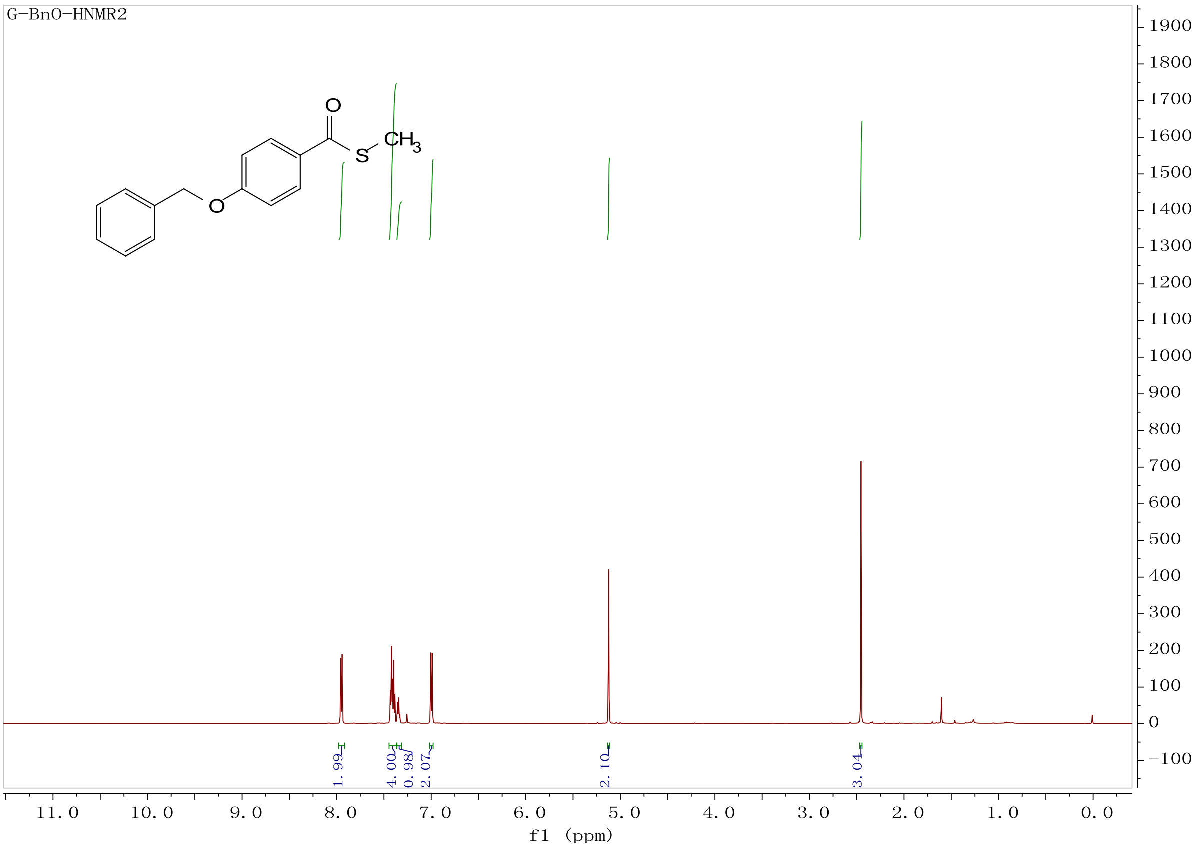
G-BnO-HNMR2
O
S
CH3
O
1.99
4.00
0.98
2.07
2.10
3.04
11.0 10.0 9.0 8.0 7.0 6.0 5.0 4.0 3.0 2.0 1.0 0.0
f1 (ppm)
1900 1800 1700 1600 1500 1400 1300 1200 1100 1000 900 800 700 600 500 400 300 200 100 0 −100

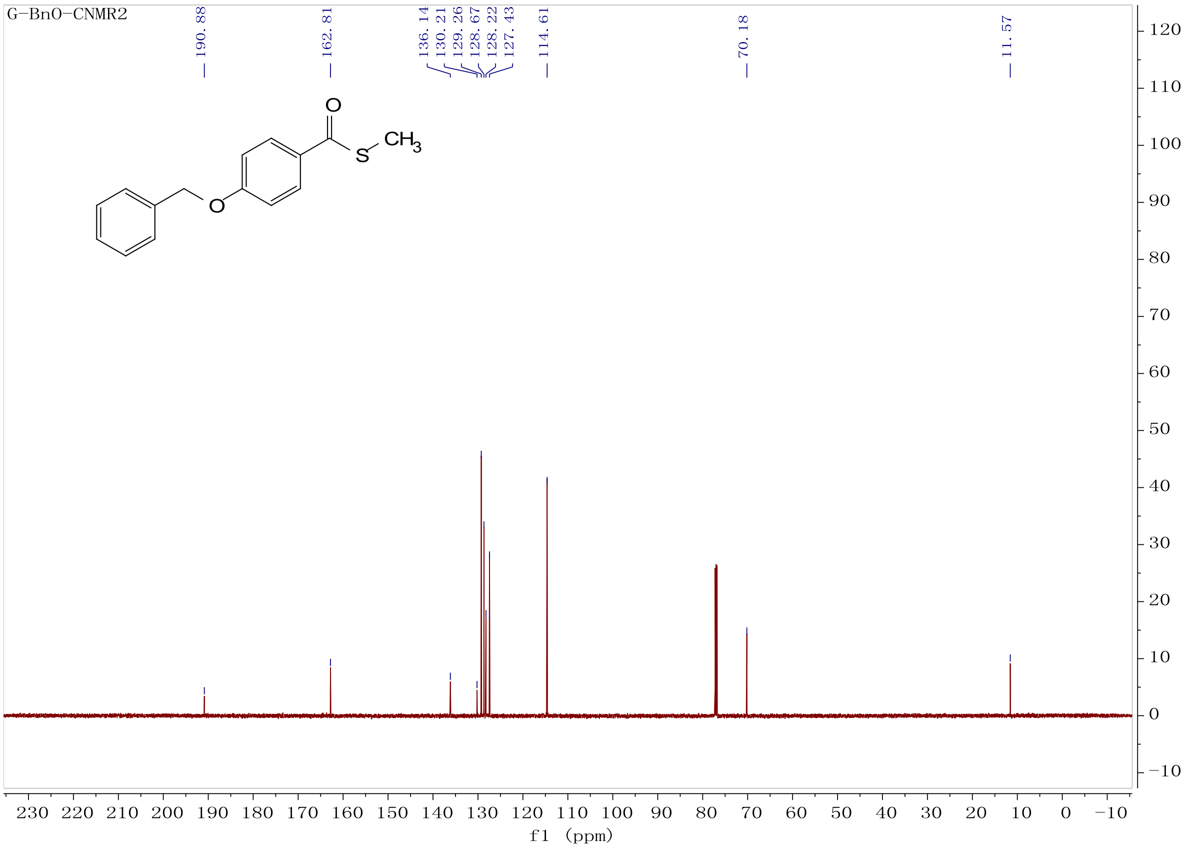
G-BnO-CNMR2
190.88
162.81
136.14
130.21
129.26
128.67
128.22
127.43
114.61
70.18
11.57
O
S
CH3
O
230 220 210 200 190 180 170 160 150 140 130 120 110 100 90 80 70 60 50 40 30 20 10 0 −10
f1 (ppm)
120 110 100 90 80 70 60 50 40 30 20 10 0 −10

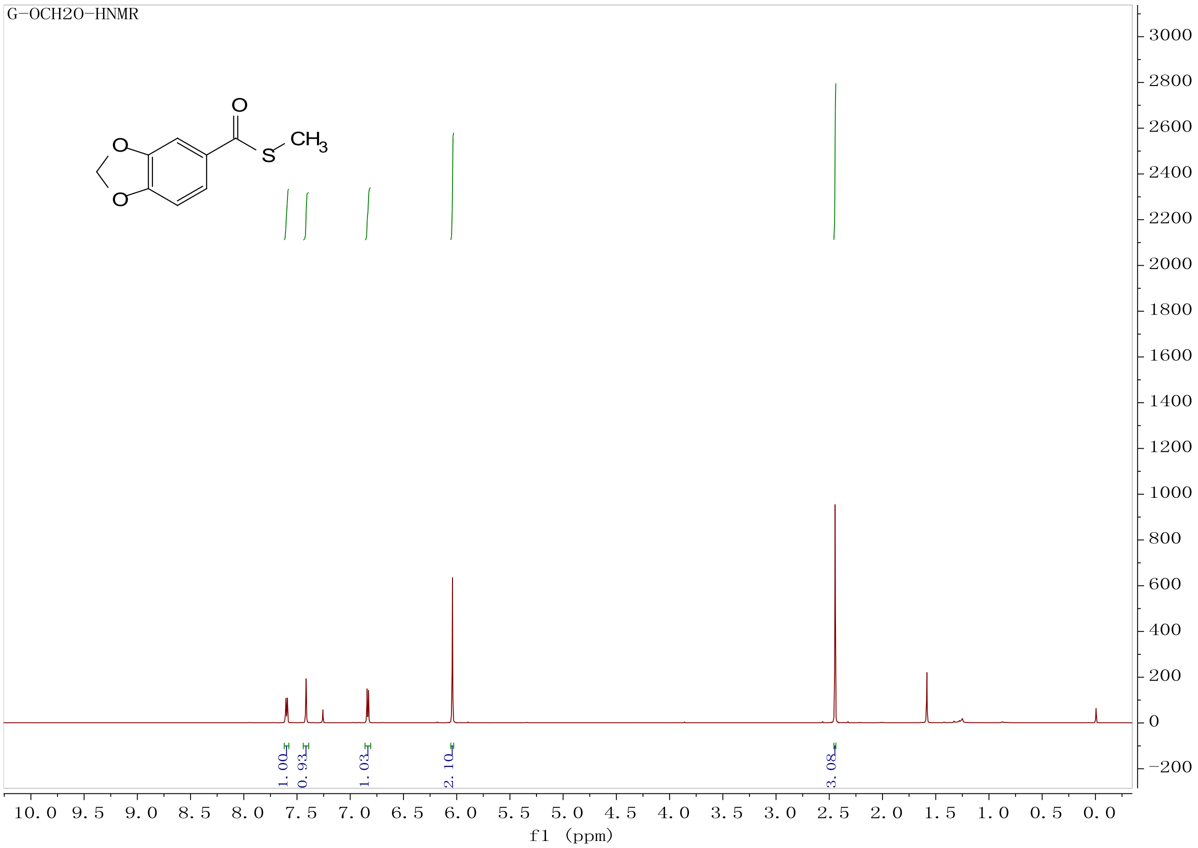
G-OCH2O-HNMR
O
CH_3
S
O
O
1.00
0.93
1.03
2.10
3.08
10.0 9.5 9.0 8.5 8.0 7.5 7.0 6.5 6.0 5.5 5.0 4.5 4.0 3.5 3.0 2.5 2.0 1.5 1.0 0.5 0.0
f1 (ppm)
3000 2800 2600 2400 2200 2000 1800 1600 1400 1200 1000 800 600 400 200 0 -200

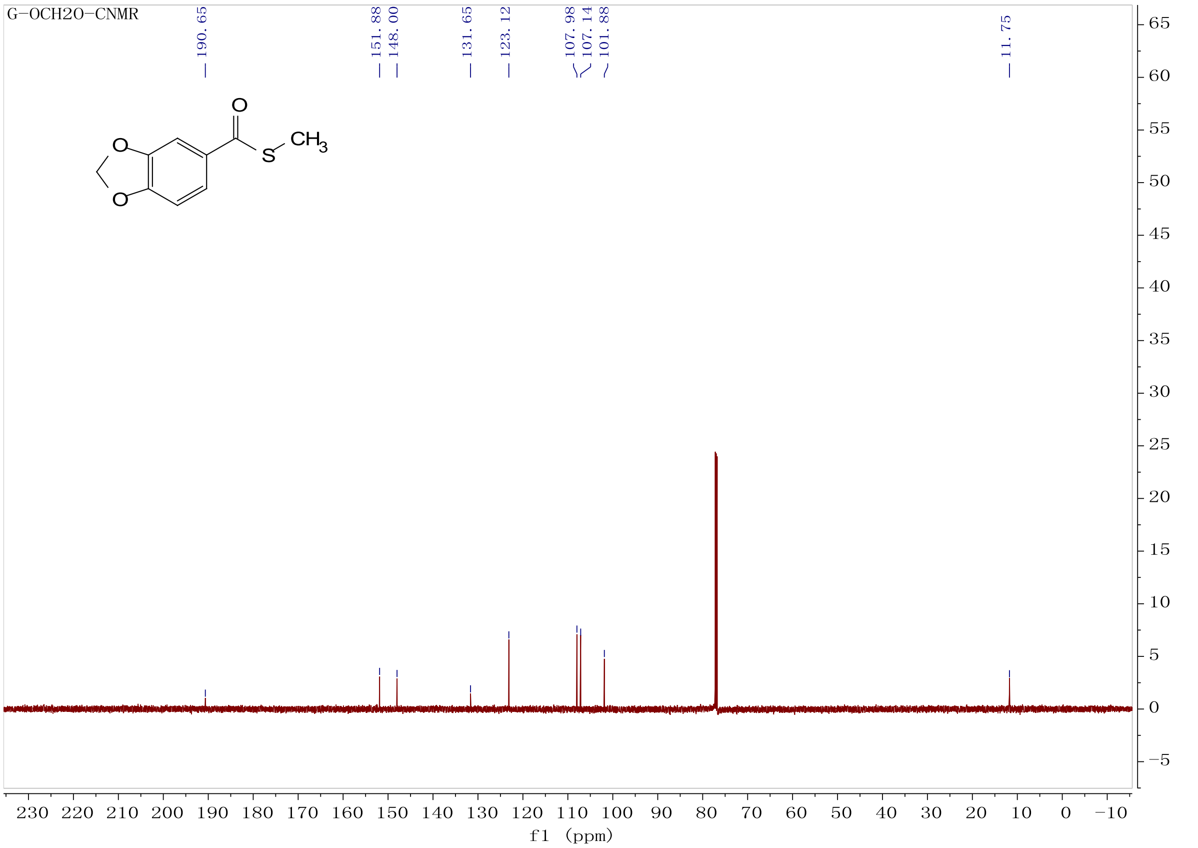
G-OCH2O-CNMR
190.65
151.88
148.00
131.65
123.12
107.98
107.14
101.88
11.75
230 220 210 200 190 180 170 160 150 140 130 120 110 100 90 80 70 60 50 40 30 20 10 0 -10
f1 (ppm)
65 60 55 50 45 40 35 30 25 20 15 10 5 0 -5

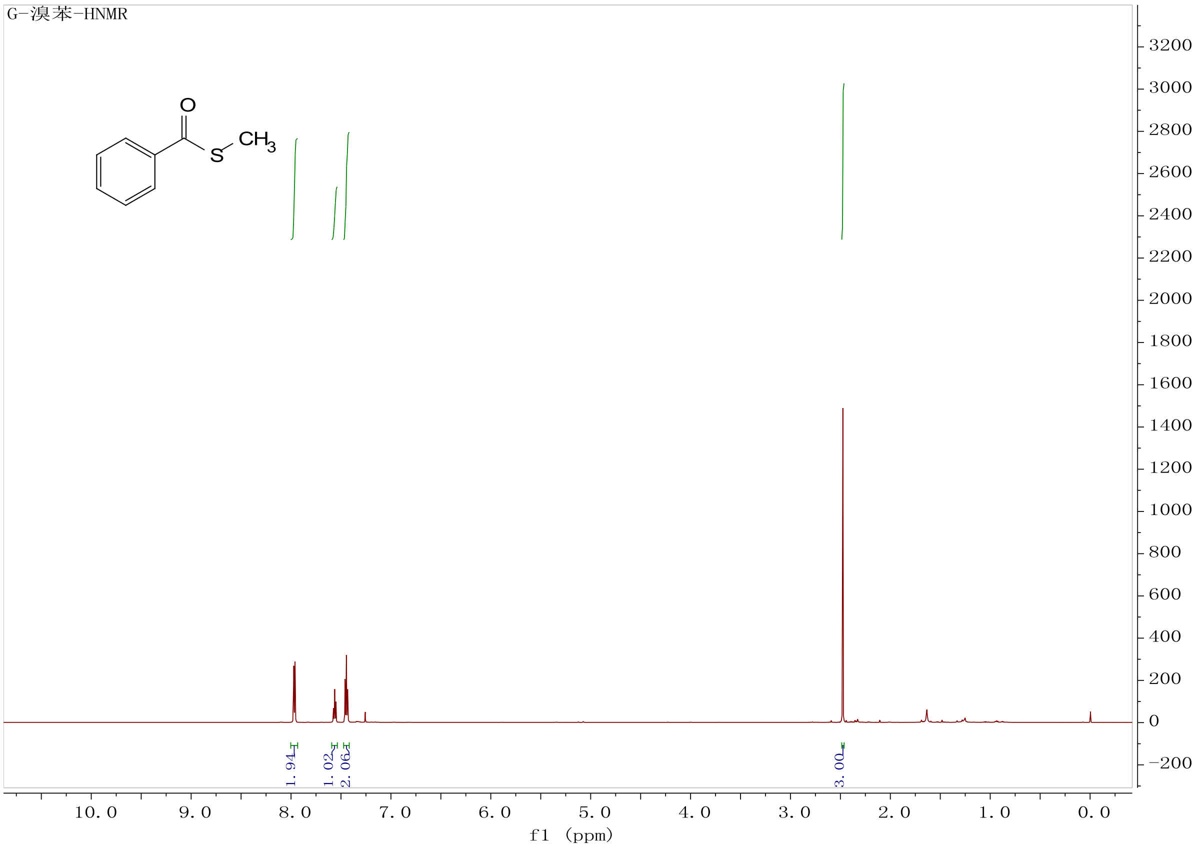
G-溴苯-HNMR
O
S
CH3
1.94
1.02
2.06
3.00
10.0 9.0 8.0 7.0 6.0 5.0 4.0 3.0 2.0 1.0 0.0
f1 (ppm)
3200 3000 2800 2600 2400 2200 2000 1800 1600 1400 1200 1000 800 600 400 200 0 −200

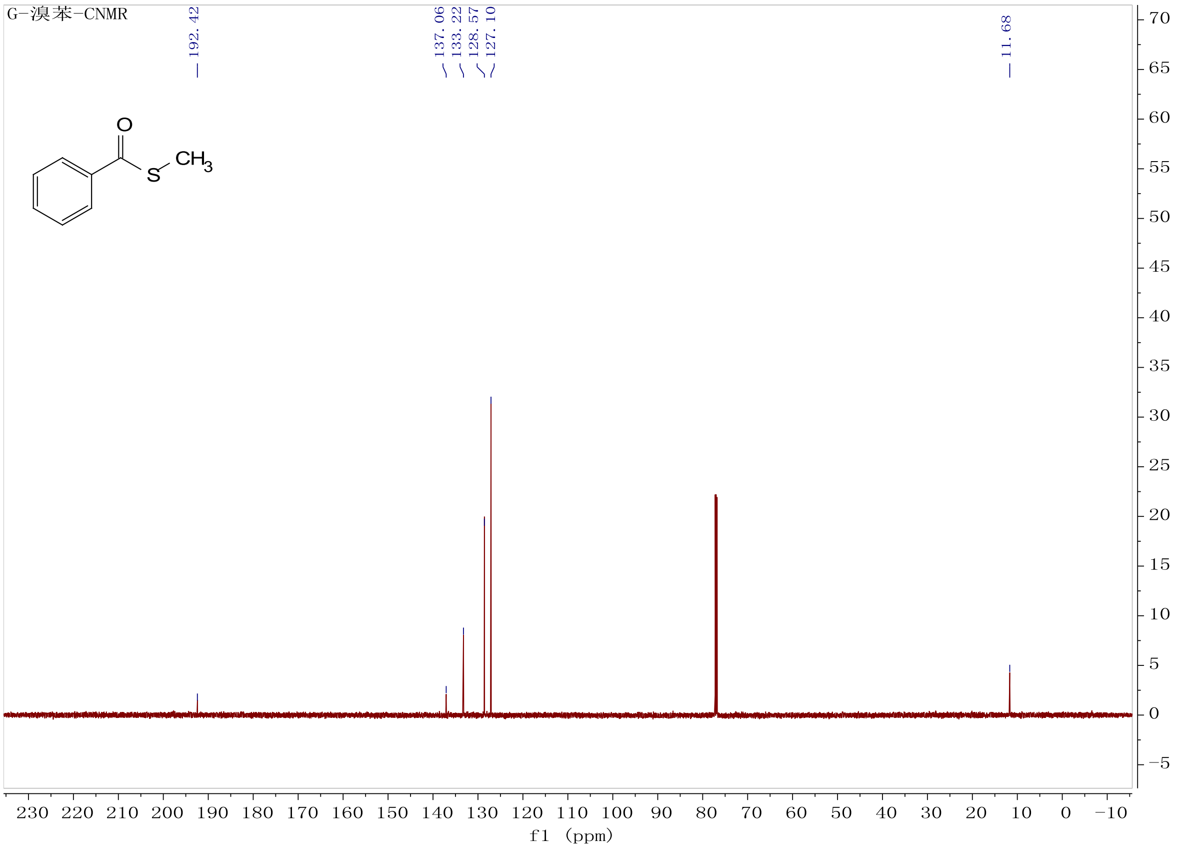
G-溴苯-CNMR
192.42
137.06
133.22
128.57
127.10
11.68
O
S
CH3
230 220 210 200 190 180 170 160 150 140 130 120 110 100 90 80 70 60 50 40 30 20 10 0 −10
f1 (ppm)
70 65 60 55 50 45 40 35 30 25 20 15 10 5 0 −5

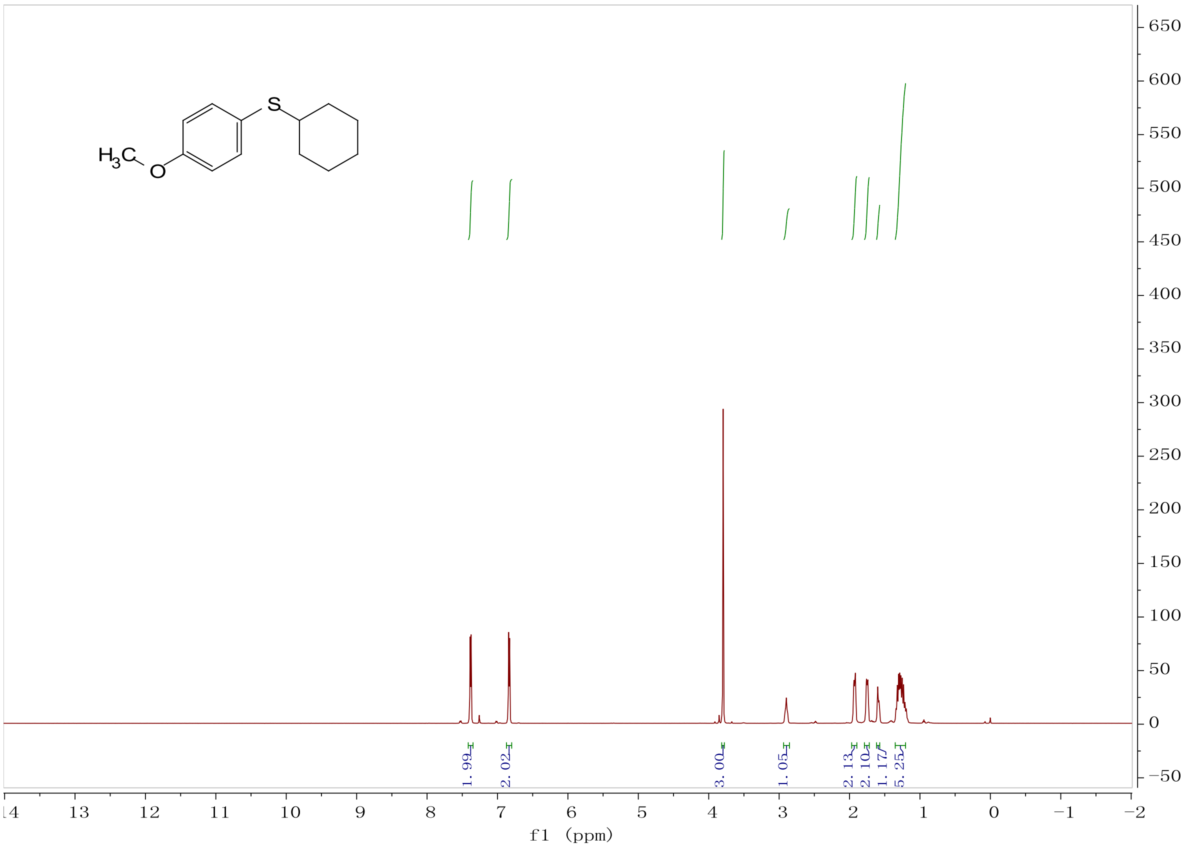

H3C
O
S
1.99
2.02
3.00
1.05
2.13
2.10
1.17
5.25
f1 (ppm)

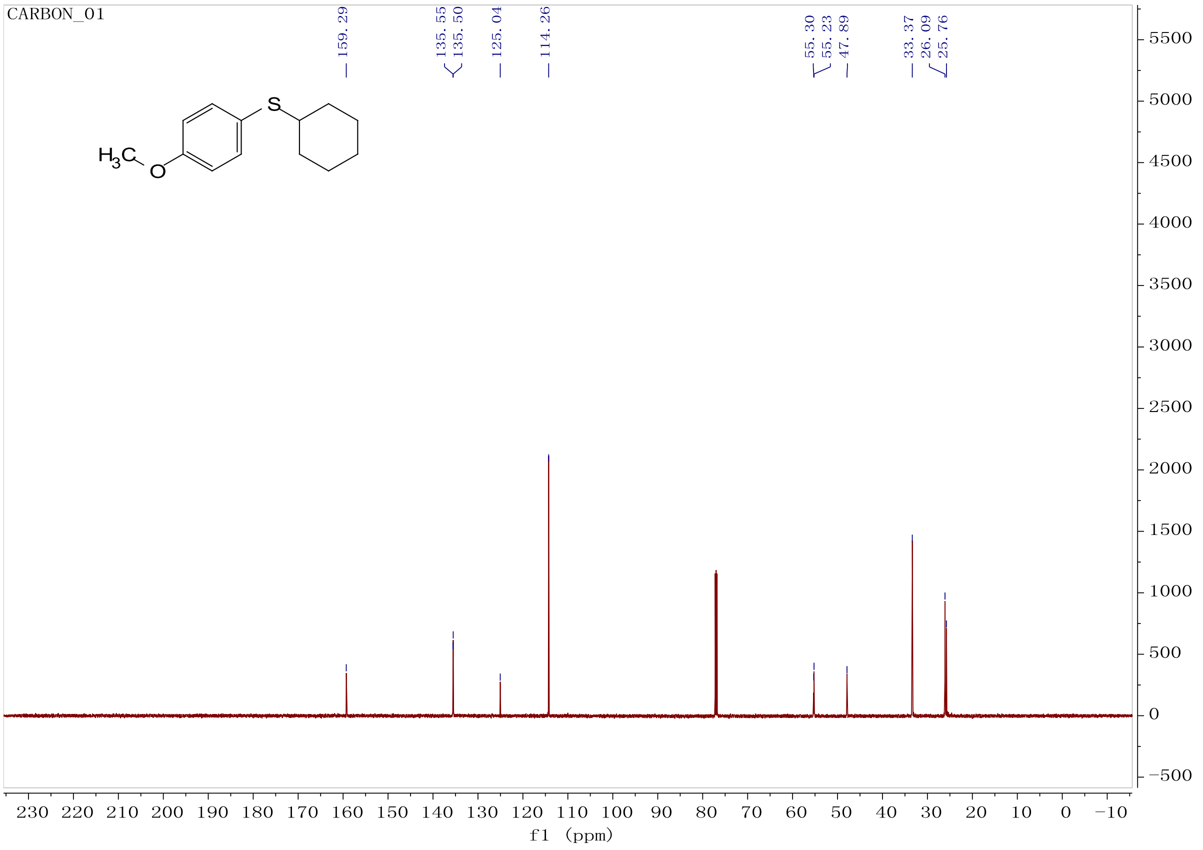

CARBON_01
159.29
135.55
135.50
125.04
114.26
55.30
55.23
47.89
33.37
26.09
25.76
H3C
O
S
f1 (ppm)

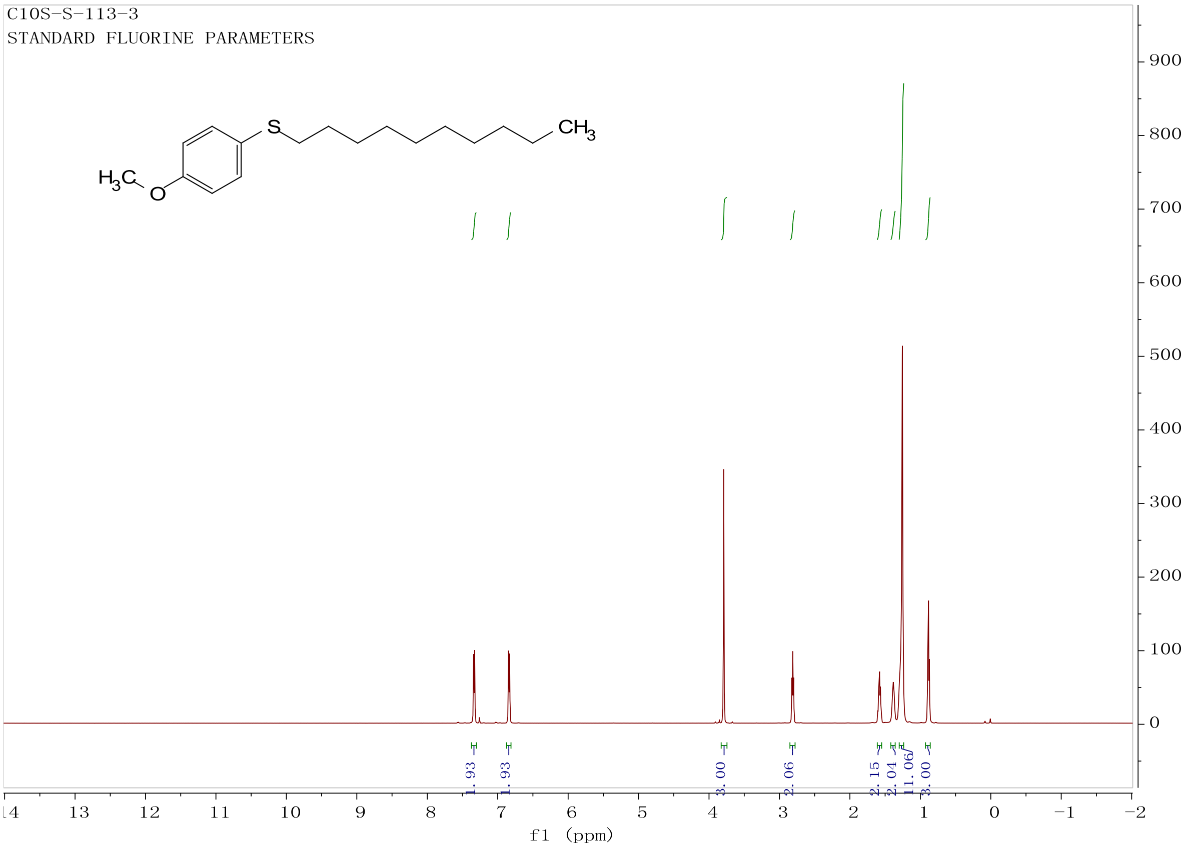
C10S-S-113-3
STANDARD FLUORINE PARAMETERS
S
CH3
H3C
O
1.93
1.93
3.00
2.06
2.15
2.04
11.06
3.00
f1 (ppm)

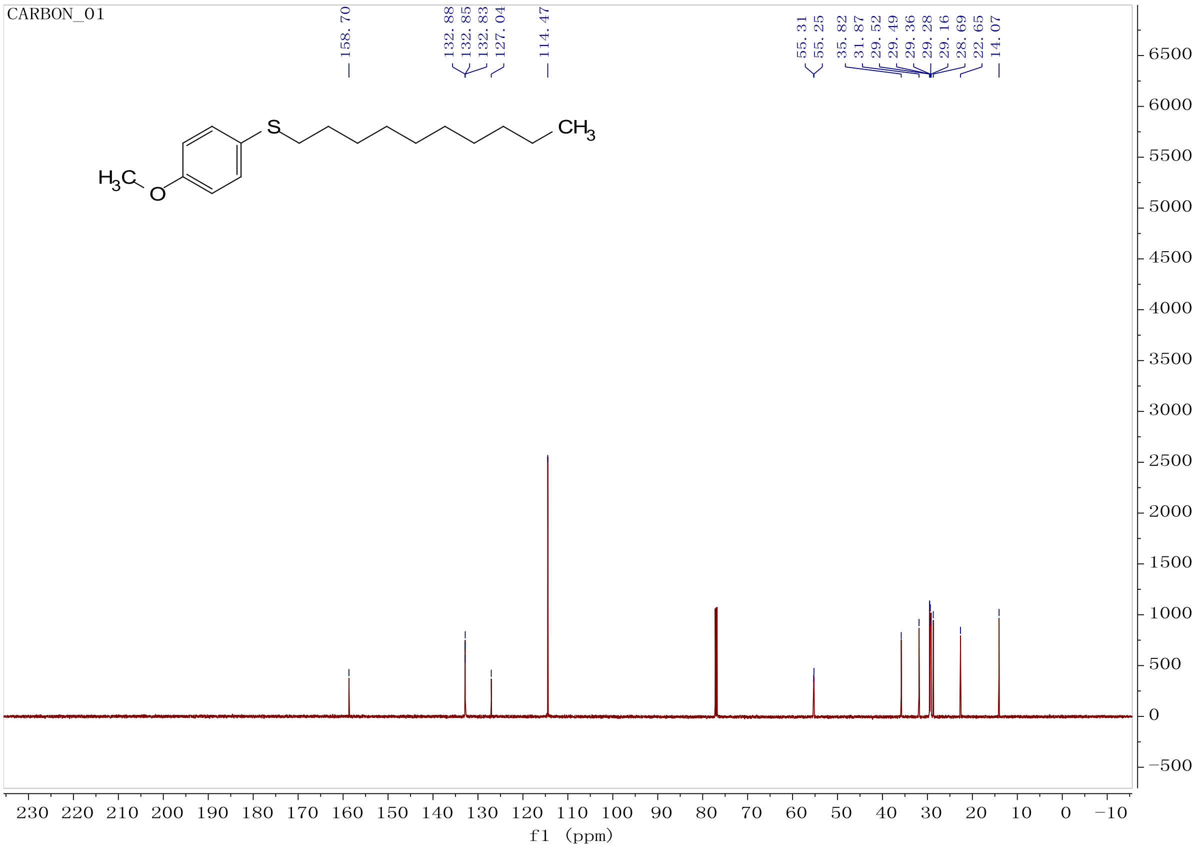
CARBON_01
158.70
132.88
132.85
132.83
127.04
114.47
55.31
55.25
35.82
31.87
29.52
29.49
29.36
29.28
29.16
28.69
22.65
14.07
S
CH3
H3C
O
f1 (ppm)

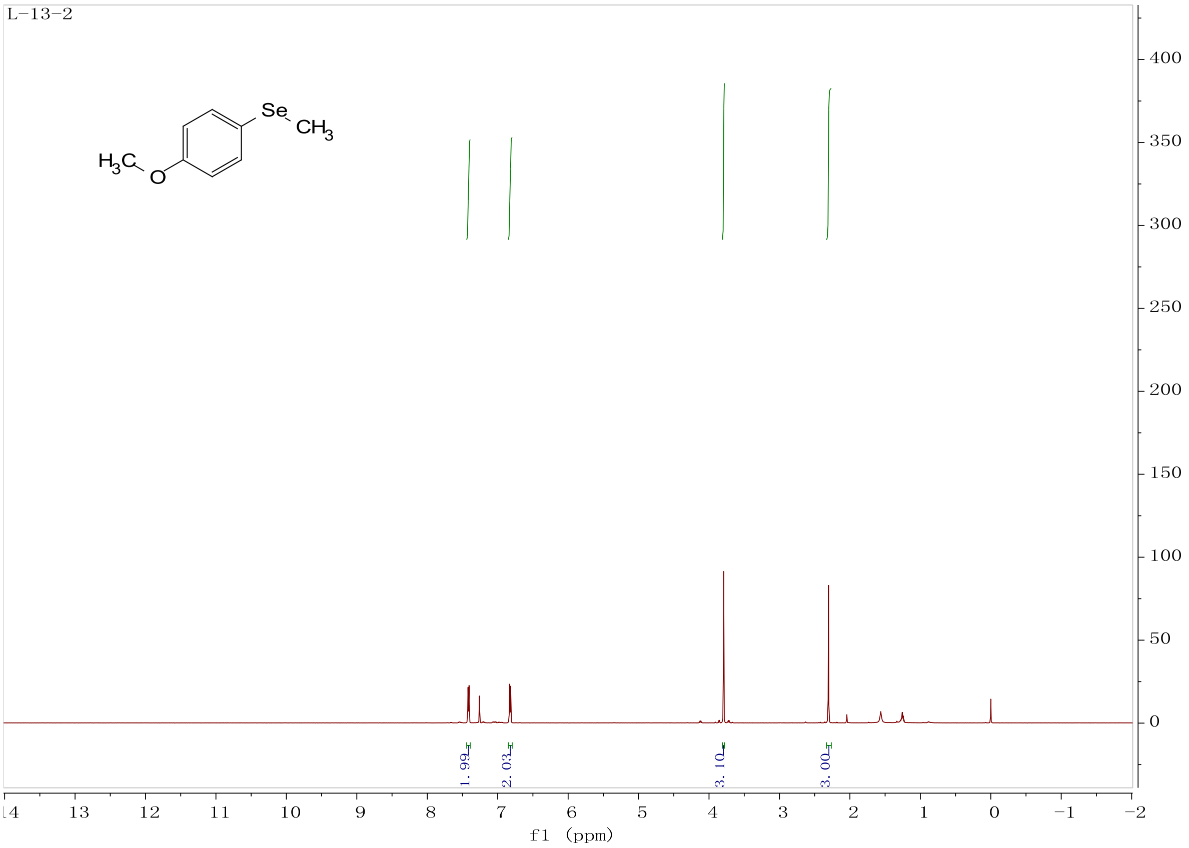
L-13-2
Se
CH3
H3C
O
1.99
2.03
3.10
3.00
f1 (ppm)

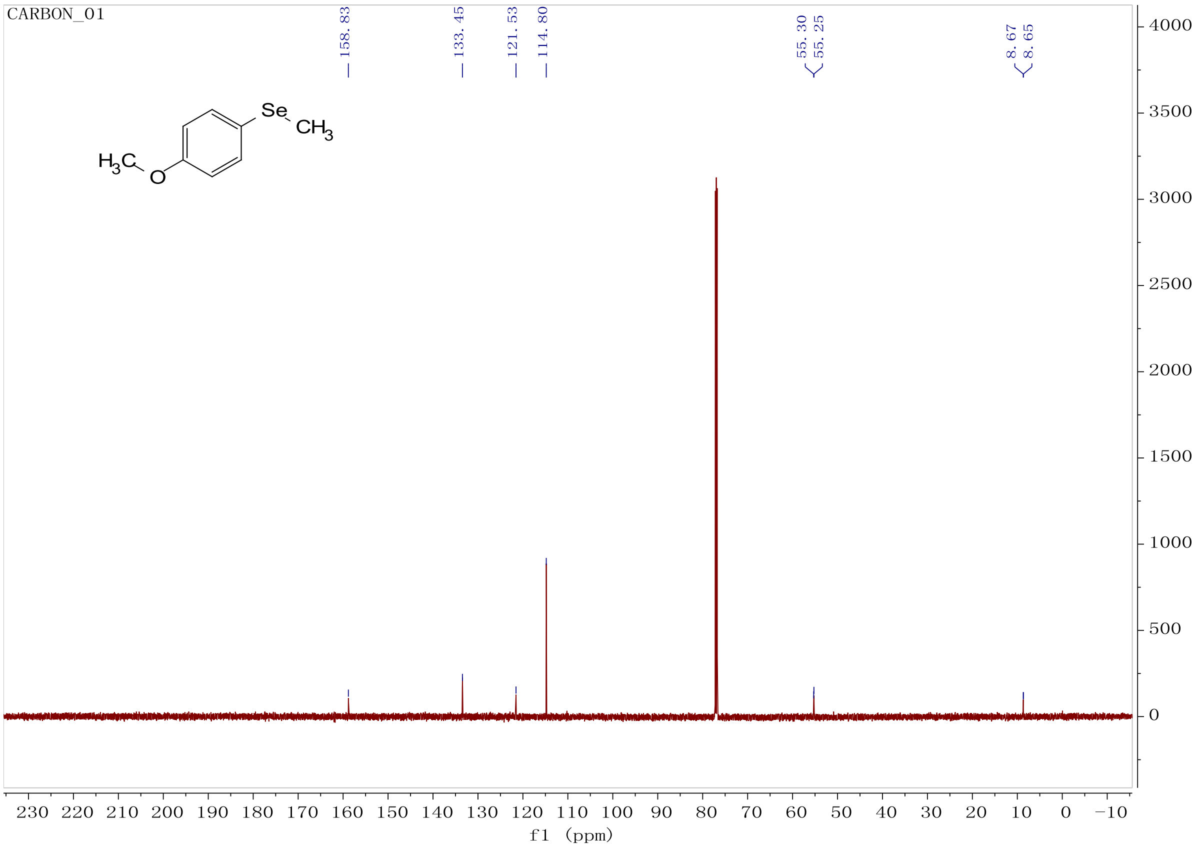
CARBON_01
158.83
133.45
121.53
114.80
55.30
55.25
8.67
8.65
Se
CH3
H3C
O
f1 (ppm)

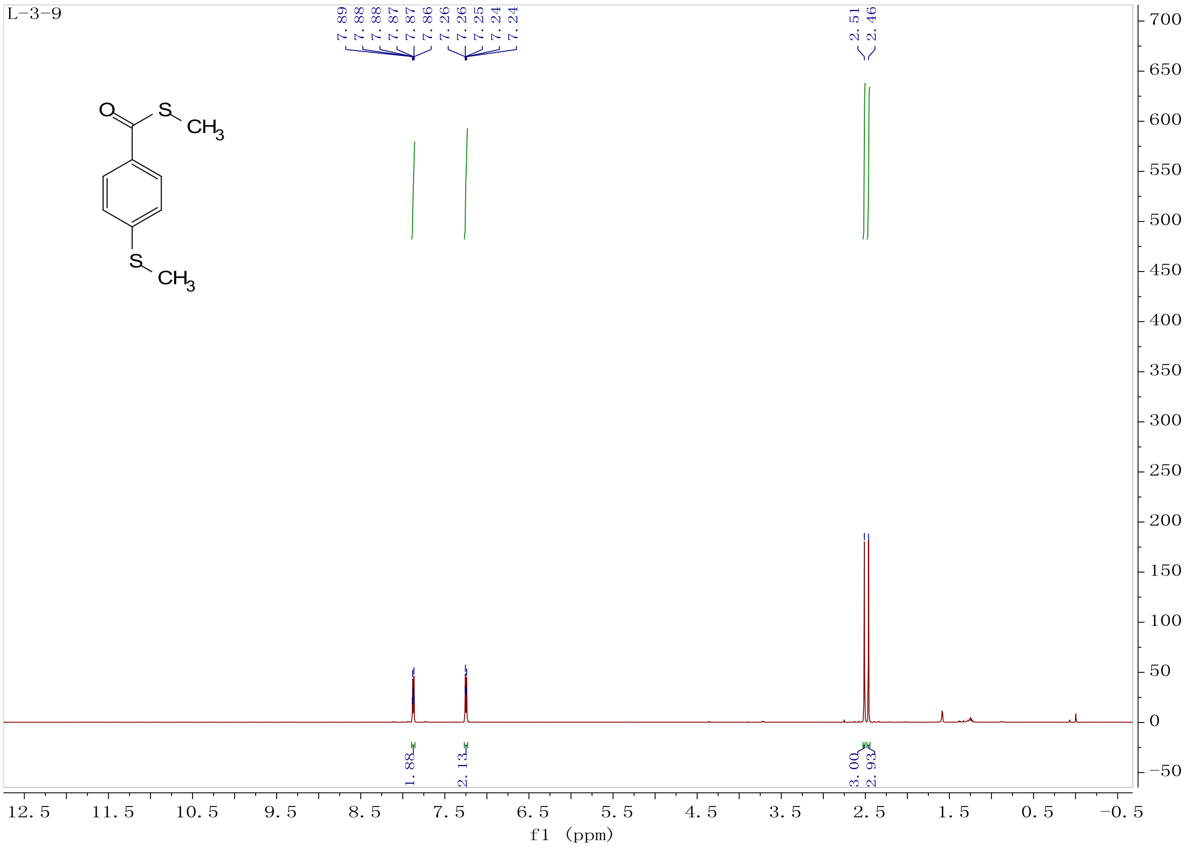
L-3-9
7.89
7.88
7.88
7.87
7.87
7.86
7.26
7.26
7.25
7.24
7.24
2.51
2.46
1.88
2.13
3.00
2.93
f1 (ppm)

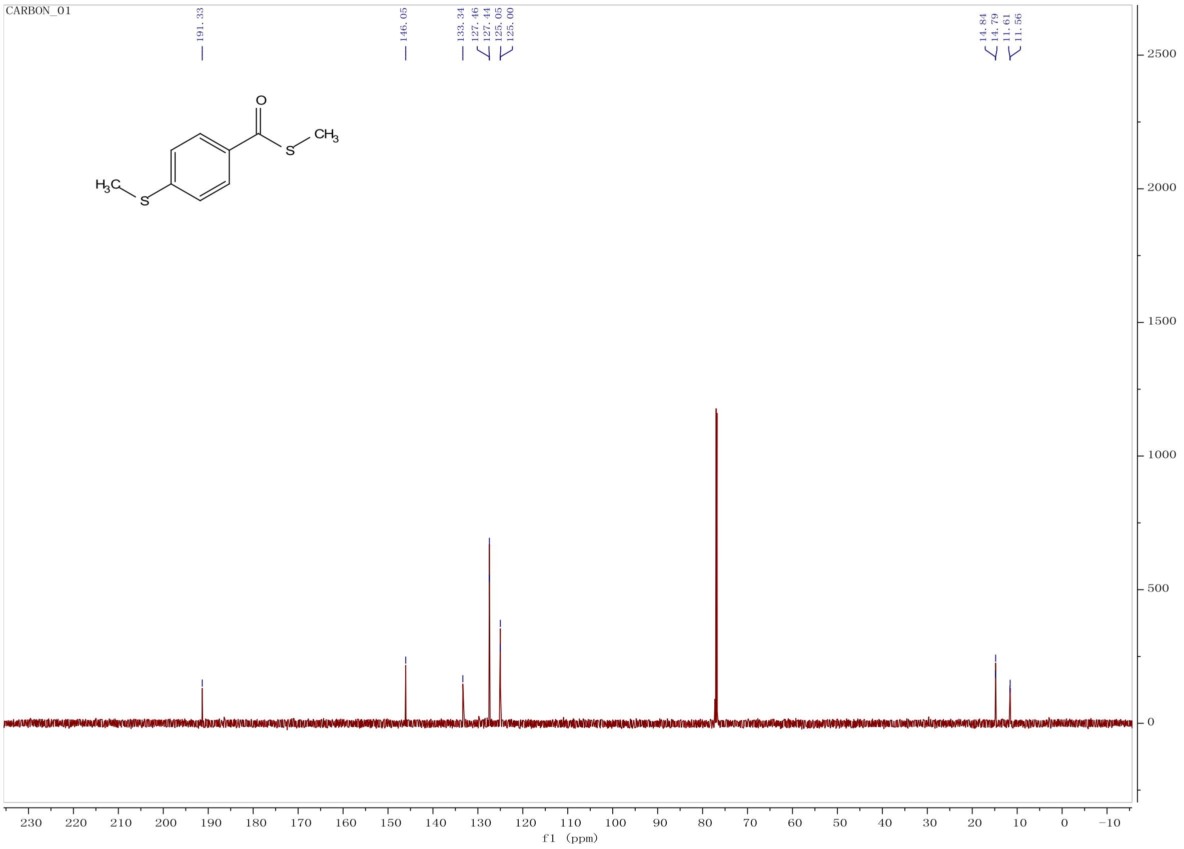
CARBON_01
191.33
146.05
133.34
127.46
127.44
125.05
125.00
14.84
14.79
11.61
11.56
f1 (ppm)
